# An overview on animal/human biomass-derived carbon dots for optical sensing and bioimaging applications†

**DOI:** 10.1039/d3ra06976a

**Published:** 2023-12-01

**Authors:** Prashant Dubey

**Affiliations:** a Centre of Material Sciences, Institute of Interdisciplinary Studies (IIDS), University of Allahabad Prayagraj-211002 Uttar Pradesh India pdubey@allduniv.ac.in pdubey.au@gmail.com

## Abstract

Over the past decade, carbon dots (CDs) have emerged as some of the extremely popular carbon nanostructures for diverse applications. The advantages of sustainable CDs, characterized by their exceptional photoluminescence (PL), high water solubility/dispersibility, non-toxicity, and biocompatibility, substantiate their potential for a wide range of applications in sensing and biology. Moreover, nature offers plant- and animal-derived precursors for the sustainable synthesis of CDs and their doped variants. These sources are not only readily accessible, inexpensive, and renewable but are also environmentally benign green biomass. This review article presents in detail the production of sustainable CDs from various animal and human biomass through bottom-up synthetic methods, including hydrothermal, microwave, microwave-hydrothermal, and pyrolysis methods. The resulting CDs exhibit a uniform size distribution, possibility of heteroatom doping, surface passivation, and remarkable excitation wavelength-dependent/independent emission and up-conversion PL characteristics. Consequently, these CDs have been successfully utilized in multiple applications, such as bioimaging and the detection of various analytes, including heavy metal ions. Finally, a comprehensive assessment is presented, highlighting the prospects and challenges associated with animal/human biomass-derived CDs for multifaceted applications.

## Abstract 2

Animal/human-derived biomass has shown a significant potential for the sustainable/green synthesis of luminescent carbon dots with wide applicability in the fields of sensing, bioimaging and many more.

## Graphical abstract

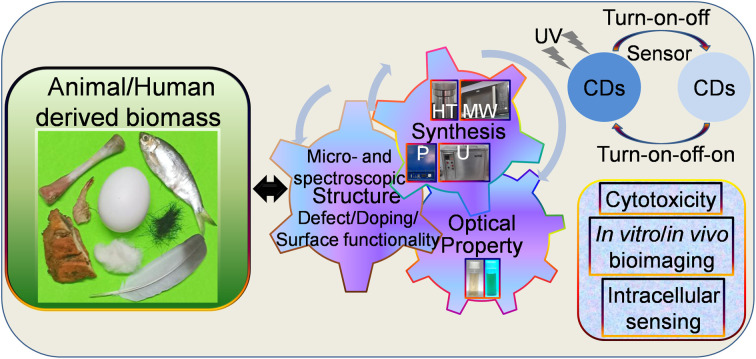

## 1 Introduction

**Fig. 1:**
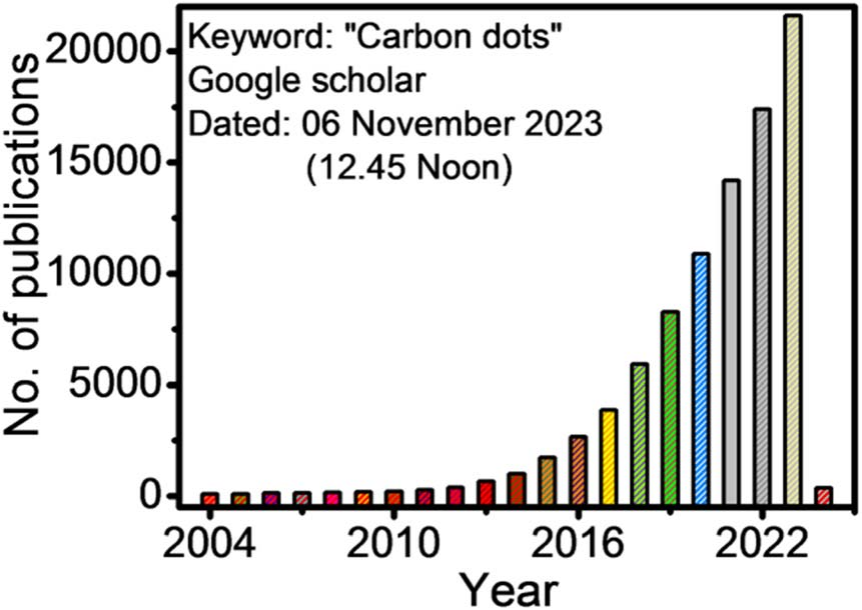
The number of publications according to the Google Scholar search since 2004 (keywords: carbon dots).

**Fig. 2:**
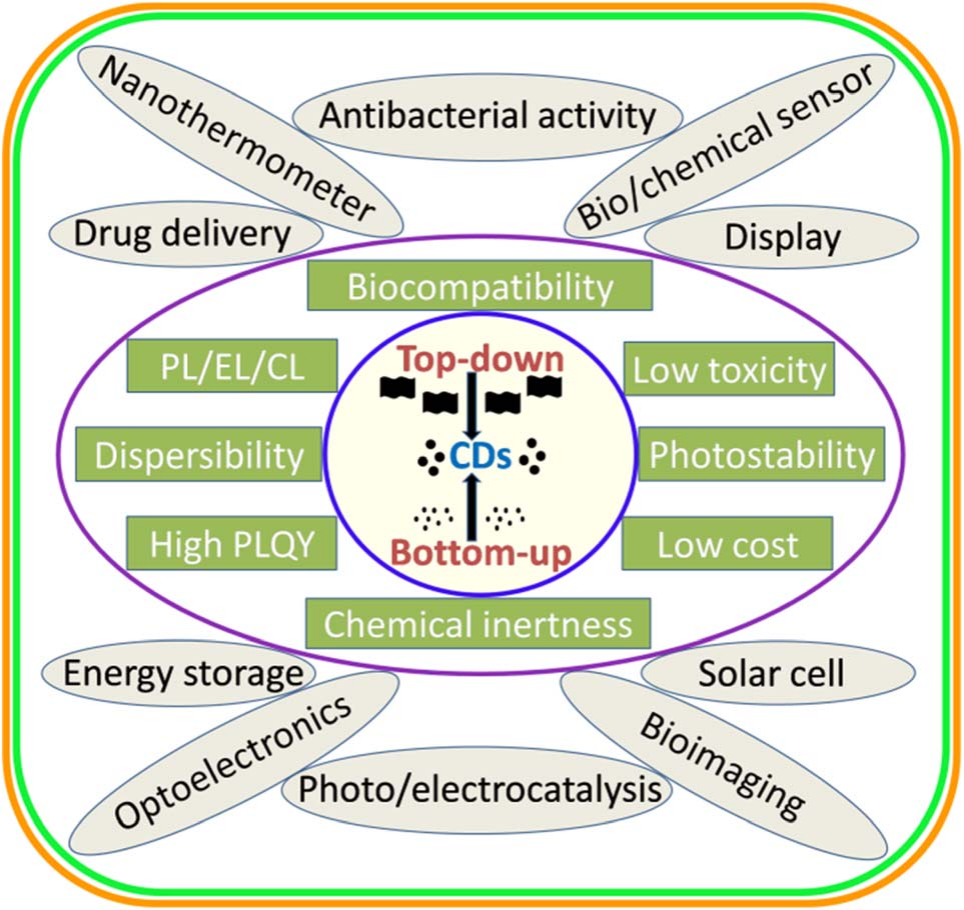
A schematic illustration of the broad synthetic approaches, various physiochemical properties, and applications of CDs.

Carbon dots/quantum dots (CDs/CQDs) represent a family of zero-dimensional quasi-spherical carbon nanoparticles, which are typically less than 10 nm in size and primarily composed of a carbogenic backbone together with hydrogen (H), oxygen (O), and nitrogen (N)-containing surface functionalities.^1,2^ Owing to their different structures and surface chemistry, CDs can be broadly categorized as carbon nanodots (CNDs), polymer carbon dots (PCDs), and graphene quantum dots (GQDs).^3,4^ After the serendipitous discovery of nanosized CDs in 2004 during the purification of single-walled carbon nanotubes,^5^ they have attracted significant research attention in the scientific community as evidenced in Fig. 1. In 2006, Sun *et al.* observed bright luminescent emissions upon the surface passivation of acid treated carbon particles (obtained from laser ablation) by poly(ethylene glycol) (PEG), which, for the first time, were denoted as CDs.^6^ Since then, these emerging materials have become a rising star^7^ owing to their outstanding physiochemical properties (Fig. 2), such as multicolor photoluminescence (PL),^8,9^ chemical inertness,^10^ and resistance to photo-bleaching.^11^ Additionally, the aqueous/organic solvent dispersibility, high quantum yield (QY), low toxicity, good biocompatibility, and relatively inexpensive source for the low-cost, green synthesis of CDs further amplified their interest as an economical and environmentally sustainable class of fluorescent nanomaterials.^7,9,12–15^ Consequently, the utility of CDs have been proven for diverse applications, including solar cells,^16^ optoelectronic devices,^17^ biosensing^18^ and chemical/metal sensing,^19,20^ antibacterial agents,^21^ photocatalysis and electrocatalysis,^22^ displays,^23^ nanothermometers,^24^ drug delivery,^25^ energy storage,^26^ and live cell bioimaging^27^ (Fig. 2). It has been demonstrated that synthetic methods, reaction parameters, and starting precursors are highly responsible for the specific characteristics of CDs.^7,28,29^ Besides the conventional chemical precursors, there has been extensive research on the synthesis, properties, and applications of natural biomass/waste source-derived CDs as evidenced by the large number of review articles.^30–55^

Most of the previous review articles described the synthesis, structural/optical properties, and different applications of biogenic CDs derived from diverse natural/waste sources, such as plant components (leave, stem, root, *etc.*), vegetables, fruits, foods, beverages, and wastes (agricultural, industrial, food, *etc.*), but rarely considered animal/human-derived precursors. For instance, Sharma *et al.*^30^ discussed a variety of green source-derived CDs for sensing and bioimaging applications. Bhandari *et al.*^32^ presented the optoelectronic applications of CQDs/GQDs derived from various biomolecules including natural biomass. Meng *et al.*^33^ highlighted the current developments in biogenic CDs synthesized from diverse natural sources. Fan *et al.*^34^ focused on food waste-derived CQDs for food safety detection. Sekar *et al.*^37^ reviewed the heavy metal ion detection capability of CNDs derived from various green resources. Desmond *et al.*^40^ critically overviewed biomass-based CQDs for cancer therapy. Perumal *et al.*^41^ described the various applications of CDs derived from plant sources. The current developments in biomass-based CDs were also reviewed by Wareing *et al.*^42^ Chahal *et al.*^43^ discussed green-synthesized CDs from natural/non-toxic chemicals. Shahraki *et al.*^46^ presented the multifaceted applications of CDs particularly obtained from food waste. Ahuja *et al.*^47^ outlined the energy and bioremediation applications of CQDs obtained from biomass waste. Kaur *et al.*^48^ discussed the application of fruit waste-derived CDs in bioimaging. Manikandan *et al.*^49^ discussed the environmental applications of green CQDs derived from plant/agro-industrial waste. Xiang *et al.*^50^ critically reviewed CDs obtained from biomass/waste for biological and environmental applications. Fan *et al.*^52^ described biomass-derived CDs for sensing applications. Recently, Singh *et al.*^54^ included CDs synthesized from a variety of biomass for the detection of hazardous ions. In another recent review article, Fang *et al.*^55^ presented an overview of biomass-derived CDs, focusing on their preparation, property, and various applications. However, although there are numerous review articles related to biogenic CDs, a comprehensive overview of CDs derived from animal/human sources is limited in the literature. Natural biomass acquired from animal/human has attracted significant interest given that it is a minimal chemical intake, abundant, low-cost, and renewable green source. Moreover, the conversion of animal/human waste into value added products is an economical and sustainable approach to realize the waste-to-wealth initiative. Therefore, the assessment of animal/human biomass-derived CDs together with the recent achievements is paramount for future developments.

This review explicitly aims to present an overview of CDs synthesized from animal and human biomass for sensing and bioimaging applications. Initially, the diverse synthetic strategies, formation mechanisms, and various modifications associated with CDs are outlined. Furthermore, the comprehensive correlation/development of the size, QY, and elemental compositions of CDs derived from animal/human-based precursors using various preparation methods is presented with their structural and property description/relationship. Based on the preceding knowledge, the utilization of these animal/human biomass-derived CDs for metal ion/other analyte sensing and fluorescent biomarker applications is discussed together with a brief description of their other applications. Finally, this article is concluded by highlighting the achievements of these CDs in addition to identifying the future prospects of animal/human biomass as renewable resources and attempting to address the challenges/limitations in their practical implementation.

## 2 Diverse strategies for the synthesis of CDs

**Fig. 3:**
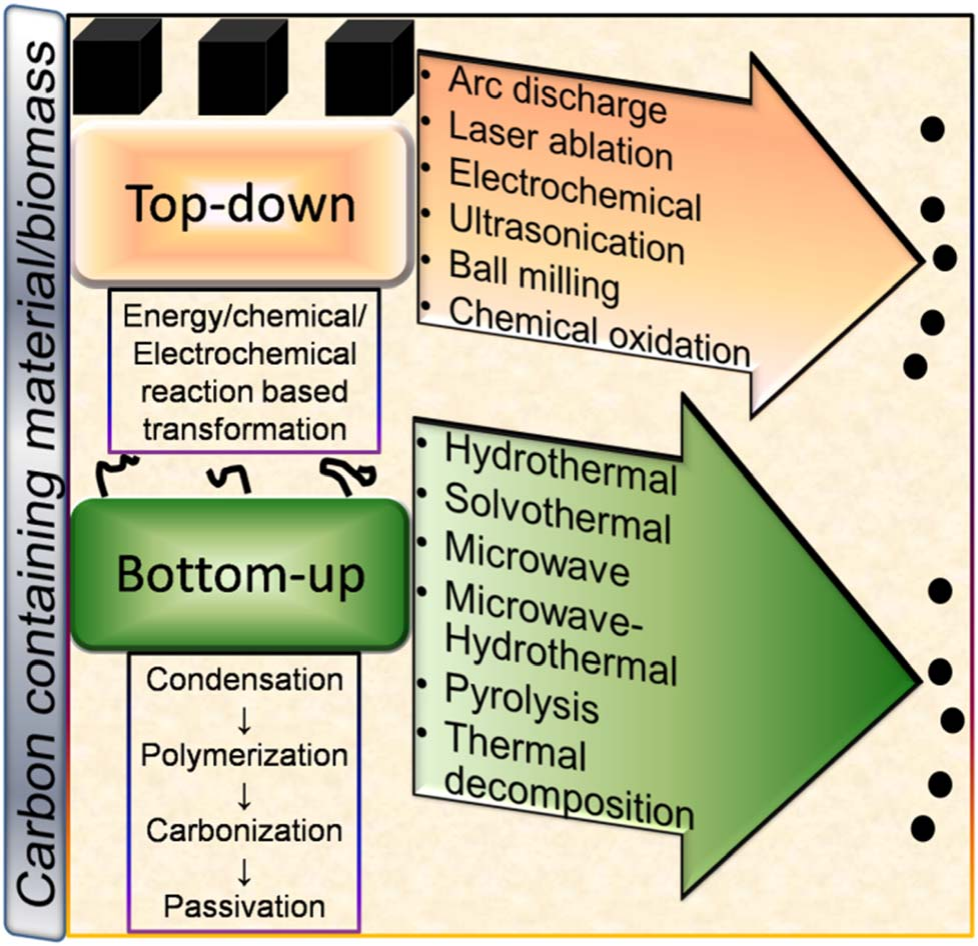
A schematic illustration of the various top-down and bottom-up techniques for the fabrication of CDs and their formation mechanism.

Broadly, CDs can be synthesized either *via* the top-down or bottom-up approach using chemical or natural precursors as starting materials. A schematic illustration of the various top-down and bottom-up synthetic routes is shown in Fig. 3. Both synthetic strategies have their own merits/demerits and have been developed with time for the preparation of narrow-sized CDs with excellent optical properties.

### 2.1 Top-down approach

This approach involves the breaking of bigger carbon precursors into nano-sized carbon particles. Here, the synthesis of CDs using various top-down methods is highlighted with specific examples.

#### 2.1.1 Arc discharge method

The generation of a high-energy electric arc by placing two electrodes in close proximity under an applied potential drives the evaporation of the electrode material to produce CDs or other carbon nanostructures. Chao-Mujica *et al.*^56^ applied submerged arc discharge in water to synthesize CDs from a graphite rod. The size of the fluorescent CDs was found to be in the range of 1 to 5 nm with an appreciable QY (∼16%). It was proposed that the sublimed carbon vapor together with the exfoliated graphite/graphene fragments function as growth centers for the heterogeneous nucleation of polyhedral carbon nano-onions (CNOs) and large disordered polyhedral particles, which transformed into CDs and graphene oxide *via* water vapor oxidation.

#### 2.1.2 Laser ablation method

An intense laser source (pulse) is also used to fragment micrometric carbonaceous solids into CDs.^57,58^ For instance, laser ablation of a carbon target (graphite) in ethylenediamine (EDA) and polyethyleneimine (PEI) resulted in the formation of crystalline CDs (1–3 nm) together with amorphous nanoparticles. Interestingly, a significant difference in the optical properties of CDs (extracted after dialysis) was observed in comparison to the remaining particles (retentate) for laser ablation conducted in EDA, indicating the presence of free fluorescent molecules. Consequently, the QY of EDA-CDs reached up to 10.4%, while PEI-CDs showed a low QY (2.5–3.3%).^58^

#### 2.1.3 Electrochemical method

The electrochemical synthesis of CDs is another top-down approach, which is based on redox chemistry induced by applying a potential between two electrodes (cathode and anode).^59^ The first electrochemical synthesis of CDs (average diameter: 2.8 ± 0.5 nm) was reported in 2007, where multiwalled carbon nanotube (MWCNT)-coated carbon paper was used as the working electrode together with Pt-wire (counter electrode) and Ag/AgClO_4_ (reference electrode).^60^ In a recent report, Zhao *et al.*^61^ synthesized N,S-CDs through an electrochemical method for the fabrication of a flexible fluorescent film and information encryption application. An optimized potential (60 V, 3 h) was applied on two graphite electrodes (anode and cathode) together with phenylenediamine (N source) and thioacetamide (S source) to yield red/blue/green-emitting N,S-CDs. The green-emitting N,S-CDs showed a significantly higher absolute QY (12.99%) compared to their undoped counterpart (0.49%), which is ascribed to the narrowed band gap of CDs *via* –NH_2_ functionalization and increased excited-state electron transition related to the S atom of the C–S bonds.

#### 2.1.4 Ultrasonication method

The breakdown of large carbonaceous precursors *via* high-energy ultrasonic waves is another simple and convenient top-down method, which has been explored for the synthesis of CDs.^62^ For example, the ultrasonic treatment (37 kHz) of CNOs in an H_2_SO_4_ : HNO_3_ mixture (3 : 1) for 2 h followed by reflux treatment (100 °C, 2 h) resulted in the formation of green-emitting CDs (average size: 6.4 ± 1.8 nm) with excitation-wavelength independent (EWID) PL.^63^ Xu *et al.*^64^ synthesized biogenic CDs *via* the ultrasonic treatment (320 W, 80 kHz, 3 h) of kiwifruit juice in the presence of different additives such as ethanol, acetone, and EDA. The CDs obtained with acetone additive showed a smaller average diameter (4.8 nm) compared to that with ethanol (6.7 nm) and EDA (6.9 nm) additives. Although the size effect of the CDs was not very pronounced in the different additives, the absolute QY of the acetone additive-derived CDs was much higher (∼10%) compared to that of the other two CDs (∼3.7/∼2.4% for ethanol/EDA additive-derived CDs), which was explained by the occurrence of electron–hole recombination mainly from the surface trap sites.

#### 2.1.5 Ball milling method

The breaking/milling of large-sized precursors by applying the kinetic energy of high-speed rotating balls has been considered a green mechanochemical approach for the large-scale synthesis of CDs.^65^ Mechanical ball milling of a mixture of cellulose and Mg with ZrO_2_ balls (360 rpm, 28 h) resulted in the formation of Mg-doped CDs (average diameter: 4.8 nm, QY: 9.45%), which exhibited potential as an Fe^3+^ sensor.^66^ Jeong *et al.*^67^ applied wet ball milling on spent coffee grounds to produce CDs (<2.0 nm). The surface property of the CDs was controllably tuned through the incorporation of oxygen/nitrogen-containing small molecules during the milling process, which improved the sensitivity and selectivity of the functionalized CDs for the detection of Fe^3+^ in aqueous medium.

#### 2.1.6 Chemical oxidation method

The breakdown/etching of a bigger carbon source *via* chemical oxidation is another popular and simplistic top-down approach for the synthesis of CDs. For instance, He *et al.*^68^ demonstrated a facile and low-cost acid oxidation strategy for the production of a high-yield CD-based pickling solution from various saccharides (glucose, fructose, soluble starch, and saccharose; green C source) and concentrated H_2_SO_4_ (acid oxidant). The CDs containing pickling solution were obtained in a very short time (∼3 min), which showed excellent corrosion inhibition efficiency for Q235 carbon steel. Saikia *et al.*^69^ fabricated self-doped N,S-CDs from coal feedstock using 30% hydrogen peroxide (H_2_O_2_) as an oxidant with the assistance of ultrasonication. The resulting CDs (average size: 6 nm, QY: 9.35/16.96%) were used as a plant growth promoter with low phytotoxicity on plant metabolism.

### 2.2 Bottom-up approach

The preparation of nano-sized CDs *via* the condensation of small carbon precursors can be broadly termed the ‘bottom-up’ route. Some common bottom-up methods for the synthesis of CDs include hydrothermal (HT), solvothermal (ST), microwave (MW), microwave-hydrothermal (MW-HT), pyrolysis, and thermal decomposition (TD) (Fig. 3).

#### 2.2.1 HT method

The HT method is one of the extensively explored synthetic techniques to achieve CDs from chemical compounds^70,71^ and natural precursors.^72,73^ The reaction parameters such as temperature and reaction time have been found to be crucial to tune the size, yield and fluorescence property of the synthesized CDs. Moreover, it is an inexpensive and readily available method with broad prospects. Recently, Hou *et al.*^73^ used HT conditions (200 °C, 4 h) and an egg white precursor to synthesize CDs (2–5 nm) for the fabrication of a CDs/layered zirconium phosphate composite. Combined with the synergy of two components, the composite showed the maximum Fe^3+^ adsorption capacity of 93.01 mg g^−1^ and detection ability in real environmental samples.

#### 2.2.2 ST method

Similar to HT, the condensation of the starting precursors can also occur in non-aqueous medium. For example, the facile ethanol-assisted ST treatment of maize starch together with Na_2_HPO_4_·12H_2_O, NaH_2_PO_4_·2H_2_O, and urea resulted in the formation of N,P-codoped CDs (average diameter: ∼2.5 nm) with a QY as high as 76.5%.^74^ Hao *et al.*^75^ demonstrated a one-pot ST approach to synthesize CDs using folic acid (FA; 160 °C, 1 h) as a carbon source. The synthesized CDs possessed a narrow size range (2.8–3.2 nm) together with an appreciable QY under 360 nm excitation (31.2%), which were applied to fabricate a paper-based sensor for the detection of Hg^2+^ and a non-toxic nanoprobe for cellular imaging.

#### 2.2.3 MW method

An energy efficient and low time-consuming MW-assisted method has also been explored with chemical and biomass precursors to derive functional CDs.^76,77^ Li *et al.*^78^ applied MW treatment (3 min) to a mixture of carbonized laver (biomass) and PEG to yield blue-emitting CDs for the detection of oxytetracycline. In another recent report, citric acid (CA) and tris base were used as starting precursors to synthesize CDs *via* the MW method. The fluorescent CDs showed potential for the detection of Mn^2+^ and tartrazine and anti-counterfeiting applications.^79^

#### 2.2.4 MW-HT method

HT treatment of the starting precursors under MW irradiation has also been explored as a bottom-up process in the synthesis of CDs. In contrast to the typical HT method, MW-HT treatment achieves a desired reaction temperature in a shorter time. For instance, an MW-HT method (100 °C, 5 min) was applied on empty fruit bunch biochar to produce CDs with an average diameter of ∼4.5 nm. The obtained CDs were also implemented for the fluorescence quenching-based detection of Cu^2+^.^80^

#### 2.2.5 Pyrolysis method

The direct pyrolysis of carbonaceous precursors has been considered a simple and facile bottom-up approach to synthesize CDs. It was observed that CA can be transformed into fluorescent CDs (QY: 29%) with the optimal carbonization temperature/time (160 °C/50 min).^81^ The solvent-free pyrolysis of starch at 300 °C yielded narrow-sized CDs (1.3–2.3 nm) with a QY as high as 21%. The synthesized CDs were also applied for the fluorometric detection of Ru^3+^ in aqueous solution.^82^

#### 2.2.6 TD method

TD is slightly different from the pyrolysis method in the sense that the starting precursors in liquid/semi-liquid/solution state can be carbonized at elevated temperature. For example, an aqueous extract of *Miscanthus* grass together with EDA was thermally decomposed at 180 °C for 4 h to produce N-CDs (average size: ∼4.6 nm, QY: 11.7%), which were also applied as a nanoprobe for Fe^3+^ sensing.^83^ In another report, tender coconut water was directly heated at 100 °C to synthesize CDs for the detection of Zn^2+^.^84^

## 3 Formation mechanism of CDs

The formation mechanism of CDs follows different routes in the top-down and bottom-up synthetic approaches.

### 3.1 Top-down pathway

In the top-down route, graphite,^56,58^ MWCNTs,^60^ and other bulk carbonaceous sources (*e.g.*, activated carbon,^85^ activated carbon fiber,^86^ and coal feedstock^69^) have been utilized as the starting source, which can be broken/cleaved into tiny CDs by employing physical, electrochemical or chemical oxidation method. The breaking/etching of inherent chemical bonds associated with bulk carbon drives the formation of CDs. This bulk to nano transformation can be induced either by high energy (arc discharge, laser ablation, ultrasonication-based acoustic cavitation, *etc.*) or chemical/electrochemical reaction (Fig. 3). Although the physical top-down strategy is less common for biomass-derived CDs, some researchers employed the chemical oxidation-based top-down formation route and natural/waste resources such as coal feedstock,^69^ candle soot,^87^ coal tar pitch,^88^ and carbonized olive solid waste^89^ to produce surface-passivated CDs. The harsh oxidizing environment (HNO_3_, H_2_SO_4_/HNO_3_, H_2_O_2_, *etc.*) eventually disrupts the bonding arrangements of bulk carbon, resulting in its breakdown into CDs. He *et al.*^90^ demonstrated the one-step transformation of carbon nanotubes (CNTs) into CDs *via* a thiol–ene click reaction. The sp^2^ carbon of CNTs reacted with thiomalic acid under reflux condition to produce –COOH-functionalized CDs for bioimaging application. The formation mechanism of coal-derived N,S-CDs is based on the breaking of the organic carbon linkage (binding bridge of crystalline fragments in the microstructure of coal) *via* ˙OH radicals (produced from H_2_O_2_). The oxidant can reach inside the interlayers of the graphitic structure (abundantly present in coal) to facilitate the disintegration of the bridging bonds such as –CH_2_–CH_2_–, –CH_2_–O–, –O–, and –S–. Moreover, acoustic cavitation induced by ultrasonication favours the breaking of the bonds between the graphitic structure and polycyclic aromatic fragments. These aromatic components eventually fragment into C_2_ units to form N,S-CDs *via* polymorphic reaction.^69^

### 3.2 Bottom-up pathway

The bottom-up formation of CDs relies on the solution-phase condensation/carbonization of small carbon-containing molecules. Organic molecules (conjugated or non-conjugated) or natural biomass can be used to produce CDs *via* the bottom-up route.^91,92^ The involvement of four stages (condensation, polymerization, carbonization, and passivation) is commonly speculated in the formation of CDs from small organic precursors (Fig. 3).^91,92^ Initially, the starting organic molecules get condensed into a small chain-like structure, which subsequently polymerizes to form polymer-like aggregates or carbon clusters. Subsequently, the carbonization/aromatization of polymer-like aggregates generates a carbonaceous core (amorphous/graphitic) at elevated temperature, which has an outer surface containing various functional groups.^91,92^ The reaction temperature/time plays a crucial role in determining the sp^2^/sp^3^ domains of CDs in the carbonization step.^91,92^ He *et al.*^93^ described the formation mechanism of MW-synthesized N-CDs, involving inter-and/or intra-molecular dehydration of an amide-rich organic precursor, followed by polymerization and carbonization to produce a carbon core possessing oxygen-containing functional groups. It was observed that the amount of core backbone increased with an increase in temperature and time, which is ascribed to the gradual transformation of the functional groups into a carbogenic core. Cao *et al.*^94^ proposed the formation mechanism of N-CDs derived from *o*-phenylenediamine using the ST method. Initially, 2,3-diaminophenazine (oxidized/cyclised product of the precursor) aggregates (H-bonding and π interaction) into a polymeric cluster, which creates a core–shell type of preliminary structure in the subsequent carbonization step. With time, the shells collapse with the evolution of the graphitised core in the form of N-CDs. Recently, Mohammed *et al.*^95^ investigated the effect of reaction time on the formation mechanism of N-CDs derived from 4-aminoantipyrine under HT condition. The spectroscopic results indicated the reduction and enhancement of the peak intensities in the aromatic and aliphatic/carbonyl group region, respectively, with the extension of the reaction time, indicating the structural changes during the synthesis process. These changes are correlated with the π–π interactions (non-covalent) of the benzene rings present in the precursor molecules. Moreover, the attachment of polar functional groups to the surface of N-CDs was accompanied by the hydrolysis of the pyrazole ring present in 4-aminoantipyrine.

Generally, biogenic CDs follow the bottom-up formation mechanism due to the abundance of organic molecules in the starting precursors. Although it is difficult to track the exact mechanism of these CDs due to the complex composition of their starting precursor, the decomposition of biomass into active components, followed by their polymerization, condensation, carbonization, and nucleation are the commonly accepted formation steps.^96,97^ For example, *Shewanella oneidensis* MR-1 bacterial cells first decomposed to macromolecules (polysaccharides/proteins/lipids, *etc.*), followed by hydrolysis to produce a large number of small organic molecules (saccharides/peptides/amino acids/hydrocarbon *etc.*). These small organic fragments get polymerized, condensed, and carbonized in the subsequent steps to form doped CDs due to nuclear burst.^97^

## 4 Modifications of CDs

The modification of CDs is vital for the improvement of physiochemical features, particularly their optical properties. Recently, Fan *et al.*^98^ reviewed the metal ion sensing application of modified CDs. The inherent modifications in CDs can be achieved either by heteroatom doping or surface functionalization.

### 4.1 Heteroatom doping

Various heteroatoms can be accommodated in the interior core or surface of CDs for the enhancement of their emission properties. Heteroatom-doped CDs usually possess abundant surface defects and active sites, resulting in the modification of their electronic structures *via* tuning of their initial band gap or formation of new energy levels.^3,42^ Consequently, doped CDs show better possibility for diverse applications due to the improvement in their optical characteristics and QY.^3,42,98^ N is one of the most common doping elements (atomic radius of C/N: 0.0914/0.092 nm), which is incorporated in CDs to improve their fluorescence properties.^99^ A green precursor (glucose) was HT treated in the presence of NH_3_ to produce N-CDs. The optimized N-CDs (N content: 10.94%) showed a significantly high PL emission with a QY of 9.6% compared to their undoped counterpart (QY: <1%), which is ascribed to the modifications in the surface/core electronic states of N-CDs. N-CDs were also used for the sensitive and selective detection of Cr^6+^.^100^ Sulfur (S) is another heteroatom that can be incorporated in CDs to enhance their optical properties. For instance, S-CDs synthesized from a mixture of ascorbic acid (AA) and thioglycolic acid (TGA) using the HT method (180 °C, 6 h) showed a QY as high as 32.07%. Moreover, they could also recognize Fe^2+^/Fe^3+^ from a real oral ferrous gluconate sample.^101^ N and S elements are also codoped in biogenic CDs to enhance their fluorescence property and sensing application.^102^ CDs doped with boron (B) and phosphorous (P) heteroatoms are also known in the literature. For example, P-doped CDs derived from a trisodium citrate–phosphoric acid (H_3_PO_4_) mixture under HT condition showed a satisfactory QY (16.1%) and Fe^3+^ sensing ability *via* the quenching of their fluorescence signal.^103^ Sadhanala *et al.*^104^ developed B-doped CDs through the ST treatment of catechol (C source) and naphthalene boronic acid (B source). The violet luminescent B-doped CDs (QY: 39.4%) were implemented for the sensitive/selective detection of Mg^2+^. The choice of the natural precursor in the synthesis of doped CDs is advantageous for the self-incorporation of heteroatoms in their structure.^105^ However, strategic doping can be achieved by using heteroatom-containing precursors together with the natural biomass. For example, the HT treatment of frozen tofu, EDA, and H_3_PO_4_ (210 °C, 4 h) produced N,P-codoped CDs, which were applied for sensing and bioimaging.^106^

Wang *et al.*^107^ firstly demonstrated the modification of CDs with metal ions (Mn^2+^) *via* the formation of Mn^2+^–coordination functional knots. Since then, a large number of metal ions has been used as doping agents for modifying the electronic state, charge density, and physiochemical properties of CDs.^108^ For example, the simple room-temperature synthesis of Si-CDs was achieved using 5-sulfosalicylic acid and 3-aminopropyl triethoxysilane (APTES), which were also used for the detection of Hg^2+^.^109^ The HT reaction between ethylenediamine tetraacetic acid disodium salt and FeCl_3_·6H_2_O yielded Fe-CDs with an Fe content as high as 13 wt%. Consequently, the Fe-CDs showed 2.6-times greater photocatalytic reduction ability (CO_2_ to methanol) compared to the undoped CDs.^110^ Zhang *et al.*^111^ used the traditional Chinese herb mulberry together with MgCl_2_·6H_2_O to synthesize Mg-CDs through the HT technique (200 °C, 10 h). The Mg-CDs were applied for *in vitro* osteoblastic differentiation and matrix mineralization.

### 4.2 Surface functionalization

The surface of CDs contains abundant functional groups, which can be modified with functional molecules either *via* covalent chemistry or non-covalent interaction.^112^ Covalent modification involves the direct binding of additional molecules to the surface of CDs, whereas π interaction, electrostatic conjugation and van der Waals force direct non-covalent modification. The coupling of the abundant –COOH groups present on the surface of CDs with amine containing compounds *via* an amide linkage is one of the simplest covalent functionalization strategies to improve the fluorescence intensity and amphiphilic character of CDs.^113^ CDs derived from CA and diaminonaphthalene under ST condition showed an excellent QY of 70% ± 10%, which is attributed to the pronounced edge amination of the CDs to reduce the number of defect sites, therefore delaying non-radiative recombination.^114^ The acid-catalyzed esterification reaction between –COOH and –OH-containing compounds is another simple modification strategy. Lactose-derived CDs were modified with mercaptosuccinic acid (MSA) *via* the esterification protocol, which showed an improved QY (46%) in comparison to the unmodified CDs (21%). Furthermore, the MSA-CDs were also evaluated for Ag^+^ sensing application with good sensitivity and selectivity.^115^ Sulfonyl chloride compounds are attached to –NH_2_-containing CDs *via* sulfonylation reaction. For example, amino-rich CDs (chitosan derived) were coupled with 4,4′-bis(1′′,1′′,1′′,2′′,2′′,3′′,3′′-heptafluoro-4′′,6′′-hexanedion-6′′-yl)chlorosulfo-*o*-terphenyl-Eu^3+^ (BHHCT-Eu^3+^) *via* sulfonylation reaction to fabricate a ratiometric fluorescent nanoprobe for the detection of Cu^2+^. Interestingly, the red fluorescence of BHHCT-Eu^3+^ (at ∼615 nm) was quenched in the presence of Cu^2+^ without affecting the blue fluorescence (at ∼410 nm) arising from CDs.^116^ The surface of CDs can also be redesigned with the silane moiety. For example, silylation chemistry was used to covalently functionalize CDs (CA derived) with tetraethyl orthosilicate and APTES, which possessed a large number of –NH_2_ groups for the coupling of TGA-modified CdTe QDs. The resulting ratiometric fluorophore showed excellent Cu^2+^ sensing ability *via* quenching of the fluorescence signal originating from the CdTe QDs.^117^ Suzuki *et al.*^118^ grafted methyltriethoxysilane and 3-glycidyloxypropyltrimethoxysilane on –NH_2_-containing CDs (prepared from CA and EDA using HT method) *via* epoxy-amine reaction, which were used to prepare organic–inorganic hybrid films for solid-state emitting devices. In a recent report, *N*-aminoethyl-γ-aminopropyltrimethoxysilane-functionalized CDs were applied for corrosion inhibition.^119^ Modification of the surface of CDs by copolymerization is advantageous to get a high molecular weight scaffold. For instance, Li *et al.*^120^ used anionic ring-opening polymerization (monomer: glycidol) to conjugate hyperbranched polyglycerol (HPG) on the surface of –OH-containing CDs (derived from α-cyclodextrin *via* the HT process). Although the QY of HPG-*g*-CDs was estimated to be slightly lower (1.2%) than that of bare CDs (1.5%), they exhibited high water dispersibility and low cytotoxicity for cell labelling and imaging.

Besides, CDs are also modified with functional moieties *via* non-covalent interaction. The extended π system on the surface of CDs is advantageous to modify their surface *via* π–π interaction. For example, Si-CDs prepared from glycerol and APTES through the MW method were modified with dopamine (DA) *via* face-to-face π–π interaction. The resulting Si-CDs@DA was applied for the fabrication of an Ag^+^ sensor and intracellular visualization of Ag^+^.^121^ According to the surface functional groups/attached moieties, the CDs can impart surface charge (positive or negative) for the electrostatic interaction of targeting species. For instance, PEI-functionalized CDs were prepared *via* non-covalent adsorption/wrapping of a cationic polymer (PEI), which were further modified with FA *via* electrostatic interaction. The resulting FA-modified PEI-CDs were used as a fluorescent nanoprobe (turn-on, *in vitro*/*in vivo*) to target folate receptor-positive cancerous cells.^122^ Carrot juice-derived CDs were non-covalently modified (electrostatic interaction) with PEI and Nile blue chloride (organic dye) to assemble a two-photon fluorescent nanoprobe for the detection of Cu^2+^ (turn-on–off) and S^2−^ (turn-off–on).^123^ The complexation/coordination strategy is also executed on CDs to attach functional species on their surface. For instance, when orange peel-derived CDs were modified with ethylenediamine tetraacetic acid (EDTA) *via* complexation, they showed higher sensitivity for the detection of Cr^6+^ compared to bare CDs due to the strong chelating ability of EDTA.^124^

## 5 Developments in the precursors and synthetic methods for animal/human biomass-derived CDs

**Fig. 4:**
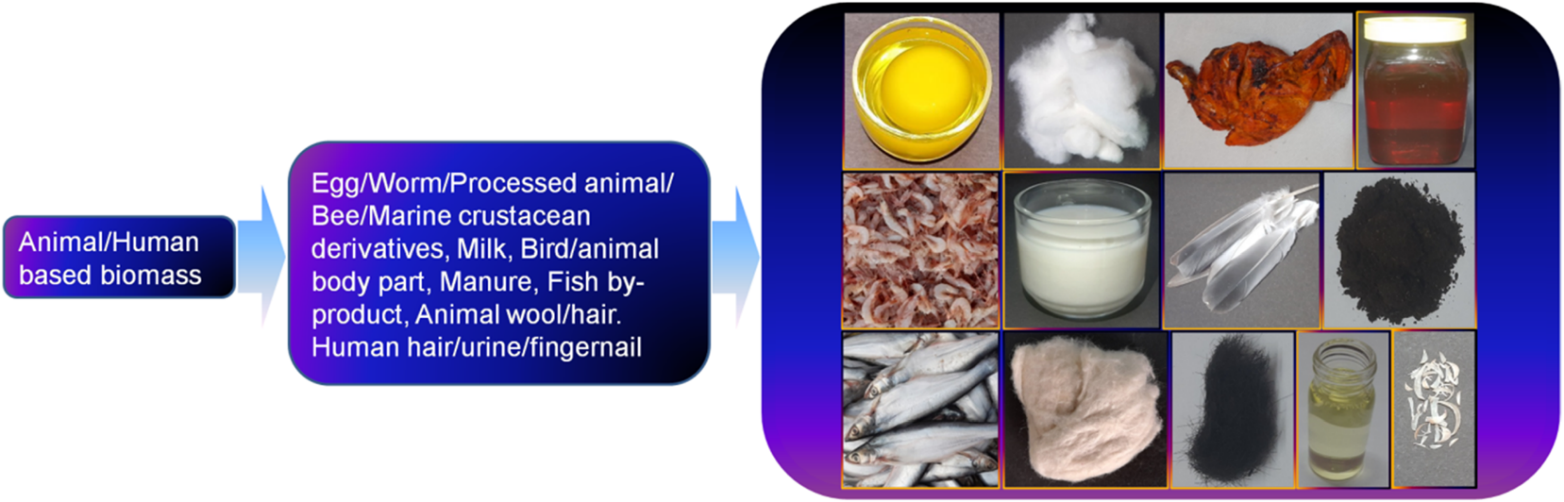
The representative photographs of the animal/human-based biomass applied for the synthesis of CDs. Top row (from left to right: egg white and yolk, SF, roasted chicken, and honey), middle row (from left to right: prawn, milk, pigeon feather, and cow manure), and bottom row (from left to right: fish, sheep wool, human hair, urine, and fingernails).

Besides plant-derived components as a natural resource, the use of animal- and human-based biomass has attracted tremendous interest over the past one decade for the synthesis of CDs and their doped counterparts. A representative illustration of the different animal/human-derived biomass used for the synthesis of CDs is shown in Fig. 4. These types of biomass are usually referred to as ‘green precursors’, which are sustainable, inexpensive, environmentally compatible, abundant, and renewable sources of carbon together with other elements. Moreover, the preparation of applied materials from these sustainable precursors is also a waste-to-wealth approach because many of them can be recognized as discarded biowaste. Although the synthesis of CDs from biomasses can be grouped into two broad categories, namely, top-down and bottom-up approach, the bottom-up protocol is widely implemented for this purpose. In this regard, the fusion of bio-derived organic precursor molecules under HT condition is one of the highly explored techniques; however, other methods such as pyrolysis, MW, alkaline hydrolysis, ultrasonication, MW-HT, thermal annealing, roasting (R)/grilling (G)/baking (B), acid carbonization, TD, autoclave TD (AT), and condensation are also adopted in the synthesis of biogenic CDs from various animal/human biomass (Table 1). Importantly, the bottom-up approach is advantageous compared to the top-down method for the production of CDs with a high yield, narrow size, heteroatom doping, good quality, and excellent property due to its simple and cost-effective operation. The AT calcination of grass at 180 °C and subsequent separation *via* centrifugation were probably the pioneering method for the synthesis of biogenic CDs.^125^ The authors proposed that these CDs are photoluminescent polymer nano-dots because of their crystalline structure with considerably smaller lattice spacing (0.20 nm) than that of graphitic carbon (0.34 nm). This pioneer report on the use of a natural source started a new trend of utilizing green biogenic resources for the synthesis of CDs. In this section, we summarize the synthesis and structural/compositional developments of CDs obtained from different animal and human biomass in chronological order using various synthetic approaches.

**Table 1:** A summary of the synthetic parameters, composition, size distribution, and QY of animal/human biomass-derived CDs^a^

Source	Synthetic method	Elemental composition (C, H, O, N, others)	Size distribution (nm)/average size or maximum population (nm)	QY (%)	Ref.
**Animal biomass-derived CDs**					
Egg white/yolk	Plasma-induced pyrolysis	[56.75, 6.03, 24.10, 13.12]^b^/[62.42, 8.74, 23.58, 5.26]^b^	3.39/2.15	6.0/8.0	126
Egg shell membrane	P (400 °C, 2 h); MW (5 min)	[13.78, —, 56.86, 1.60, 27.75 (Na)]^c^	2.0–12.0/5.0	14.0	127
Egg shell membrane	P (300 °C, 4 h, air)	[72.76, —, 10.58, 15.62, 1.04 (S)]^c^	1.0–5.0/3.35 ± 0.5	8.0	128
Egg white	HT (220 °C, 48 h)	[46.40, 9.15, 30.39, 13.94, 0.12 (S)]^b^	1.4–3.4/2.1	43, 61^e^	129
Egg white (albumin)	AH (1 M NaOH)	[C, O, N, S]^c^	—/3.2 ± 1.1	16.8	130
	MW (700 W, 5 min)	′′	—/13.4 ± 8.5	6.6	
	U (180 W, 5 h)	′′	—/4.1 ± 2.8	2.3	
Egg white	TD (200 °C, 4 h, air)	[C, O, N]^c^	—/3.3 ± 0.4	43.0	131
Egg white	MW (750 W, 25 min)	[46.66, —, 36.79, 15.91, 0.65 (S)]^c^	1.5–4.5/2.4 ± 0.6	—	132
Egg white	MW (1100 W, 3 min)	[56.68, 4.57, 28.13, 9.80, 0.82 (S)]^b^	—/2.98 ± 1.57	7.93	133
Pigeon egg white	P (300 °C, 3 h, air)	[68.83, —, 14.0, 9.44, 1.10 (S), 6.63 (Ot.)]^c^	2.2–4.2/3.3 ± 0.5	17.48	134
Pigeon egg yolk	′′	[78.51, —, 12.10, 5.83, 0.23 (S), 3.07 (Ot.)]^c^	2.2–4.2/3.2 ± 0.5	16.34	
Egg yolk oil	TD (260 °C, 1 h); D	[64.75, —, 10.16, 25.10 (Fe)]^d^	1.0–17.0/<10.0	5.01	135
Egg yolk	P (300 °C, 3 h, N_2_); HT (300 °C, 12 h)	[58.76, —, 39.65, 1.59]^c^	—/<10.0	35.0	136
Egg yolk	P (220 °C, 24 h, air)	[57.80, —, 29.20, 11.20]^c^	1.0–6.0/2.04	—	137
*Bombyx mori* silk	HT (190 °C, 3 h)	[—, —, —, 10.45]^c^	4.0–7.0/5.0	13.9	138
Cocoon silk + H_2_O_2_	HT (260 °C, 50 min)	[56.34, 5.63, 25.21, 12.82]^b^	3.3–10.3/7.42	24.0	139
		[53.81, —, 27.75, 18.43]^c^			
Silkworm chrysalis	MW-HT (210 °C, 45 min)	[71.32, —, 22.96, 5.72]^c^	13.0–26.0/19.0	46.0	140
*Bombyx mori* silk + CA	HT (200 °C, 3.5 h)	[C, O, N]^c^	4.0–6.0/5.6	61.1	141
Spider silk	HT (180 °C, 10 h)	[41.38, 8.65, 33.67, 16.30]^b^	1.5–5.5/3.65	21.5^e^	142
Silk fibroin	MW-HT (200 °C, 20 min)	[60.58, —, 21.72, 17.70]^c^	—/6.1 ± 0.7	15.0	143
Silk fibroin	HT (220 °C, 12 h)	[59.25, 4.86, 22.68, 13.21]^b^	—/7.5	38.0	144
Silk sericin	MW (800 W, 2.5 min)	[60.11, —, 35.78, 4.11]^c^	2.0–7.0/4.0	3.60	145
Silk sericin	P (150 °C, 2 h, air)	[64.28, —, 21.10, 14.62]^c^	4.0–6.5/5.3 ± 0.5	67.0	146
Silk fibroin + LMWC	P (230 °C, 3 h, N_2_)	—	2.0–4.5/3.0 ± 1.5	66.0	147
Barbeque beef	TA (5 h, Ar)	—	—	40.0	148
	RF (2.5 M HNO_3_); GC				
Hamburger	R (220 °C, 30 min); EE & D	[68.6, —, 17.34, 13.38, 0.56 (S), 0.12 (P)]^c^	2.0–10.0/5.1 ± 4.9	23.25	149
Pike eel fish	R (280 °C, 30 min); EE & D	[66.58, —, 17.50, 15.91]^c^	1.5–3.5/2.75	68.7	150
Pike eel fish	R (300 °C, 30 min); EE & D	[68.28, —, 15.33, 16.39]^c^	1.75–4.25/2.75	80.16	151
Lamb	B (250 °C, 30 min); EE & D	[78.31, —, 19.70, 1.77]^c^	0.8–3.8/2.0	10.0	152
Lamb	B (350 °C, 30 min); EE & D	[74.72, —, 17.11, 7.12]^c^	0.6–2.4/1.69	45.0	153
Lamb	B (280 °C, 45 min); EE & D	[58.26, —, 27.82, 9.61, 4.31 (S)]^c^	1.2–3.7/2.6	13.97	154
Duck	G (170 °C, 60 min); EE & GC	[70.48, —, 22.17, 6.25, 1.11 (S)]^c^	0.7–2.3/1.3	4.4	155
Duck	R (300 °C, 30 min); EE & D	[64.67, —, 22.12, 12.73]^c^	1.1–3.0/1.95 ± 0.41	38.05^e^	156
Chicken breast	R (230 °C, 30 min); EE & D	[69.87, —, 19.81, 9.91]^c^	0.6–2.8/1.7 ± 0.4	10.8	157
Chicken	R (300 °C, 30 min); EE & D	[67.61, —, 16.40, 15.36]^c^	0.5–3.5/2.1 ± 0.6	17.46	158
Chicken breast	R (200 °C & 300 °C, 30 min); EE & D	[71.69, —, 18.84, 9.07]^c^ & [67.15, —, 18.8, 13.29]^c^	1.0–4.5/2.8 & 0.3– 2.1/1.2	—	159
Chicken	R (250 °C, 30 min); EE & D	[75.70, —, —, 7.97]^c^	2.0–6.0/4.7	12.84	160
Mackerel fish	R (230 °C, 40 min); EE & D	[52.70, —, 22.30, 24.10]^c^	0.9–3.5/2.2	12.0	161
Mackerel fish	G (230 °C, 30 min); EE & GC	—	1.0–4.0/2.94 ± 0.03	—	162
Salmon fish	R (200 °C, 50 min); EE & HPLC	—	1.0–4.0/2.73 ± 0.48	—	163
Pork	R (280 °C, 30 min); EE & D	[C, O, N]^c^	3.5–8.5/5.93	17.11	164
Beef	R (280 °C, 30 min); EE & HPLC	[68.68, —, 15.98, 10.60]^c^	1.0–5.0/2.32 ± 0.71	—	165
Honey	HT (100 °C, 2 h)	[44.24, —, 45.95, 9.81]^c^	—/2.0	19.8	166
Rapeseed bee pollen	HT (180 °C, 48 h)	[52.30, 6.67, 32.52, 7.10]^b^	0.75–2.25/1.7	12.8	167
Camellia bee pollen	HT (180 °C, 24 h)	[44.69, 6.44, 40.82, 6.67]^b^	0.8–1.5/1.2	8.9	
Lotus bee pollen	′′	[41.30, 5.91, 46.70, 4.28]^b^	0.75–1.5/1.1	6.1	
Bee pollen	HT (200 °C, 24 h)	[C, O, N]^c^	1.5–2.3/2.01	7.7	168
Rapeseed bee pollen	HT (200 °C, 24 h)	[67.30, —, 25.10, 7.60]^c^	2.3–12.3/5.2	7.7	169
Honey	D (48 h)	[C, O]^c^	2.3–5.4/3.2 ± 1.5	1.6	170
	NaBH_4_ reduction			4.6	
Honey + Garlic extract + NH_3_	HT (200 °C, 6 h)	[C, O, N, S]^c^	4.0–13.0/8.29	4.19	171
Bee pollen (water)	HT (180 °C, 24 h)	[81.87, —, 13.78, 4.34]^c^	1.0–5.0/2.64	2.15^e^	172
Bee pollen (ethanol)	′′	[67.85, —, 19.64, 12.51]^c^	0.25–4.5/1.59	4.80^e^	
Shrimp egg	P (180 °C, 25 min)	[C, O, N]^c^	0.5–6.0/3.25	18.5	173
Prawn shell	RF (80 °C, 2 h); HT (200 °C, 8 h)	[C, O, N]^d^	2.0–8.0/4.0	9.0	174
Prawn shell	HT (180 °C, 12 h)	[68.50, —, 27.90, 3.60]^c^	1.0–5.0/3.0	—	175
Prawn shell + urea	HT (150 °C, 1 h)	—	7.0–15.0/—	5.84	176
Dried shrimp	HT (170 °C, 12 h)	[21.24, —, 48.65, 23.57, 6.22 (S), 0.32 (P)]^d^	1.0–13.0/6.0	54.0	177
Crab shell + GdCl_3_	MW (220 °C, 10 min)	[C, O, N, Gd]^c^	—/4.0 ± 0.7	19.84	178
Crab shell	U (20 kHz, 1 h)	[C, O, N]^c^	—/8.0	14.5	179
Crab shell	P (210 °C, 20 min)	[C, O, N]^c^	3.0–9.0/—	14.5	180
Crab shell	HT (180 °C, 12 h)	—	—/10.0	—	181
Shrimp shell	HT (180 °C, 15 h)	[C, O, N (14.40)]^c^	1.5–5.5/—	—	182
Shrimp shell	P (230 °C, 2 h, N_2_)	[C, O, N, S, P]^c^	3.0–5.0/—	20.0	183
Shrimp shell	HT (180 °C, 12 h)	[C, O, N]^c^	8.0–10.0/—	27.14	184
Crayfish shell	HT (200 °C, 8 h)	[77.19, —, 13.86, 8.59, 0.36 (S)]^c^	0.3–4.8/2.38 ± 0.14	50.2^e^	185
Crayfish shell	HT (200 °C, 6 h)	[66.82, —, 20.01, 12.70, 0.47 (S)]^c^	3.0–5.5/4.0	18.57	186
Crayfish shell	HT (180 °C, 8 h)	[63.0, —, 30.0, 7.0]^c^	2.0–5.5/3.8	10.68	187
Mussel seafood	HT (180 °C, 8 h)	[62.15, —, 25.21, 10.43]^c^	0.6–2.0/1.30 ± 0.25	15.2	188
Milk	HT (180 °C, 2 h)	[C, O, N]^c^	2.0–4.0/3.0	12.0	189
Milk	HT (180 °C, 8 h)	[62.50, 8.38, 24.45, 3.92, 0.75 (S)]^b^	4.6–5.3/5.0 ± 0.27	9.68	190
Milk + l-cysteine	′′	[46.83, 8.15, 29.37, 8.22, 7.43 (S)]^b^	3.78–3.98/4.0 ± 0.07	10.38	
Milk + urea	′′	[33.70, 7.14, 37.28, 20.88, 1.0 (S)]^b^	3.15–3.35/3.0 ± 0.07	15.39	
Cow milk	HT (180 °C, 12 h)	[92.15, —, 4.18, 3.66]^c^	2.0–5.0/—	9.6	191
Cow milk	HT (180 °C, 12 h)	—	1.0–5.0/—	—	192
Milk	MW (175 °C, 25 min)	[50.77, 6.47, 32.38, 10.38]^b^	1.5–3.0/2.3 ± 0.4	16.0	193
Milk	MW (1250 W, 2 min)	[73.30, —, 17.30, 9.30, 0.10 (P)]^c^	1.0–7.0/—	—	194
Milk	HT (180 °C, 4 h)	[C, O, N, P]^c^	—/10.0	10.0	195
Milk powder + FeCl_3_	HT (180 °C, 6 h)	[60.28, —, 32.76, 6.05, 0.91 (Fe)]^c^	2.0–4.5/2.9	8.73	196
Cow milk	HT (180 °C, 8 h)	—	1.0–3.0/2.0	—	197
Cow milk	HT (180 °C, 2 h)	[67.36, —, 22.73, 9.91]^c^	4.5–11.5/7.0	38.0	198
Cow milk	HT (180 °C, 4 h)	[61.12, —, 31.49, 6.06]^c^	1.0–2.0/1.6 ± 0.4	59.47	199
Milk powder + methionine	HT (220 °C, 3 h)	[C, O, N, S]^c^	—/3.4	32.7	200
Pasteurized milk	HT (170 °C, 12 h)	—	1.75–4.25/2.5 ± 1.0	5.7	201
Expired milk	SBCW (180 °C, 1.2 MPa, 2 h)	[70.11, —, 24.12, 5.77]^c^	1.1–2.7/2.0	8.64	202
Denatured milk	HT (120 °C, 3 h)	—	1.0–5.0/2.0	—	203
Denatured sour milk	HT (160 °C, 3 h)	[76.16, —, 17.52, 6.32]^c^	2.0–4.0/—	13.0	204
Goose feather	MW-HT (180 °C, 40 min)	[43.50, 5.57, 34.70, 14.40, 1.83 (S)]^b^	19.0–23.0/21.5	17.1	205
		[48.40, —, 33.30, 16.30, 1.90 (S)]^c^			
Pigeon feather	P (300 °C, 3 h, air)	[74.78, —, 12.76, 11.30, 1.16 (S)]^c^	2.8–4.3/3.8 ± 0.5	24.87	134
Chicken feather	HT (180 °C, 18 h)	[62.90, —, 35.80, 1.30]^d^	—/35.0	—	206
Carbonized hen feather + Zn salt	P (350 °C, 3.5 h, air); MW (900 W, 6 min)	[66.13, —, 26.16, 5.33, 2.37 (Zn)]^c^	2.0–5.5/4.09	10.34	207
Carbonized hen feather + Mg salt	′′	[53.0, —, 34.0, 9.0, 4.0 (Mg)]^c^	1.5–5.5/3.4	9.23	
Pig skin	HT (250 °C, 2 h)	[19.31 : 6.02 : 1.0 (C : O : N atomic ratio)]^c^	3.5–7.0/5.58 ± 0.21	24.1	208
Pig skin	P (300 °C, 2 h, N_2_); MW (400 W, 4 min)	[82.93, —, 13.52, 2.43, 1.12 (Ot.)]^c^	3.0–9.0/5.78	51.35	209
Pork	HT (200 °C, 10 h)	[C, O, N]^c^	2.1–4.9/3.5	17.3	210
Pig skin collagen	HT (240 °C, 3 h)	[61.85, —, 21.75, 15.66]^c^	0.8–1.8/1.25 ± 0.21	15.0	211
Pork rib bone	P (700 °C, 5 h); AC; HT (200 °C, 10 h)	[C, O, N, S, 5.35 (Ca)]^c^	—/4.2 ± 1.2	—	212
Pork liver	HT (180 °C, 5 h)	[64.06, —, 21.05, 14.88]^c^	1.3–4.5/3.2	11.74	213
Chicken drumstick	HT (190 °C, 5 h)	[C, O, N]^c^	2.0–9.0/5.0	32.86	214
Bovine bone	P (700 °C, 5 h); AC; HT (200 °C, 10 h)	[23.81, —, 53.28, 6.29, 1.54 (S), 15.09 (Ca)]^c^	—/6.6	8.4	215
Pork bone	′′	[28.59, —, 49.08, 7.19, 1.18 (S), 13.96 (Ca)]^c^	—/6.7	7.3	
Sheep bone	′′	[27.35, —, 51.57, 4.65, 1.73 (S), 14.70 (Ca)]^c^	—/9.5	8.0	
GGEC	HT (240 °C, 8 h)	[64.86, —, 27.90, 7.24]^c^	0.98–5.6/2.46	19.45	216
Chicken cartilage	HT (200 °C, 8 h)	[C, O, N]^c^	3.5–11.5/7.6	10.3^e^	217
Chicken bone	HT (180 °C, 4 h)	[75.34, —, 19.49, —]^c^	1.8–4.6/3.2 ± 0.2	—	218
Cow manure	P (300 °C, 3 h, air); RF (5 M HNO_3_, 72 h); AP	—	2.3–6.8/4.8	65	219
Cow manure	P (300 °C, 3 h, air); RF (5 M HNO_3_, 72 h); AP & 4-FPBA modification	[48.0, —, 39.10, 4.11, 3.45 (S), 5.42 (B)]^c^	2.0–6.0/4.2 ± 0.032	—	220
Pigeon manure	P (300 °C, 3 h, air)	[41.96, —, 29.71, 12.17, 1.26 (S), 14.9 (Ot.)]^c^	3.2–5.2/4.2 ± 0.5	33.5	134
Pigeon manure	HT (150 °C, 6 h)	[50.55, —, 37.81, 11.64]^c^	12.0–20.0/15.65	25.92	221
Fish scale (grass carp)	HT (200 °C, 24 h)	[60.35, —, 25.05, 14.60]^c^	1.0–4.0/—	17.08	222
Fish scale (grass carp)	HT (200 °C, 20 h)	[65.20, —, 21.90, 12.90]^c^	4.0–9.0/—	9.0	223
Fish scale (grass carp)	MW-HT (200 °C, 2 h)	[C, O, N, S]^c^	1.4–3.4/2.6 ± 0.8	19.92	224
Fish scale (crucian carp)	HT (200 °C, 20 h)	[70.0, —, 16.0, 14.0]^c^	5.0–10.0/—	6.9	225
Fish scale (*Lethrinus lentjan*)	HT (280 °C, 3 h)	—	3.0–15.0/—	—	226
Fish scale (*Ctenopharyngodon idella*)	Water extraction, 1 h	[64.87, —, 24.16, 4.54, 1.33 (S), 1.16 (P), 1.64 (Ca)]^c^	30.0–130.0/61.0 ± 3.6; 2.5–7.5/4.23 ± 0.13	15.6	227
Fish scale (*Dicentrarchus labrax*)	HT (200 °C, 24 h)	[66.0, —, 26.0, 8.0]^c^	—/10.0 (AFM)	6.0	228
Fish scale (*Dicentrarchus labrax*)	HT (200 °C, 24 h)	—	—/10.0	—	229
Fish scale (*Labeo rohita*)	HT (180 °C, 7 h)	—	4.0–5.0/—	—	230
Fish scale (silver carp)	HT (200 °C, 4 h)	[65.80, —, 21.10, 13.10]^c^	1.7–6.5/4.02 ± 0.89	6.04	231
	MW (400 W, 1.5 min)	[59.40, —, 24.10, 16.50]^c^	2.4–7.2/4.23 ± 1.03	5.10	
Shark cartilage (chondroitin sulphate)	HT (240 °C, 3 h)	[C, O, S, Na]^d^	19.6–60.0/50.0	20.46	232
Carp roe	HT (200 °C, 12 h)	[66.0, —, 20.02, 13.98]^c^	6.04–9.42/7.60	13.4^e^	233
Fish scale collagen peptides	HT (190 °C, 2 h)	[65.10, —, 21.50, 13.40]^c^	1.15–3.55/2.27 ± 0.48	9.29	234
	MW (400 W, 3 min)	[62.50, —, 21.10, 16.40]^c^	5.75–9.75/7.58 ± 0.88	4.86	
Sheep wool	P (300 °C, 2 h); MW (700 W, 2 min)	[4.74 : 1.72 : 1.0 (C : O : N atomic ratio)]^c^	2.7–9.3/6.05 ± 1.67	22.5	235
Sheep wool	MW-HT (200 °C, 1 h)	[C, O, N, S]^c^	1.5–4.5/2.8	16.3^e^	236
Sheep wool	HT (240 °C, 6 h)	[62.54, —, 23.05, 13.66, 0.75 (S)]^c^	4.0–8.0/5.9	25.6	237
Pig hair	′′	—	—	20.1	
Wool keratin	HT (200 °C, 10 h)	[C, O, 14.05 (N), S]^c^	2.0–6.0/—	8.0^e^	238

**Human biomass-derived CDs**					
Hair fiber	AC (40 °C, 24 h)	[61.22, —, 31.21, 5.43, 2.14 (S)]^c^	4.0–10.0/7.5	11.1	239
	AC (100 °C, 24 h)	[59.73, —, 30.87, 5.37, 4.03 (S)]^c^	2.0–7.0/4.2	4.02	
	AC (140 °C, 24 h)	[60.56, —, 28.72, 5.64, 5.08 (S)]^c^	2.0–5.0/3.1	5.38	
Hair	P (300 °C, 2 h, N_2_)	[74.12, 6.46, 11.59, —]^b^	—/2.3	17.3	240
Hair	AT (200 °C, 24 h)	[C, O, N]^c^	2.0–8.0/4.56	10.75	241
Hair	P (300 °C, 2 h, N_2_); MW (400 W, 4 min)	[74.66, —, 14.75, 9.66, 0.93 (Ot.)]^c^	2.0–7.0/3.57	86.06	209
Hair	AT (180 °C, 24 h)	[76.60, —, 13.80, 8.91]^c^	7.0–13.0/11.0	38.0	242
	MW (1100 W, 5 min)	[84.98, —, 13.81, 1.21]^c^	65.0–90.0/78.0	17.0	
Hair	P (300 °C, 1 h, air)	[91.72, —, 3.22, 5.06]^c^	1.0–2.6/1.76	38.0	243
Urine (unmodified diet)	TD (200 °C, 12 h)	—	10.0–40.0/20.6 ± 8.4	5.3	244
Urine (vitamin C supplemented)	′′	′′	2.0–28.0/11.4 ± 6.6	4.3	
Urine (asparagus rich diet)	′′	′′	10.0–70.0/38.8 ± 20.6	2.7	
Urine + citron juice	HT (180 °C, 7 h)	[45.12, —, 38.30, 16.58]^d^	2.5–5.5/4.0	34.5^e^	245
Urine	CN & GC	[67.80, —, 27.57, 3.70, 0.65 (S), 0.28 (Fe)]^c^	1.0–5.0/2.5	4.8	246
	HT (200 °C, 8 h), GC	[64.29, —, 27.20, 5.48, 2.79 (S), 0.23 (Fe)]^c^	2.0–9.0/5.5	17.8	
Fingernail + H_2_SO_4_	MW (400 W, 2 min)	[57.80, —, 34.80, 5.90, 1.50 (S)]^c^	1.8–3.0/2.2	42.8	247
Fingernail	P (200 °C, 3 h, air)	[55.80, —, 23.50, 19.20, 1.50 (S)]^c^	2.0–4.5/3.5	81.4	248
Fingernail	HT (200 °C, 3 h)	—	1.96–4.15/3.1	—	249

a P: pyrolysis, AH: alkaline hydrolysis, U: ultrasonication, D: dialysis, TA: thermal annealing, RF: reflux, GC: gel column, EE: ethanol extraction, HPLC: high-pressure liquid chromatography, SBCW: subcritical water treatment, AC: acid carbonization, AP: amine passivation, CN: condensation, Ot.: other elements, and AFM: atomic force microscopy.

b From elemental analysis.

c From XPS analysis.

d From TEM/SEM EDX analysis.

e Absolute quantum yield.

### 5.1 Animal-derived precursors

The components of animal derivatives include an abundance of proteins, fats, carbohydrates, amino acids, vitamins, and minerals. These constituents hold potential to serve as viable biosources for the synthesis of both CDs and their doped analogues.

#### 5.1.1 Animal egg derivatives as a carbon source

Chicken eggs contain a lot of proteins, carbohydrates, and fats together with heteroatoms, and therefore can be successfully used in the preparation of CDs *via* the bottom-up approach. Edible chicken egg (white and yolk) is probably the first animal-derived natural source employed for the synthesis of amphiphilic CDs *via* plasma-induced pyrolysis.^126^ Subsequently, different chicken egg-based products such as egg shell membrane,^127,128^ egg white,^129–133^ pigeon egg white/yolk,^134^ egg yolk oil,^135^ and egg yolk^136,137^ have been used for the preparation of doped CDs *via* various synthetic methods (Table 1). Among them, the treatment of egg white under HT^129^ or TD^131^ condition yielded N-CDs with a narrow size and excellent relative QY (43.0%). Recently, the MW-assisted synthesis of N,S-CDs from egg white was reported, which showed a low QY (7.93%).^133^ Also, egg yolk-derived N-CDs obtained *via* pyrolysis followed by HT treatment showed a high QY (35.0%), which was maintained even after 6 month (32.0%).^136^ Recently, egg yolk was also used to synthesize N-CDs *via* the pyrolysis method with a small average size of 2.04 nm and fairly high N content of 11.2 atomic%.^137^ Therefore, it can be concluded that the HT or TD/pyrolysis approach employing chicken egg white/yolk may be feasible to produce narrow-sized heteroatom-doped CDs with excellent QY.

#### 5.1.2 Worm derivatives as a carbon source

Silk fibroin (SF), extracted from *Bombyx mori* silkworm/cocoon, is a nontoxic natural composite of proteins (fibroin, ∼75 wt%) and sericin (∼25 wt%). The N content in SF is as high as 18.0%, and therefore it can potentially be applied for the synthesis of doped CDs. However, despite its high N content, the earlier reports of SF-derived N-CDs synthesized *via* the HT method showed a low QY (Table 1).^138,139^ Subsequently, N-CDs directly derived from silkworm chrysalis *via* the MW-HT method showed a very high QY (46.0%) although they had a relatively large average diameter and small N content (Table 1).^140^ Interestingly, when SF was HT treated together with an equal amount of CA, narrow-sized N-CDs with a relatively high relative QY (61.1%) were obtained compared to the previous reports (Table 1).^141^ The high QY of N-CDs in this study is attributed to the surface passivation and formation of a pyrrolic structure *via* the effective N heteroatom doping in the carbon nanosheet-type core domain. Subsequent reports of N-CDs derived from spider silk,^142^ SF,^143,144^ and silk sericin^145,146^ showed appreciable QYs (Table 1). The high QY of 67.0% for the N-CDs (derived from silk sericin) prepared at a low pyrolysis temperature (150 °C) was ascribed to the formation of a carbon core together with O, N-based functional groups on its surface. Consequently, the created surface defects trapped excitons followed by radiative recombination to furnish a high QY.^146^ In another recent report, Horo *et al.*^147^ synthesized amine-functionalized CDs *via* the pyrolysis of SF (N source) and low molecular weight chitosan (LMWC, C source and functionalizing precursor) blends under an N_2_ atmosphere at 230 °C. They achieved a fairly high QY of 66.0% for the CDs obtained by LMWC : SF blends in a ratio of 1 : 1.5. The above-mentioned results clearly indicate that SF together with CA or LMWC and silk sericin may be a good biosource under the HT or pyrolysis condition to produce doped CDs with a narrow size distribution and high QY.

#### 5.1.3 Processed animal products as a carbon source

The isolation of CDs from processed animal derivatives began with char from overcooked barbeque beef.^148^ Subsequently, CDs were extracted from roasted hamburger,^149^ grilled/roasted pike eel,^150,151^ baked lamb,^152–154^ roasted duck,^155,156^ roasted chicken/chicken breast,^157–160^ roasted mackerel fish,^161,162^ roasted salmon fish,^163^ roasted pork,^164^ and roasted beef.^165^ Among them, the excellent QYs of the roasted pike eel-derived N-CDs (68.7% and 80.16%) were remarkable, which were attributed to the abundant N-doping (15.91 and 16.39 atomic%, respectively) and surface-passivated amine-linkage or stable excited states.^150,151^ It was observed that the roasting/baking temperature^149,151,153,156,158,159,164^ or baking time^154^ had a profound impact on the structure–property relationship of the derived CDs. For example, N-CDs derived from roasted duck showed an increase in absolute QY of 10.53% (200 °C), 13.80% (250 °C), and 38.05% (300 °C) with an increase in the roasting temperature, which was ascribed to the increase in N contents in the N-CDs of 7.18%, 8.70%, and 12.73%, respectively. Moreover, the average particle size of N-CDs, *i.e.*, 2.59 ± 0.57 nm, 2.00 ± 0.53 nm, and 1.95 ± 0.41 nm, respectively, decreased and gradually became uniform with an increase in the roasting temperature.^156^ Alternatively, the QY and average size of the baked lamb-extracted N,S-CDs (280 °C) at different baking times, *i.e.*, 15/30/45 min, were found to be 2.93/10.89/13.97% and 4.1/3.7/2.6 nm, respectively. The authors suggested that the ingredients in the lamb initially produced large nanostructures, which subsequently turned into smaller CDs with an increase in the baking time.^154^

#### 5.1.4 Bee derivatives as a carbon source

Honey, which mainly contains glucose, fructose, carbohydrates, proteins, amino acids, vitamins, enzymes, polyphenols, and minerals, can be obtained directly from bee hives. Earlier reports demonstrated the synthesis of N-CDs from honey^166^ and bee pollen^167–169^ using the HT method. The presence of nanoscale CDs (2.3–5.4 nm) in natural honey and improvement in QY after NaBH_4_ reduction (from 1.6% to 4.6%) *via* surface modification were further confirmed in a later report; however, the QY was much lower than the earlier report (Table 1).^170^ N,S-CDs prepared from the HT treatment of natural honey (C source), garlic (S source), and ammonia (N source) also yielded a similar QY as that of NaBH_4_-treated CDs (Table 1).^171^ Bee pollen biomass was further used to prepare N-CDs *via* the HT method. The bee pollen extract in water resulted in WBCDs (absolute QY: 2.15% and average size: ∼2.64 nm), while the pollen extract in ethanol produced EBCDs (absolute QY: 4.80% and average size: ∼1.59 nm).^172^ However, despite the high N content in EBCDs compared to WBCDs and previously reported bee pollen-derived N-CDs,^167^ the QY did not improve (Table 1), which signifies that high N doping alone may not be enough to achieve a high QY.

#### 5.1.5 Marine crustacean derivatives as a carbon source

**Fig. 5:**
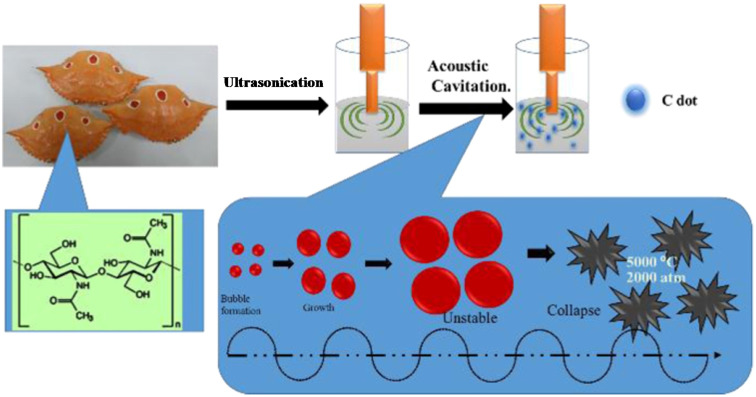
A schematic illustration of N-CDs obtained from crab shells by a sonochemical treatment. Reprinted from ref. 179, Copyright (2018), with permission from Elsevier.

Marine crustaceans such as shrimp, crab, prawn, and crayfish are highly consumed seafood worldwide, and also recognized as natural sources for the synthesis of animal biomass-derived CDs. Heteroatom-doped CDs were successfully synthesized from shrimp egg,^173^ prawn shells,^174–176^ dried shrimp,^177^ crab shells,^178–181^ shrimp shells,^182–184^ crayfish shells,^185–187^ and mussel seafood^188^ using different synthetic protocols (Table 1). The applicability of the ultrasonication method in the synthesis of N-CDs from crab shells is shown in Fig. 5.^179^ Recently, the N-CDs synthesized from shrimp shell^184^ using the HT method possessed a size in the range of 8–10 nm and improved QY (27.14%) compared to the previously reported shrimp shell-derived doped CDs using the pyrolysis method (20.0%);^183^ however, it was much lower than that of the dried shrimp-derived N-CDs using the HT method (54.0%),^177^ justifying that dried shrimp is a good animal derivative and HT is an excellent synthetic process. The elemental analysis (EA) of N-CDs derived from dried shrimp by energy dispersive X-ray (EDX) spectroscopy showed the presence of 23.57 wt% of N atoms together with other elemental (S: 6.22 wt% and P: 0.32 wt%) peaks, and therefore the fairly high QY is attributed to the synergistic role of other elements together with the high N content for excellent fluorescence properties.^177^ A high absolute QY of 50.2%^185^ and good relative QY of 18.57%^186^ for N,S-CDs recently derived from crayfish shell using the HT method were also remarkable.

#### 5.1.6 Animal milk as a carbon source

Milk is another common animal derivative, which contains many nutrients such as proteins, fat, lipids, carbohydrates, lactose, and minerals. HT or MW-assisted CDs/doped analogues were successfully synthesized using milk in earlier reports.^189–195^ Some recent reports also evidenced the synthesis of doped CDs using cow milk/milk powder *via* the HT method.^196–200^ For example, Zhang *et al.*^199^ obtained N-CDs with a narrow size distribution (1.0–2.0 nm) and excellent QY (59.47%) using cow milk and the HT method. Another recent report also showed that HT-synthesized N,S-CDs from a mixture of milk powder and methionine (S source) showed a good QY (32.7%).^200^ Alternatively, pasteurized/expired/denatured milk-derived CDs resulted in relatively low QYs in the range of 5.7–13.0% (Table 1).^201–204^ These results clearly reflect that the HT treatment of pure milk is a good choice to obtain narrow-sized N-CDs with satisfactory QYs.

#### 5.1.7 Birds and animal body parts as a carbon source

**Fig. 6:**
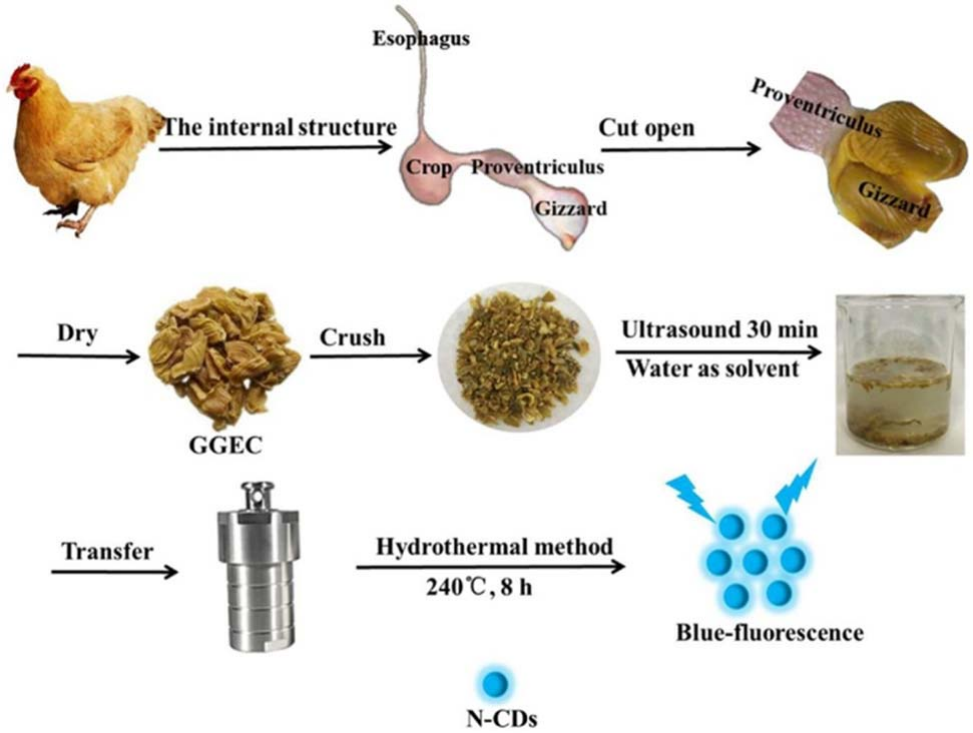
A systematic diagram of N-CDs derived from GGEC by the HT method. Reprinted from ref. 216, Copyright (2021), with permission from Elsevier.

Birds and animal body parts such as goose/pigeon/chicken/hen feathers,^134,205,206,207^ pig skin,^208,209^ pork,^210^ pig skin collagen,^211^ pork rib bones,^212^ pork liver,^213^ chicken drumstick,^214^ animal bones,^215^ inner wall of chicken stomach (Galli Gigerii Endothelium Corneum, GGEC),^216^ chicken cartilage,^217^ and chicken bones^218^ are consumed for the preparation of heteroatom-containing CDs using various synthetic methods (Table 1). Noticeably, the effective self-doping of Ca element can be seen from animal bones-derived CDs; however, QYs are not as high as that in other animal body part-synthesized CDs (Table 1).^212,215^ For example, pig skin-derived N-CDs prepared by pyrolysis followed by MW irradiation possessed a high QY (51.35%).^209^ The synthetic steps involved in the preparation of N-CDs from GGEC *via* HT treatment at 240 °C for 8 h are shown in Fig. 6.^216^ The N-CDs in this report showed a small average particle size (∼2.46 nm) and good QY (19.45%) due to the effective graphitization of the core and increased N content in the core graphene, which resulted in more PL contribution from the core state rather than the molecular state. X-ray photoelectron spectroscopy (XPS) analysis also validated the low amino N-content compared to the graphene N-content in the synthesized N-CDs. Recently, hen feathers were applied as an animal source together with Zn/Mg salt for the synthesis of Zn/Mg-doped CDs. Interestingly, the Zn/Mg-doped CDs exhibited a smaller size (4.09/3.4 nm) and better QY (10.34/9.23%) compared to the undoped CDs (5.43 nm, 8.15%).^207^

#### 5.1.8 Manure as a carbon source

As animal/bird biowaste, cow/pigeon manure is another source of carbon due to its high cellulose content. An earlier report showed that amine-passivated CDs (QY: 65%) can be obtained from the calcination of cow manure at 300 °C in air, followed by surface modification.^219^ The same research group further extended the surface functionalization of amine-passivated CDs *via* 4-formylphenylboronic acid (4-FPBA) covalent bonding, which resulted in PBA-CDs with N, S, and B hetero-elements.^220^ Heteroatom-doped CDs were also synthesized from pigeon manure^134,221^ with a QY as high as 33.5% *via* the simple pyrolysis of manure at 300 °C in air for 3 h.^134^

#### 5.1.9 Fish by-products as a carbon source

**Fig. 7:**
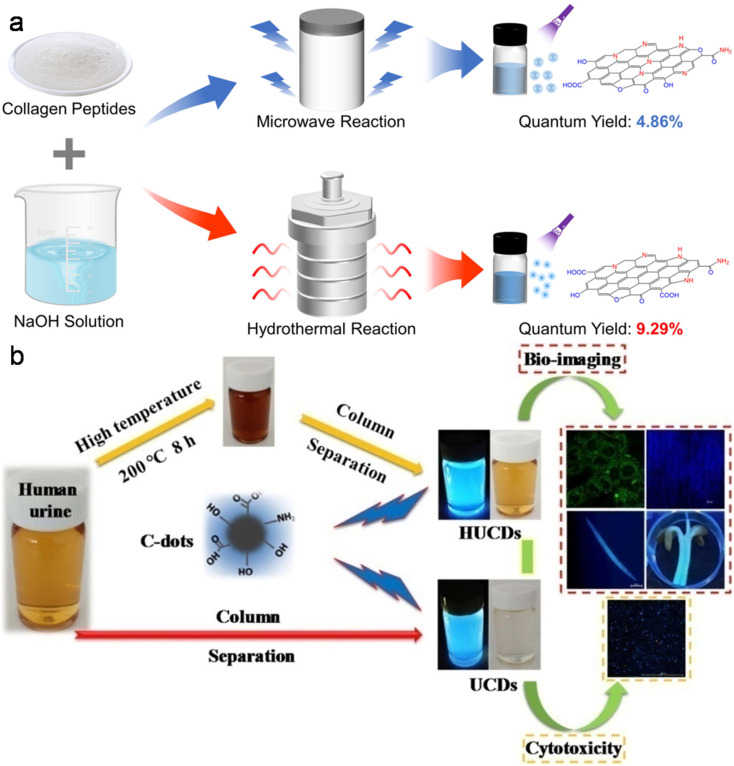
(a) MW and HT methods involved in the synthesis of N-CDs from fish scale collagen peptides. Reproduced/adapted from ref. 234 with permission from The Royal Society of Chemistry, 2023. (b) A schematic of the synthesis of UCDs/HUCDs from human urine and their applicability in bioimaging. Reprinted from ref. 246, Copyright (2019), with permission from Elsevier.

Collagen protein-, fat-, and vitamin-rich fish scale can be employed as another waste bio-precursor for the synthesis of doped CDs. Fish scales of grass carp,^222–224^ crucian carp,^225^*Lethrinus lentjan*,^226^*Ctenopharyngodon idella*,^227^*Dicentrarchus labrax*,^228,229^*Labeo rohita*,^230^ and silver carp^231^ have been employed in the synthesis of heteroatom-containing CDs using the HT or MW synthetic protocol (Table 1). Besides fish scale, other fish byproducts such as shark cartilage,^232^ carp roe,^233^ and fish scale collagen peptides^234^ have also been employed to produce doped CDs. Table 1 clearly indicates that the QY of fish scale-derived doped CDs can reach up to 19.92%.^224^ Moreover, the QY of shark cartilage-derived doped CDs (20.46%) is also appreciable.^232^ A recent report on the preparation of fish scale collagen peptide-derived N-CDs under MW and HT conditions (Fig. 7a) showed a significant difference in their size, structure, and surface composition. The QY of the HT-derived N-CDs (9.29%) was found to be higher than that of the MW-synthesized N-CDs (4.86%) (Fig. 7a), which is ascribed to the higher carbonization and smaller size of N-CDs.^234^

#### 5.1.10 Animal wool and hair as a carbon source

Sheep wool^235–237^ and pig hair^237^ have also been used for the synthesis of doped CDs *via* different synthetic processes (Table 1), with the N,S-CDs obtained from the HT treatment of sheep wool showing a high QY (25.6%).^237^ Wool keratin (α-keratin), which contains cysteine (amino acid) as an important constituent, was also utilized by Song *et al.*^238^ to synthesize N,S-CDs *via* the HT method. The size of N,S-CDs was found to be in the range of 2–6 nm along with an N content as high as 14.05%. However, the absolute QY measured at an excitation wavelength of 365 nm was fairly low (8.0%) compared to the animal wool/hair-derived doped CDs (Table 1).

### 5.2 Human-derived precursors

Besides animal biomass, various nontoxic human-derived raw/waste sources such as hair, urine, and fingernails have also been applied to produce fluorescent CDs and their doped counterparts.

#### 5.2.1 Human hair as a carbon source

Keratin is the main component (∼99.0%) of hair, which is rich in C, O, and N elements. In the earlier reports on doped CDs derived from human hair,^209,239–241^ it was observed that narrow-sized N-CDs (2.0–6.0 nm) with a QY as high as 86.06% were obtained from the pyrolysis of hair followed by MW irradiation.^209^ Subsequently, Singh *et al.*^242^ produced N-CDs from hair under AT (OCDs) and MW (MCDs) conditions. Interestingly, OCDs obtained under the conventional AT carbonization showed a much higher QY (38.0%) compared to the MCDs (17.0%), surpassing the QY of previously reported N-CDs (10.75%) using a similar synthetic protocol.^241^ The average size of OCDs was considerably smaller (∼11.0 nm) and possessed a higher N content (8.91%) compared to MCDs (average size: ∼78.0 nm and N content: 1.21%), which indicates that the surface defects and surface constituents play an important role in the emission properties.^242^ A recent report on human hair-derived N-CDs synthesized under pyrolysis condition (300 °C, 1 h, air) also showed a high QY of 38.0% together with a narrow size distribution (1.0–2.6 nm).^243^

#### 5.2.2 Human urine as a carbon source

The liquid portion of sewage waste is majorly contributed by urine, which contains a significant proportion of urea and some amount of salts (Na^+^, K^+^, and Cl^−^) and heterocyclic compounds (uric acid, creatinine, *etc.*). Therefore human urine may be upcycled as a green and waste source to produce doped CDs. Unmodified (UPDs) and diet-modified (CPDs and APDs) pee dots were synthesized in an earlier report with different sizes and QYs using human urine (Table 1).^244^ Subsequently, N-CDs derived from a mixture of human urine and citron fruit extract *via* the HT method showed a significantly improved absolute QY (34.5%).^245^ Human urine was further explored to obtain doped CDs *via* a Sephadex G-25 gel separation protocol (UCDs) and HT method followed by column chromatography (HUCDs) (Fig. 7b).^246^ Interestingly, the QY of HUCDs (17.8%) was fairly high compared to UCDs (4.8%) due to the high content of N (5.48%) together with S element (2.79%), leading to more surface-trapped excitons under the excitation process.

#### 5.2.3 Human fingernail as a carbon source

Human fingernails are a natural, low-cost, and non-toxic precursor, which primarily consist of α-helical fibrous protein (keratin). The MW dielectric heating of a mixture of fingernails and conc. H_2_SO_4_ resulted in narrow-sized N,S-CDs (1.8–3.0 nm) with a high QY (42.8%).^247^ Subsequently, N,S-CDs produced by the same group *via* the simple pyrolysis of fingernails showed a much higher QY (81.4%).^248^ The authors proposed that the existence of N and S elements in the form of cysteine-like RHN–C–C–SH structures was responsible for the high QYs of N,S-CDs.^247,248^ Afterwards, fingernails were again used without hazardous H_2_SO_4_ to obtain small-sized CDs (∼3.1 nm) under HT condition.^249^

Based on the overall summary discussed above, it can be concluded that HT treatment is the most versatile approach to produce animal/human biomass-derived CDs with a high QY, narrow size, and effective heteroatom doping; however, the pyrolysis, MW, and MW-HT methods are also applicable to various animal/human biomass precursors. The starting precursor and initial composition also play an important role in achieving good-quality CDs. Significant improvements in the QYs can also be observed in some of animal/human-derived precursors by tuning the experimental conditions.

### 5.3 Formation mechanism of CDs derived from animal/human biomass

Some research groups discussed the formation mechanism of animal/human-derived CDs in their reports. For instance, the formation mechanism of N-CDs from egg white begins with the hydrolysis of egg protein into peptides and amino acids during the HT process. Subsequently, partial polymerization, followed by the carbonization of amino acids results in the generation of a carbon core coated with oligomers. With the progression of the reaction, the oligomers are gradually eliminated from the core surface to form N-CDs with –OH and –COOH functionalities.^129^ Subsequently, a similar formation mechanism was proposed for doped CDs derived from egg albumin/egg white using the alkaline hydrolysis/MW synthetic method.^130,133^

He *et al.*^139^ presented the combined effect of the top-down/bottom-up strategy in the formation of N-CDs from cocoon silk. The ˙OH radicals generated from H_2_O_2_ under HT condition cleave silk into small microrods. As the reaction proceeds, the fragments from the microrods get hydrolyzed into small molecules. Subsequently, the dehydration and polymerization of these molecules nucleate nanospheres for the formation of N-CDs *via* intermolecular dehydration/polymerization.

The MW-assisted transformation of silk sericin into N-CDs was studied through gas chromatography-mass spectrometry, which exhibited the release of various gases (CO_2_, NH_3_, N_*x*_O_*y*_, C_*x*_H_*y*_O_*z*_, H_2_O, *etc.*) during operational condition. The high/instant temperature and generated OH^−^ cleave sericin into amino acids and polypeptides. These active components get aggregated and carbonized in subsequent steps to form a graphitized carbon core attached with heteroatom-containing functional groups.^145^ The N-CDs produced from silk sericin under pyrolysis condition underwent dehydration, depolymerisation, carbonization, and self-passivation to form a carbon core decorated with functional groups. It was also observed that the graphitization of the core carbon (*I*_D_/*I*_G_: 0.95/0.69/0.63/0.51) and average size of N-CDs (5.3/6.7/8.4/9.6 nm) increased with an increase in the pyrolysis temperature (150/200/250/300 °C).^146^

**Fig. 8:**
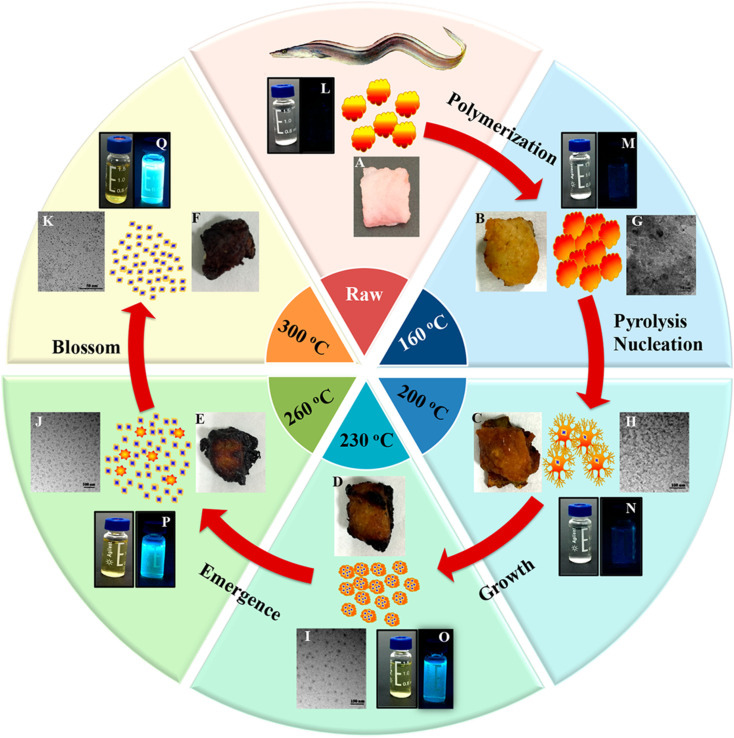
A stepwise illustration of the growth and formation mechanism of pike eel-derived N-CDs (red arrow). (A–F) The digital pictures of fish mass obtained at different roasting temperatures. (G–K) TEM images of corresponding fluorescent materials isolated at various stages. (L–Q) The digital photographs of aqueous soluble CDs under day per UV (365 nm) light. Reprinted (adapted) with permission from ref. 151, Copyright (2018), the American Chemical Society.

Bi *et al.*^151^ discussed the formation mechanism of N-CDs extracted from pike eel at different roasting temperatures, indicating the intensified polymerization/carbonization of fish ingredients (carbohydrates, proteins, and lipids) at a higher roasting temperature. The light pink fish flesh (Fig. 8A) turned a golden colour (Fig. 8B) at 160 °C, which was composed of irregular microstructures (Fig. 8G) with weak fluorescence (Fig. 8M) due to its insufficient combustion. When the roasting temperature was increased to 200 °C, the flesh showed a brown/curled appearance (Fig. 8C) with the evolution of a few carbon nanoparticles in the microstructure (Fig. 8H) and weak fluorescence (Fig. 8N), indicating the disintegration of the large biopolymer clusters. The formation of char on the surface of the flesh (Fig. 8D) and shrinkage of the clusters (Fig. 8I) occurred at a roasting temperature of 230 °C due to the extension of carbonization, which also showed stronger fluorescence (Fig. 8O). A further increase in the roasting temperature (260 °C) resulted in more charred product on the flesh surface (Fig. 8E), possessing a large number of CDs (Fig. 8J) and intensified blue fluorescence (Fig. 8P). The disintegration of the biopolymer clusters was significant at this stage, which was further extended at 300 °C. Consequently, the flesh surface turned into charred mass (Fig. 8F) having small-sized CDs (Fig. 8K, 1.75–4.25 nm) with strong cyan fluorescence (Fig. 8Q). A similar observation/explanation of escalated carbonization at a higher processing temperature was also presented for N-CDs derived from duck^156^ and chicken.^158^

The growth mechanism of N-CDs derived from human hair using the AT/MW method starts with the cutting/unzipping of the tertiary structures present in hair to generate a significant amount of small hydrocarbon. Subsequently, the C–C bonds are disintegrated with the evolution of nitrogen (depolymerisation), followed by cross-linking to form N-CDs.^242^

## 6 Physiochemical properties of animal/human biomass-derived CDs

### 6.1 Structural characteristic and chemical composition

**Fig. 9:**
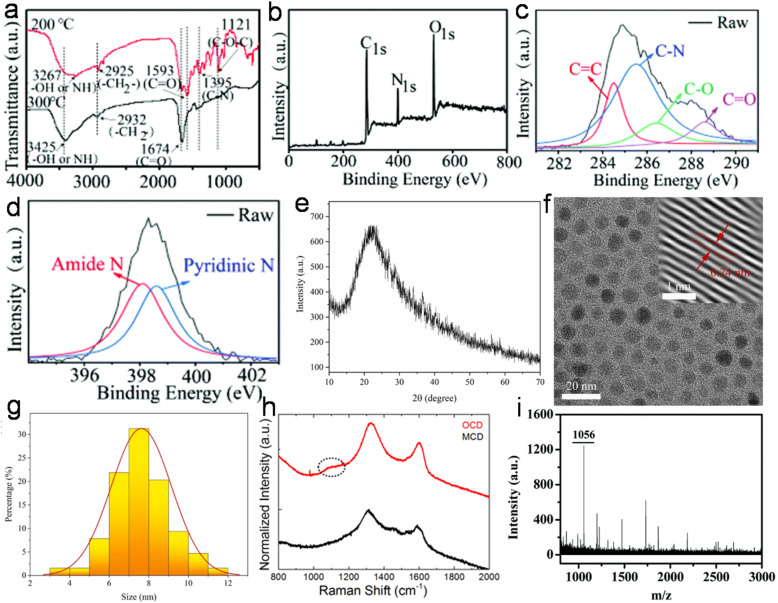
(a) The FTIR spectra of N-CDs extracted at 200/300 °C. (b) The XPS survey spectrum, (c) C 1s fine spectrum, and (d) N 1s fine spectrum of N-CDs obtained at 300 °C from chicken breast as animal biomass. Reproduced/adapted from ref. 159 with permission from The Royal Society of Chemistry, 2020. (e) The PXRD profile, (f) TEM/HRTEM (inset) image, and (g) size distribution of N-CDs derived from chicken cartilage. Reprinted from ref. 217, Copyright (2022), with permission from Elsevier. (h) The Raman spectra of N-CDs (OCD and MCD) synthesized from human hair. Reprinted from ref. 242, Copyright (2020), with permission from Elsevier. (i) The MALDI-TOF-MS spectrum of roasted salmon fish-derived CDs. Reproduced/adapted from ref. 163 with permission from The Royal Society of Chemistry, 2019.

**Fig. 10:**
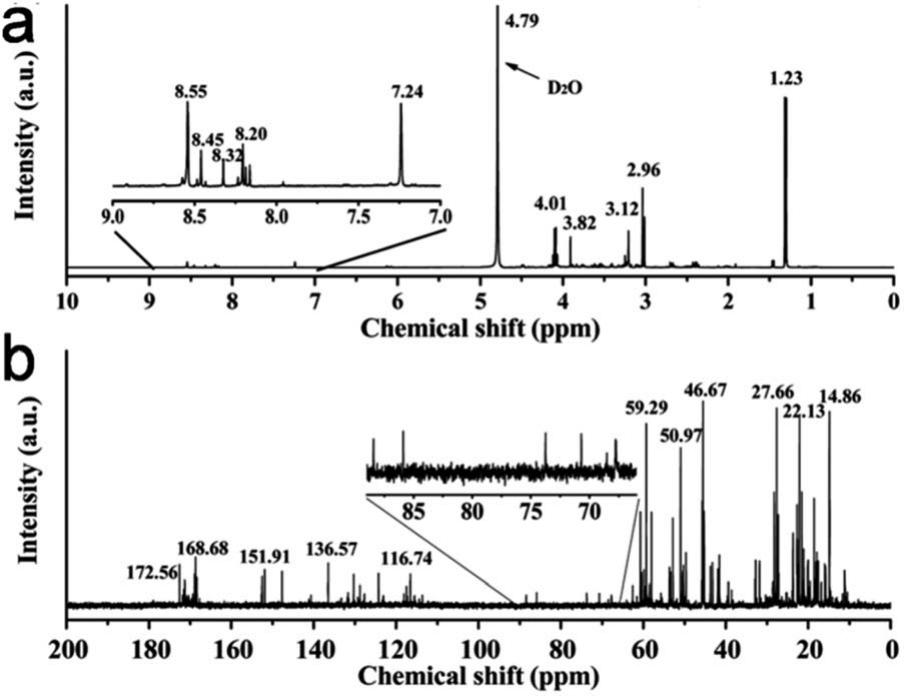
(a) The ^1^H NMR and (b) ^13^C NMR spectra of CDs isolated from roasted salmon fish. Reproduced/adapted from ref. 163 with permission from The Royal Society of Chemistry, 2019.

Structurally, most animal/human biomass-derived CDs are composed of a carbogenic core (predominantly sp^2^-hybridized carbon) and outer shell comprised of O- and/or N-containing functional groups attached to its surface. C, O, and N are found to be very common elements in these CDs; however, the successful doping of other elements (S, P, Fe, Zn, Mg, *etc.*) has also be reported, as shown in Table 1. There are numerous reports showing that the incorporation of heteroatoms significantly modifies the composition of CDs and effectively improves their QY (Table 1). The surface functionalities of the shell not only provide good aqueous solubility/dispersibility, but also tune the optical properties of animal/human biomass-derived CDs. Generally, surface zeta potential analysis is used to determine the strength of the electrostatic repulsion amongst CDs due to the presence of abundant surface charge. A large negative zeta potential value suggests strong electrostatic repulsion among the negatively charged functional groups and better stability of animal/human biomass-derived CDs in aqueous solution.^128,129,131,132,140–142,144,185,189,191,197,203,207,208,215^ Besides a negative zeta potential, animal/human-derived CDs with positive zeta potentials have also been reported.^137,198,230,236,243^ The functional groups and elemental compositions of animal/human biomass-derived CDs are often analyzed by Fourier transform infrared (FTIR) spectroscopy and XPS. Fig. 9a–d show the representative FTIR and XPS results of chicken breast-derived N-CDs, respectively.^159^ The N-CDs obtained at a roasting temperature of 200/300 °C presented broad absorption peaks at ∼3267/3425 cm^−1^ due to the stretching modes of the O–H or N–H bonds. The peaks at ∼2925/2932 cm^−1^ were assigned to the –CH_2_ stretching vibrations. The typical absorption peaks at ∼1593/1674 cm^−1^, 1395 cm^−1^, and ∼1121 cm^−1^ were attributed to the C=O stretching modes, vibrations corresponding to the C–N bonds, and C–O–C bonds, respectively. Interestingly, the peak intensities for the C–N and C–O–C bonds were low for the N-CDs extracted at 300 °C compared to that at 200 °C, which was ascribed to the partial breakdown of these bonds at a higher roasting temperature (Fig. 9a). Moreover, the XPS survey spectrum of the N-CDs synthesized at 300 °C exhibited C 1s (285.0 eV, 67.15%), N 1s (400.0 eV, 13.29%), and O 1s (531.0 eV, 18.80%) elements (Fig. 9b). The four deconvoluted peaks located at ∼284.5, 285.5, 286.4, and 288.6 eV in the C 1s core level peak are assigned to the C=C, C–N, C–O, and C=O bonds, respectively (Fig. 9c), while the high-resolution N 1s spectrum (Fig. 9d) showed two peaks at ∼398.1 eV (amide N bonds) and ∼398.6 eV (pyridinic N bonds). Many characterization techniques such as powder X-ray diffraction (PXRD), high-resolution transmission electron microscopy (HRTEM), and Raman spectroscopy are applied to investigate the crystalline nature and defect density of CDs. The PXRD spectra of CDs and doped CDs synthesized from animal/human biomass typically exhibit a diffraction peak in the 2*θ* range of 20° to 27°, which corresponds to the (002) graphitic plane. For example, the PXRD pattern of chicken cartilage-derived N-CDs showed an intense peak at 2*θ* = 23.2° due to the (002) graphitic planes (Fig. 9e).^217^ Moreover, a nearly spherical shape (Fig. 9f), narrow size distribution (Fig. 9g), and high crystallinity with 0.24 nm lattice spacing corresponding to the (100) graphitic plane (inset of Fig. 9f) were also revealed for these N-CDs by TEM and HRTEM analyses.^217^ Most animal/human biomass-derived CDs or their doped counterparts exhibit spherical/quasi-spherical shapes with a size of less than 10.0 nm (Table 1); however, the synthesis of large-sized CDs from animal biomass has also been reported. For example, CDs derived from silkworm chrysalis (∼19.0 nm),^140^ goose/chicken feathers (∼21.5/35.0 nm),^205,206^ pigeon manure (∼15.65 nm),^221^ fish scale (∼61.0 nm),^227^ and shark cartilage (∼50.0 nm).^232^ Biogenic CDs with an average size of above 10 nm were also prepared using human biomass such as human hair (∼11.0/78.0 nm)^242^ and human urine (UPDs: 20.6 nm, CPDs: 11.4 nm, and APDs: 38.8 nm).^244^ Generally, the Raman spectrum of carbonaceous nanomaterials including CDs comprised of a G band (first-order Raman band due to the in-plane vibration of sp^2^ carbon) and D band (defect centers induced by sp^3^ carbon). A high *I*_D_/*I*_G_ ratio in nano-carbon indicates the presence of disordered structure. For example, the Raman spectra of OCDs and MCDs synthesized from human hair showed two typical peaks at ∼1316 cm^−1^ (D band) and ∼1584 cm^−1^ (G band) with *I*_D_/*I*_G_ ratios of 1.12 and 1.14, respectively (Fig. 9h), suggesting the disordered structures of nano-carbon. Moreover, the shoulder peak at ∼1200 cm^−1^ in the spectrum of OCDs (dotted circle in Fig. 9h) is ascribed to the π–π charge transfer between OCDs and NO rings.^242^ The molecular weight and various functional groups/minute structural features of salmon fish-derived CDs were confirmed by matrix-assisted laser desorption ionization time-of-flight mass spectrometry (MALDI-TOF-MS) and ^1^H nuclear magnetic resonance (NMR) and ^13^C NMR spectra.^163^ The predominant peak at 1056 *m*/*z* indicated the small size of the isolated CDs (Fig. 9i). Moreover, the ^1^H NMR signals at 1.23, 2.96, and 3.12 ppm were attributed to the alkyl, R^3^ N–C–H, and –O–C–H protons, while the peaks in the range of 3.82–4.1 ppm and 7.0–9.0 ppm were ascribed to the protons attached to the alcohol groups and aromatic protons, respectively (Fig. 10a). The peaks in the range of 10–30 ppm, 30–40 ppm, 40–65 ppm, 65–90 ppm, and 110–180 ppm in the ^13^C NMR spectrum are assigned to the carbon of the –CH_3_ groups, carbon from the ≡CH/≡C–/–C=C–O– groups, carbon in the –C=N–/=N–CH_3_/≡C–N– structures, sp^3^ carbon of alcohols/ether groups, and aromatic carbon, respectively (Fig. 10b).

### 6.2 Optical properties

**Fig. 11:**
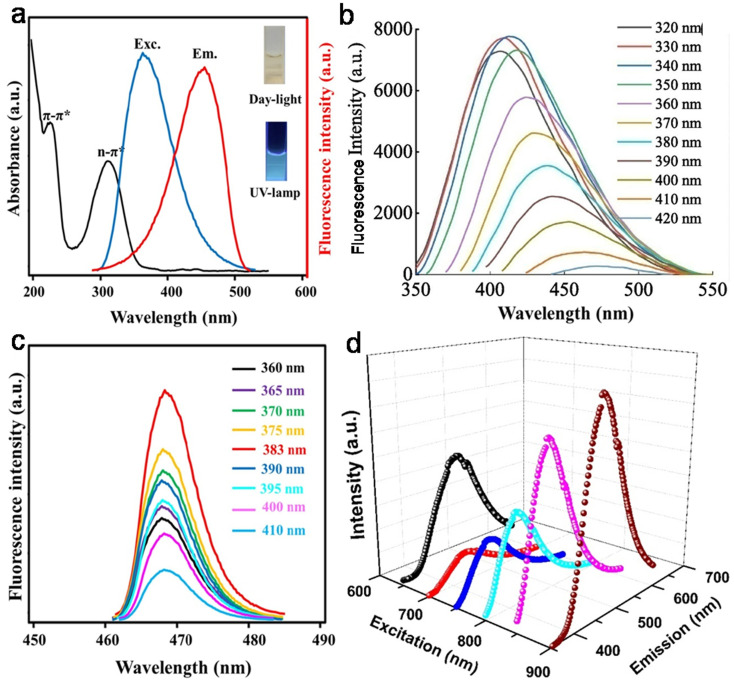
(a) The absorption (UV-vis) and (c) EWID fluorescence spectra of N,S-CDs derived from a mixture of milk powder and methionine (the inset of (a) the aqueous dispersion of N,S-CDs under day and UV light). Reprinted from ref. 200, Copyright (2023), with permission from Elsevier. (b) The EWD PL behavior of N-CDs synthesized from egg yolk. Reprinted from ref. 137, Copyright (2022), with permission from Elsevier. (d) The UCPL spectra of *Bombyx mori* SF-derived N-CDs. Reprinted from ref. 144, Copyright (2020), with permission from Elsevier.

The remarkable optical features of CDs such as their absorption, emission wavelength, excitation-tunable emission, QY, lifetime, photo and chemical stability are crucial for their potential applications. Table S1† clearly reveals that most animal/human biomass-derived CDs are comprised of a distinct absorption in the UV range (200–400 nm) and tails extended into the visible region. The lower wavelength peak is mainly due to the core domain of CDs (featured by the sp^2^-hybridized carbon framework of conjugated π electrons), and generally recognized as the π → π* transitions of the extended aromatic rings and C=C segments (Table S1†). Meanwhile, the peak/shoulder at a relatively higher wavelength in the UV region is correlated with the low energy n → π* transitions of C=O bonds/other functional groups attached to the surface or trapped excited state energy on the surface/edge (Table S1†). Therefore, it can be concluded that irrespective of the animal/human biomass utilized as the starting precursor, the optical absorption of CDs originates from their core and/or surface defects/surface states/functional groups. For example, Mohamed *et al.*^200^ observed two sharp absorption peaks at ∼230 nm and ∼315 nm, corresponding to the π → π* transitions of C=C and n → π* transition of the C=O bonds in N,S-CDs derived from a mixture of milk and methionine (Fig. 11a). Additional modifications in the surface functional groups are also directed to improve the optical absorption of CDs. For example, when crab shell-derived N-CDs were conjugated with FA *via* EDS/NHS coupling reaction, it resulted in strong peak at ∼210 nm and higher absorption at ∼280 nm in comparison to the bare N-CDs (*λ*_abs_ = 260 nm).^179^

PL is one of the most fascinating features of CDs, which determines their utility in sensing and bioimaging applications. Particularly, the excitation-wavelength dependent (EWD) emission of CDs is important for multicolor imaging applications. The PL emissions in most animal/human biomass-derived CDs exhibit the typical Stokes-type behavior (down-conversion, Table S1†). It was observed that some animal-derived CDs showed emission spectra with good symmetry and narrow full-width at half-maxima, which were correlated with the narrower size distributions of the CDs.^130,201^ Moreover, depending on the physiochemical characteristics of animal/human biomass-derived CDs, their PL spectra may follow either EWD emission or EWID behavior (Table S1†). The PL behavior of CDs is still under debate; however, there are some hypotheses for its origin, as follows: (i) core-based emission due to the band gap transitions in the conjugated π-skeletons, which is determined by the size of the carbogenic core (quantum size effect) and degree of graphitization; (ii) surface state-based emission (or trapping of excited state energy at the surface/edge states) induced by the surface/edge defects and/or passivation of the core surface *via* the attachment of functional groups; and (iii) molecular state-type emission due to the fluorophores (free or bonded) present in the CDs. A closer look at the emissions behavior and structural features of animal/human biomass-derived CDs indicated that either of the first two factors or their combination may influence the PL feature of CDs; however, one of them may be dominant in some cases (Table S1†). Fig. 11b shows the typical profile of the EWD-dependent PL spectra of N-CDs (egg yolk derived) in the *λ*_ex_ range of 320 to 420 nm and maximum emission intensity at 340 nm excitation.^137^ Most of the animal/human source-derived CDs showed blue (inset of Fig. 11a) or blue-green luminescence under the irradiation of UV light (*λ*_ex_ = 365 nm) (Table S1†). The EWD emission is a distinct observation (Table S1†); however, EWID emissions^135,145,185,200,212,215,227,231^ or the combination of both EWD and EWID^223,225^ were also noticed in animal biomass-derived CDs (Table S1†). For example, recently synthesized N,S-CDs (from a mixture of milk powder and methionine) exhibited EWID emission (*λ*_em_ = ∼ 470 nm) in the *λ*_ex_ range of 360–410 nm and maximum emission intensity at *λ*_ex_ = 383 nm (Fig. 11c).^200^ Interestingly, Ca-self-doped CDs specifically derived from animal bones (without additional purification steps) showed EWID behavior.^212,215^ The authors speculated that the functional groups attached to the surface of the CDs effectively passivated the surface defects, and therefore the EWID characteristics originated from the single radiative transition of the core sp^2^ carbon.

#### 6.2.1 Up-conversion PL (UCPL)

In addition to common down-conversion fluorescence, UCPL emission is a newly discovered phenomenon that has also been observed in animal/human biomass-derived CDs (Table S1†). It is an anti-Stokes process in which the emitted photons have higher energy (shorter emission wavelength) than the excited photons energy (longer excitation wavelength) and often interpreted by the multiple photon activation mechanism.^190,211,223,225,239^ The UCPL behavior in animal/human biomass-derived CDs was first observed in early 2013 in *Bombyx mori* silk-synthesized N-CDs.^138^ Subsequently, Wang *et al.*^144^ observed the UCPL property in N-CDs derived from the same bio-precursor, which showed red shifts in their emission peaks with an increase in *λ*_ex_ from 650 nm to 900 nm (Fig. 11d). UCPL behavior was also observed in CDs derived from many other animal/human biomass such as human hair,^239^ honey,^166^ milk,^190^ pig skin collagen,^211^ and fish scales^223–225^ (Table S1†). The UCPL of these biogenic CDs may open a new development in bioscience as a cellular imaging platform with two-photon luminescence microscopy and design of efficient catalysts for energy technology.

#### 6.2.2 Stability (effect of concentration, light, temperature, pH, solvent, and ionic strength)

The effect of concentration and various environmental factors such as exposure to light, temperature, pH, and ionic strength on the PL response of animal/human biomass-derived CDs has been routinely investigated to establish their wide applicability. The optimum concentration of CDs is always required to obtain a high PL intensity. For example, recently reported N-CDs (egg white derived) showed the maximum PL intensity at the optimum solution concentration (0.1%), while a further increase in concentration resulted in a decreasing trend due to the inner filter effect (IFE) and aggregation-based quenching. Moreover, the gradual red shift in *λ*_em_ from 441 nm to 498 nm with higher concentrations of N-CDs (0.1% to 0.5%) was explained due to the increase in H-bonding between the surface-attached functional groups, and therefore decrease in highest occupied molecular orbital-lowest unoccupied molecular orbital energy gap.^133^

The photo-bleaching resistivity of CDs is a desirable criterion for reliable fluorescent nanoprobes, and therefore frequently analyzed for animal/human source-derived CDs after exposure to UV/xenon light. Interestingly, the doped CDs isolated from grilled hamburger,^149^ baked lamb,^153^ and roasted chicken^158^ at higher temperature had better stability against photo-bleaching due to their smaller size and less functional groups. When bee pollen-derived N-CDs were exposed to a high-power UV source (24 h), they showed a reduction in PL intensity by up to 50.0% without much change in their absorption.^172^ This unique phenomenon was explained by the generation of ˙OH radicals, which effectively destroyed the surface emissive centers in N-CDs (responsible for their emission) rather than the nuclear states (mainly cause absorption). The recently reported doped CDs from crayfish shell,^186,187^ milk powder,^200^ fish scales,^231^ and fish scale collagen peptides^234^ also showed excellent photo-stability. The photo-stability of animal/human biomass-derived CDs was also demonstrated for different period of times in air^134,150,172,174,183,247^ and even after storage for many days/months/year under ambient conditions.^126,136,184,185,193,204,205,208,212,221,224,235,237,239,247^ The doped CDs obtained from spider silk,^142^ milk,^189^ pork,^210^ and pork liver^213^ showed insignificant changes in PL intensity even after long-term storage at 4 °C. Noticeably, the N-CDs derived from silkworm chrysalis,^140^ grilled fish,^150^ and pork^210^ maintained their fluorescence properties even after drying, which is vital for invisible ink application.

Meanwhile, the fluorescence intensity of doped CDs synthesized from different animal/human sources such as *Bombyx mori* silk,^141^ bee pollen,^167,172^ chicken cartilage,^217^ and human hair^243^ was barely affected by temperature, while doped CDs derived from cow milk,^198^ expired milk,^202^ pork liver,^213^ chicken bone,^218^ and cow manure^219^ showed temperature-dependent PL behavior. For example, the decreasing trend of PL intensity with an increase in temperature (27–77 °C) for cow milk-derived N-CDs was attributed to the thermal activation of non-radiative trapping sites, which resulted in an increase in non-radiative relaxation at elevated temperatures.^198^

The fluorescence intensities of the CDs derived from many animal/human biomass were very stable in a broad pH range. Moreover, a linear dependence was also observed in some animal-derived doped CDs,^127,139,180^ which is crucial for pH sensor application. The pH-dependent PL features of doped CDs derived from various animal/human sources such as egg white,^133^*Bombyx mori* silk,^138^ cocoon silk,^139^ spider silk,^142^ roasted duck,^156^ bee pollen,^172^ prawn shell,^174^ crayfish shell,^185–187^ mussel seafood,^188^ cow milk,^191,199^ hen feathers,^207^ pig skin collagen,^211^ pigeon manure,^221^ fish scales,^223,227^ carp roe,^233^ human hair,^239,240^ human urine,^245^ and human fingernails^247^ were often explained by the protonation–deprotonation of the functional groups present on their surface. The protonation–deprotonation mechanism of doped CDs under different pH conditions were also endorsed by the changes in their zeta potential.^138,140,207,239^ For example, Zn/Mg-doped CDs (derived from hen feathers) showed a positive shift in zeta potential (−34.5/−36.0 mV to −0.145/−1.67 mV) when the basic pH changed to acidic due to the protonation of available surface sites. Consequently, the PL intensity dropped in acidic pH, which is ascribed to the photo-induced electron transfer (PET) and interaction of the lone pair electrons in the different N and metal-doped sites.^207^

A variety of solvents also had a significant effect on the fluorescence behavior (intensity and/or *λ*_em_) of animal/human biomass-synthesized CDs due to their different degrees of solubility/dispersibility.^126,129,132,133,138,167,172,198,201,239,240^ In this case, the dielectric constant, refractive index, and coordination ability of the solvent may influence the fluorescence property of CDs. For example, polar protic solvents such as water, methanol, and ethanol resulted in high PL intensities in comparison to polar aprotic solvents (acetonitrile and acetone) for pork rib bone-synthesized doped CDs due to the H-bonding interaction.^212^ Additionally, animal/human biomass-derived CDs exhibited stable PL features in different solvents,^141,166^ which may be vital for their implementation in complex systems. Meanwhile, most animal/human biomass-derived CDs were not influenced much by salt solutions of different ionic strengths, indicating non-aggregative and salt tolerance nature.

#### 6.2.3 Fluorescence lifetime (*τ*)

Time-resolved or pulsed-laser excitation PL measurement is performed to assess the nanosecond *τ* and origin of fluorescence in fluorescent nanoparticles (NPs), where *τ* is the characteristic time required by a number of excited fluorophores to come back to a lower state *via* the radiative loss of energy.^250^ A short average *τ* (*τ*_av_) of animal/human biomass-derived CDs suggests that their luminescence mechanism is based on the radiative recombination of electron–hole pairs (excitons)^138,151,152,177,189,191,193,201,239^ or singlet state emission.^144^ Interestingly, the N-CDs extracted from pike eel at various roasting temperatures (160 °C, 200 °C, 230 °C, 260 °C, and 300 °C) showed a continuous increase in *τ*_av_ (5.64, 6.13, 6.89, 7.02, and 7.17 ns), and correspondingly QY (12.86%, 31.35%, 42.10%, 50.70%, and 80.16%), which is ascribed to the formation of stable excited states at a higher temperature, respectively.^151^ A similar temperature effect on *τ*_av_ and QY was also noticed for N-CDs derived from chicken,^158^ pork,^164^ and mussel seafood.^188^ Furthermore, it was observed that the decay curves of animal/human biosource-derived CDs exactly fitted either mono-exponential or bi-/tri-exponential decay kinetics (Table S1†). Multiple *τ* means the involvement of different energy levels, defect states, and surface fluorophores, resulting in diverse recombination states of excitons.^130,158,207,211,225,242^ For example, the three components of *τ* in the case of human hair-derived OCDs/MCDs are attributed to the radiative transitions from three different emissive centers, *i.e.*, σ*/π* → n (*τ*_1_: associated with the surface functional groups), π* → π (*τ*_2_: due to the carbogenic core), and π* → π *via* mid-gap states (*τ*_3_: originated from the functional groups and defects). Interestingly, the third component (*τ*_3_) is prolonged for OCDs (11.33 ns) in comparison to MCDs (8.36 ns), suggesting that OCDs had deeper trap states.^242^

## 7 Applications of animal/human biomass-derived CDs

Due to the unique properties of animal/human biomass-derived CDs such as small size, abundant surface functionality, low toxicity, aqueous solubility/dispersibility, and biocompatibility, they may function as excellent nanoprobes for sensing and biological applications.

### 7.1 Sensor application

**Fig. 12:**
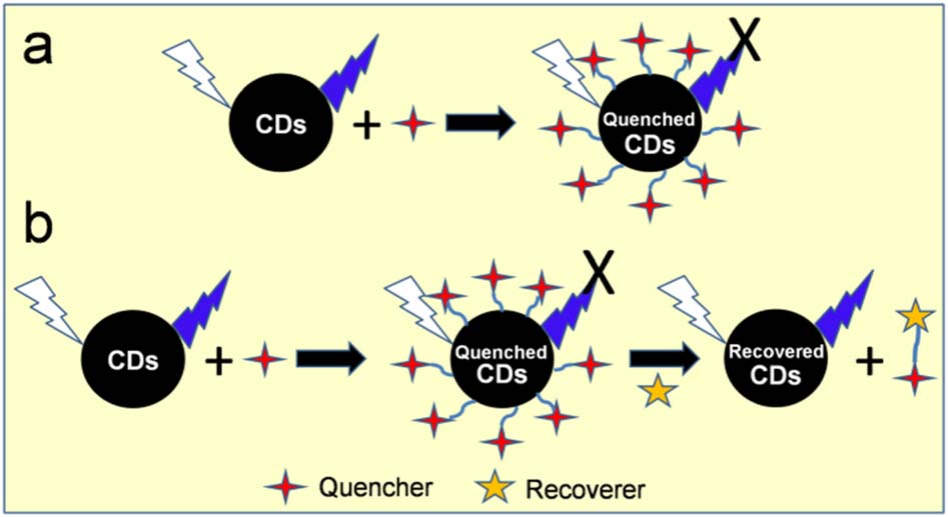
A schematic illustration of the fluorescence sensing strategy: (a) turn on–off and (b) turn-on–off–on.

When foreign species/analytes (metal ions, biomolecules, drugs, *etc.*) come in contact with CDs, they preferably interact with their surface functional groups, resulting in either a reduction/enhancement in their emission intensity (light-down/light-up) or shift in their PL peak or change in their absorption behavior. Therefore, it is desirable for the optical signal of CDs to be altered after the addition of the analyte to construct an efficient sensor probe. In this case, “turn on–off” of the fluorescence signal is the most common approach (Fig. 12a); however, the “turn on–off–on” approach (Fig. 12b) is also employed to design sensor platforms using animal/human biomass-derived CDs (Table 2). Moreover, the involvement of several quenching mechanisms in the sensing process such as static quenching effect (SQE), dynamic quenching effect (DQE), fluorescence resonance energy transfer (FRET), IFE, electron transfer (ET), aggregate-induced quenching (AIQ), coordination/interaction-induced quenching (CIQ/IIQ), and PET is also revealed in Table 2.

**Table 2:** A summary of sensing parameters and mechanism of animal/human biomass-derived CDs for various analytes^a^

Source	Sensing process/mechanism	Linear range (μM)	LOD (μM)	Analyte	Ref.
**Metal ions sensor**					
Egg shell membrane	Turn on–off–on, ET	—, 0.5–80	—, 0.48	Cu^2+^, GSH	127
Prawn shell	Turn on–off, CIQ, IFE	0.01–1.1	0.005	^c^Cu^2+^	174
Human fingernail	Turn on–off, CIQ	0–1.0	0.001	Cu^2+^	249
Milk powder + methionine	Turn on–off–on, FRET, IFE, DQE	0.01–55, 5.02–883	0.003, 1.48	^c^Cu^2+^, ^c^risedronate sodium	200
Honey	Turn on–off, AIQ	0.005–100	0.0017	^c^Fe^3+^	166
Milk	Turn on–off, CIQ, ET	10–150	—	^c^Fe^3+^	193
Goose feather	Turn on–off, CIQ, SQE, DQE	2–7	0.196	^c^Fe^3+^	205
Egg white	Turn on–off, IIQ	50–250	—	Fe^3+^	129
Egg white	Turn on–off, ET	0–25	0.54	Fe^3+^	132
Sheep wool	Turn on–off, CIQ, ET	0.1–10.0	0.01	Fe^3+^	235
*Bombyx mori* silk + CA	Turn on–off, AIQ	0.5–4.0	0.38	Fe^3+^	141
Expired milk	Turn on–off, CIQ	500–1400	—	Fe^3+^	202
Fish scale (crucian carp)	Turn on–off, CIQ, ET, SQE	1–78	0.54	^c^Fe^3+^	225
Fish scale (*Ctenopharyngodon idella*)	Turn on–off, IIQ	0–6.25	0.144	^c^Fe^3+^	227
Bee pollen (water)/(ethanol)	Turn on–off, CIQ, ET, DQE	50–100/20–100	27.0/15.0	Fe^3+^	172
Cow milk	Turn on–off, CIQ, ET	0.1–20, 20–100	0.6	^c^Fe^3+^	199
Pig skin	Turn on–off, IFE, SQE, DQE	1–100	0.68	Co^2+^	208
Hair	Turn on–off, CIQ, SQE, DQE	0–75	0.01	^c^Hg^2+^	241
Pigeon egg white/yolk	Turn on–off, CIQ, IFE, ET	0.05–1.2/0–1.6	0.0346/0.0349	Hg^2+^	134
Urine + citron juice	Turn on–off–on, ET, DQE	0–240, 1–10	0.15, 0.04	^c^Hg^2+^, ^c^Cysteine	245
Spider silk	Turn on–off, CIQ, SQE	0–0.08	0.0053	^c^Hg^2+^	142
Fish scale (grass carp)	Turn on–off, CIQ, ET	0–30	0.014	^c^Hg^2+^	224
Pigeon manure	Turn on–off, CIQ	12–120	18.5	^c^Hg^2+^	221
Carbonized hen feather + Zn salt	Turn on–off, ET, AIQ, DQE	0–6^a^, 8–20^a^	—	Hg^2+^	207
Carbonized hen feather + Mg salt	Turn on–off, ET, AIQ, DQE, SQE	0–6^a^, 12–20^a^	′′	′′	
Sheep wool/pig hair	Turn on–off, CIQ, ET	0.05–100/same	0.0168/0.0296	^c^Cr^6+^	237
Fingernail	Turn on–off, IFE, SQE	0.0017–0.0675	0.0003	^c^Cr^6+^	248
Denatured milk	Turn on–off, IFE	5–100	14.0	^c^Cr^6+^	203
Shrimp shell	Turn on–off, IFE, ET	0–70	0.1	^c^Cr^6+^	183
Denatured sour milk	Turn on–off, IFE, SQE	10–150	0.95	^c^Au^3+^	204
Crab shell	Turn on–off, IIQ	50–250	—	Cd^2+^	181
Cow milk	Turn on–off, CIQ, SQE, DQE	0–50	17	Sn^2+^	198
Urine-unmodified diet	Turn on-off, CIQ	0–50, 0–30	2.7, 3.4	Hg^2+^, Cu^2+^	244
Urine-asparagus rich diet	′′	′′	2.7, 2.9	′′	
Urine-vitamin C supplemented	′′	′′	1.8, 1.7	′′	
Pigeon feather	Turn on–off, CIQ, IFE, ET	0–0.12, 0–1.6	0.0103, 0.0609	Hg^2+^, Fe^3+^	134
Bovine/pork/sheep bones	Turn on–off, CIQ, SQE	—	0.04–4.0	^c^Ag^+^, ^c^Cu^2+^, ^c^Hg^2+^, ^c^Pb^2+^, ^c^Fe^3+^	215
Wool keratin	Turn on–off, CIQ, IFE, SQE	2.5–50	0.01416	^c^Cr^6+^	238
	Turn on–off, CIQ, DQE	0.25–125	0.113	Fe^3+^	

**Other analytes sensor**					
Fish scale (grass carp)	Turn on–off, PET	0–1000	—	ClO^−^	222
Lamb	Turn on–off	0–100	—	H_2_O_2_	152
	′′	10–300	2.9	^c^Glucose	
Cow manure	PL enhancement, coordination	10^5^–10^6^	—	Glucose	220
Chicken cartilage	Turn on–off, DQE	2–500, 500–1500	0.47	H_2_O_2_	217
	Turn on–off	5–1000	1.22	^c^Glucose	
Pork	Turn on–off, CIQ, AIQ, SQE	0.1–100, 100–500	0.05	^c^UA	210
Milk powder + FeCl_3_	Turn on–off–on, IFE, IFE inhibition	20–500	5.13	^c^AA	196
Mussel seafood	Turn on–off, FRET	1–50	0.00606	^c^Riboflavin	188
Prawn shell	Turn on–off, Redox reaction, DQE	0–1000	1.0	^c^NO_2_^−^	175
Sheep wool	Turn on–off–on, IFE, IFE inhibition	0.148–14.8	0.071	^c^Glyphosate	236
Pork rib bone	Turn on–off–on, SQE, DQE, FRET	0.15–5.0	0.064	^c^Dimethoate	212
Crayfish shell	Turn on–off–on, oxidation, DQE, oxidation inhibition	10–300	2.30	^c^PCP	186
Egg white	Turn on–off, IIQ, FRET	0.78–50	0.04	^c^Curcumin	131
Fish scale (grass carp)	Turn on–off, CIQ, SQE	185–1295	54.0	^c^LH	223
Crab shell	Turn on–off, IIQ, SQE	0.02–1.0	0.009	^c^CFX	180
Chicken drumstick	Turn on–off, IFE	0.0009–0.014	0.00044	^c^CFX	214
Milk	Turn on–off, IFE	2–200	0.6	^c^TCs	195
Carp roe	Turn on–off, IFE, SQE	0.1–50	0.0417	^c^TCs	233
Pork liver	Turn on–off, CIQ, IFE, SQE	1–200	0.75	^c^6-TG	213
GGEC	Turn on–off, IFE, SQE	0.267–100	0.267	^c^MNZ	216
	′′	0.067–80	0.067	^c^TNZ	
	′′	0.133–200	0.133	^c^ONZ	
	′′	0.067–200	0.067	^c^SNZ	
Crayfish shell	Turn on–off, CIQ, SQE	0.14–0.84	0.077	^c^TAP	185
Hair	Turn on–off, IFE, SQE	0.613–306.6	0.205	^c^CLZ	243
Fingernail	Turn on–off, IFE, SQE	0.0003–0.0434	0.0001	^c^SY	247
Egg white	PL enhancement, PET	0.609–6.09	0.092	^c^RK	133
Hair	PL enhancement, coordination	0.0001–0.05^a^	0.003^a^	CHCl_3_	242
Crayfish shell	Turn on–off, CIQ, SQE	0–50	0.16	^c^4-NP	187
Carbonized hen feather + Zn salt	Turn on–off, IFE	0–20^a^	—	4-NP	207
Carbonized hen feather + Mg salt	′′	′′	—	′′	
Chicken bone	PL enhancement, interaction	0–10^b^	0.17^b^	^c^Liby	218

a ^a^Parts per million, ^b^μg mL^−1^, and ^c^applicability for real sample analysis.

#### 7.1.1 Metal ion sensor

##### 7.1.1.1 Copper ion (Cu^2+^) sensor

**Fig. 13:**
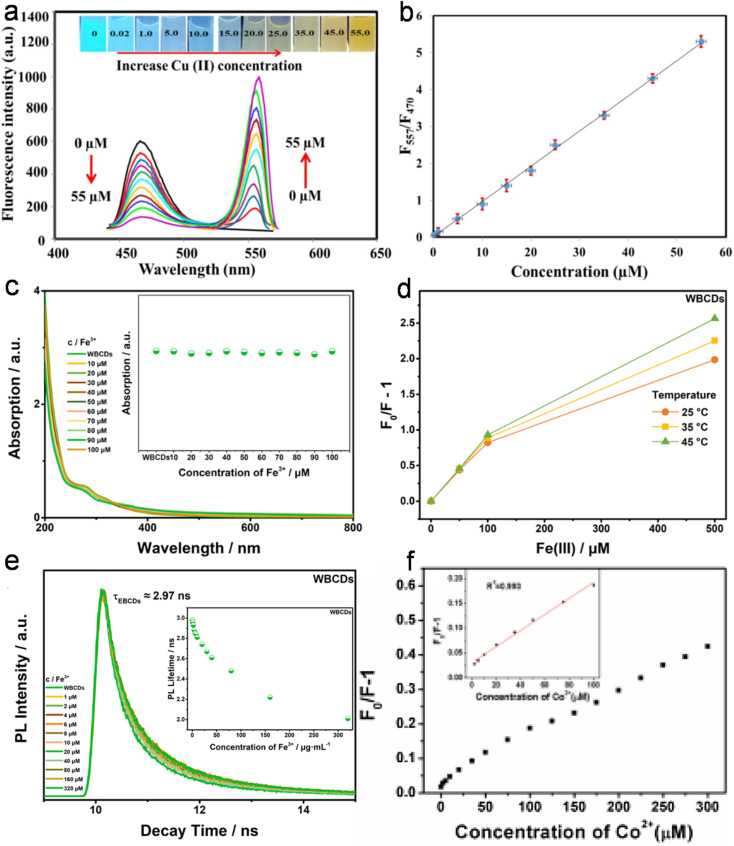
(a) Variation in the emission intensity of the sensor assembly (N,S-CDs and *o*-phenylenediamine) with different concentrations of Cu^2+^ (inset: the digital photograph of sensor system under UV-light after applying Cu^2+^ in different concentrations) and (b) the corresponding linear plot of 557/470 nm emission intensity (*F*_557_/*F*_470_) with respect to Cu^2+^ concentration. Reprinted from ref. 200, Copyright (2023), with permission from Elsevier. (c) The UV-vis absorption spectra (inset: variation in absorption intensity), (d) temperature-dependent Stern–Volmer curves, and (e) PL decay profile (inset: variation in the measured lifetime) of bee pollen-derived WBCDs with different concentrations of Fe^3+^. Reprinted from ref. 172, Copyright (2022), with permission from Elsevier. (f) The non-linear plot of (*F*_0_/*F* − 1) by applying different concentrations of Co^2+^ on pig skin-derived N-CDs (inset: linearity in the Co^2+^ concentration range of 1–100 μM). Reprinted from ref. 208, Copyright (2016), with permission from Elsevier.

The fluorescence quenching of egg shell membrane-synthesized doped CDs in the presence of Cu^2+^ and restoration of their fluorescence signal after the addition of glutathione (GSH) were probably the first report where a metal ion (Cu^2+^) quenched the fluorescence signal of animal/human biomass-derived CDs.^127^ Subsequently, prawn shell-^174^ and human fingernail-^249^derived CDs were employed for the selective detection of Cu^2+^ with an appreciable limit of detection (LOD) (Table 2). Recently, the fluorescence signal of N,S-CDs (milk powder-derived; *λ*_em_ = 470 nm) was quenched by Cu^2+^/*o*-phenylenediamine through FRET and IFE due to the formation of 2,3-diaminophenazine (oxidized product of *o*-phenylenediamine, *λ*_em_ = 557 nm) (Fig. 13a), which resulted in the sensitive detection of Cu^2+^ in the linear range of 0.01–55 μM (Fig. 13b) and LOD as low as 3.0 nM. Moreover, the sensing platform could recognize risedronate sodium (bisphosphonate-containing drug) in a wider linear range of 5.02–883 μM with the LOD of 1.48 μM by the recovery of its fluorescence signal due to the chelation of the drug molecules with Cu^2+^ and inhibition of the oxidized product. The sensing platform was also found to be applicable for the detection of Cu^2+^ and risedronate sodium in real water and biological fluids.^200^

##### 7.1.1.2 Ferric ion (Fe^3+^) sensor

Fe^3+^ is the most common metal ion, which has been detected by animal/human biomass-derived CDs due to its strong affinity with their surface functional groups. Earlier reports showed the Fe^3+^ detection ability of CDs derived from honey,^166^ milk,^193^ goose feathers,^205^ egg white,^129,132^ sheep wool,^235^*Bombyx mori* silk,^141^ expired milk,^202^ and fish scales,^225,227^ involving various quenching mechanisms and different LODs in the range of 1.7–540 nM (Table 2). Subsequently, bee pollen-derived N-CDs (WBCDs and EBCDs) were implemented for the rapid, sensitive, and selective detection of Fe^3+^*via* fluorescence quenching, which was attributed to the transfer of electrons from the excited state of N-CDs to the vacant Fe^3+^ d-orbital through a non-radiative process. The involvement of DQE in the quenching process was verified by the following characteristics: (i) no changes in the absorption peak and intensity with different Fe^3+^ concentrations because the collision between N-CDs and Fe^3+^ occurs in the excited state (Fig. 13c), (ii) increase in quenching constant (measured from Stern–Volmer plot) with an increase in temperature (Fig. 13d), and (iii) decrease in *τ*_av_ after the addition of Fe^3+^ in comparison to the bare N-CDs (Fig. 13e).^172^ Recently, cow milk-derived N-CDs were also applied for the sensitive and selective detection of Fe^3+^*via* a non-radiative ET process with a satisfactory LOD (0.6 μM).^199^

##### 7.1.1.3 Cobalt ion (Co^2+^) sensor

Although cobalt is an essential trace element in biological systems, its excessive intake may have adverse health effects. N-CDs (derived from pig skin) were used for the selective/sensitive detection of Co^2+^ (linear range: 1–100 μM and LOD: 0.68 μM). The quenching of their fluorescence signal was due to both SQE and DQE because the plot of (*F*_0_/*F* − 1) *vs.* Co^2+^ concentration (1–300 μM) did not show a linear fitting in the entire concentration range (Fig. 13f). Moreover, the IFE-based fluorescence quenching was justified by the precise overlap of the Co^2+^ absorption band (400–550 nm) with the excitation/emission bands of N-CDs (460/521 nm; fluorophore).^208^

##### 7.1.1.4 Mercury ion (Hg^2+^) sensor

The detection of Hg^2+^ is vital due to its serious toxic effect in living systems. Doped CDs derived from human hair,^241^ pigeon white/yolk,^134^ human urine,^245^ spider silk,^142^ and fish scales^224^ have been applied for the selective/sensitive detection of Hg^2+^ due to their strong affinity with attached functional groups. Interestingly, the LOD of Hg^2+^ with spider silk-derived N-CDs was very low (5.3 nM) and the sensor probe could also be applied in the real water samples.^142^ Subsequently, pigeon manure-derived N-CDs were also used for the detection of Hg^2+^*via* CIQ.^221^ Recently, hen feathers were utilized to develop a Zn/Mg-doped CD-based sensor platform. It was observed that the fluorescence quenching of Mg-CDs (70%) was higher than that of Zn-CDs (52%) in the presence of 20 ppm Hg^2+^, which is primarily governed by DQE. Moreover, the significant involvement of SQE in the higher linear range (12–20 ppm) resulted in the better quenching response of Mg-CDs.^207^

##### 7.1.1.5 Chromium ion (Cr^6+^) sensor

The development of sensor probes for Cr^6+^ is important due to its severe toxicity in humans and the ecosystem. In this case, doped CDs derived from sheep wool/pig hair,^237^ human fingernails,^248^ denatured milk,^203^ and shrimp shells^183^ showed potential to detect Cr^6+^. The quenching of the fluorescence signal in these CDs occurred either *via* ET or IFE (Table 2). Moreover, these sensing platforms were also applied for the detection of Cr^6+^-spiked real water samples with good recovery, accuracy, and sensitivity. Noticeably, the fingernail-derived N,S-CDs showed Cr^6+^ sensitivity in a wider linear range (1.7–67.5 nM) with the lowest LOD (0.3 nM).^248^

##### 7.1.1.6 Gold ion (Au^3+^) sensor

Sharma *et al.*^204^ used N-CDs (denatured sour milk derived) with AA for the selective/sensitive detection of Au^3+^ (linear range: 10–150 μM and LOD: 0.95 μM). Mechanistic investigations showed the synergistic effect of IFE and SQE in the fluorescence quenching of N-CDs/AA with Au^3+^, which is initiated by the formation of AuNPs *via* the AA-mediated reduction of Au^3+^. Besides real field water sample analyses with satisfactory recoveries and error limits, N-CD-coated paper was used for the visual detection of AA-containing Au^3+^ solutions by the varying degrees of color response under UV-light illumination.

##### 7.1.1.7 Cadmium ion (Cd^2+^) sensor

Among the harmful heavy metal ions, Cd^2+^ is widely spreading in the environment due to the chemical industry and human activities. In this case, crab shell-derived CDs have been used for the selective detection of Cd^2+^. Here, the fluorescence quenching ability of Cd^2+^ in the concentration range of 50–250 μM is promoted by strong interaction between Cd^2+^ and CDs.^181^

##### 7.1.1.8 Tin ion (Sn^2+^) sensor

The sensitive and selective detection of Sn^2+^ is crucial because of its adverse effect in the human digestive and respiratory systems. Kumar *et al.*^198^ applied cow milk-derived N-CDs for the selective and sensitive detection of Sn^2+^ with a good LOD (17 μM). Here, the strong interaction between Sn^2+^ and N-CDs induced the formation of a complex, which resulted in fluorescence quenching *via* non-radiative recombination. Moreover, the involvement of SQE and DQE is based on the non-linear fitting of *F*/*F*_0_ in the entire concentration range of Sn^2+^ (0–900 μM).

##### 7.1.1.9 Multi-metal ion sensor

**Fig. 14:**
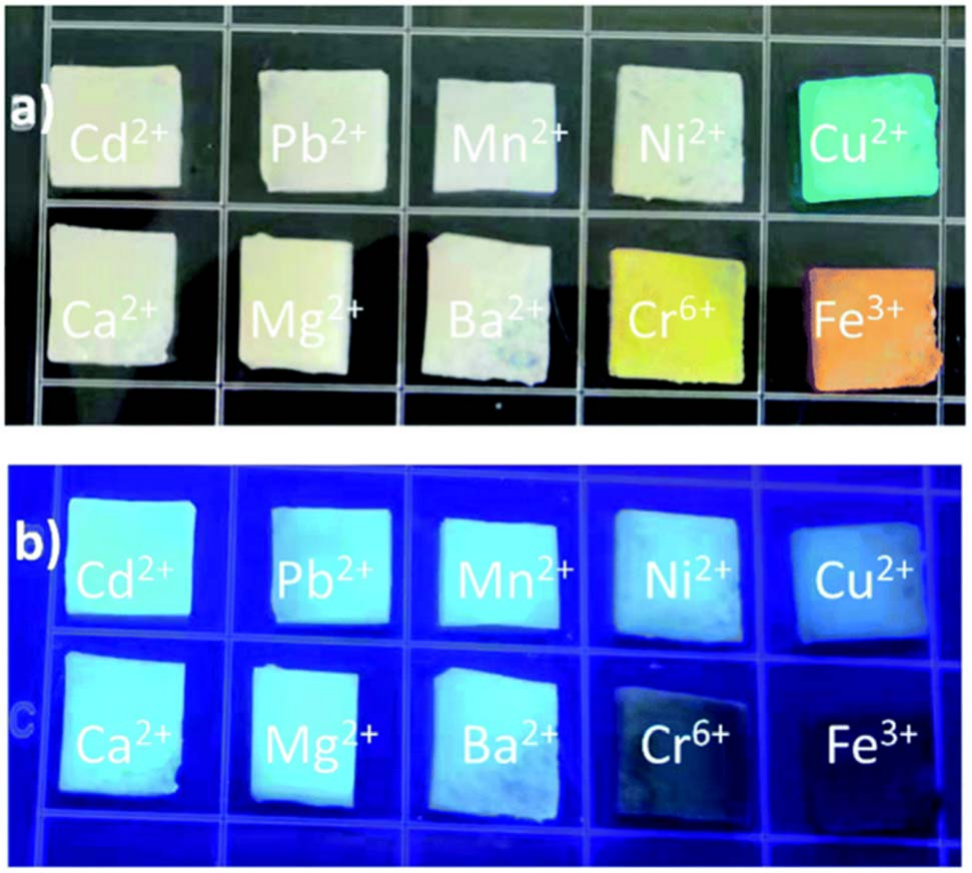
The photographs of wool keratin-derived N,S-CD-hydrogel incubated in different metal ion solutions under (a) day light and (b) UV irradiation. Reproduced/adapted from ref. 238 with permission from The Royal Society of Chemistry, 2022.

Besides single metal ions, multiple metal ions have also detected using animal/human biomass-derived CDs. For example, CDs derived from human urine^244^ and pigeon feathers^134^ were successfully employed for the selective detection of Hg^2+^/Cu^2+^ and Hg^2+^/Fe^3+^ metal ions, respectively. Fu *et al.*^215^ developed hierarchical cluster and linear discriminant analysis methods to distinguish five heavy metal ions (Ag^+^, Hg^2+^, Pb^2+^, Cu^2+^, and Fe^3+^) and their binary/ternary mixture with 100% accuracy using animal bone-derived-doped CDs. Subsequently, the fluorescence intensity of wool keratin-derived N,S-CDs was quenched by Cr^6+^ and Fe^3+^. The coordination-based fluorescence quenching of N,S-CDs was ascribed to the co-existence of IFE and SQE for Cr^6+^ and DQE in the case of Fe^3+^. The detection of Cr^6+^ in a simulated electroplating wastewater solution (a mixture of Cu^2+^, Ni^2+^, Zn^2+^, and Cr^6+^) was also achieved by this sensor probe. Moreover, the N,S-CD-loaded hydrogel could visually sense both Cr^6+^/Fe^3+^ at a lower concentration upon exposure to UV-light (Fig. 14).^238^

#### 7.1.2 Other analyte sensor

##### 7.1.2.1 Reactive oxygen species (ROS) and biomolecule sensor

ROS refer to a wide variety of oxidants such as hypochlorite (ClO^−^) and H_2_O_2_, which are essential for different physiological functions.^251^ However, the detection of these species is paramount to combat their hazardous effect. Moreover, the detection of various biomolecules such as glucose, uric acid (UA), AA (vitamin C), and riboflavin (vitamin B2) is also vital for living beings. Fish scale-derived N-CDs have been employed for the selective detection of ClO^−^ in the presence of other common metal ions through the fluorescence quenching process (PET from the N elements of doped CDs to ClO^−^).^222^ Heteroatom-doped CDs derived from baked lamb,^152^ cow manure,^220^ and chicken cartilage^217^ were applied for the sensitive/selective detection of glucose. Interestingly, the PL intensity of cow manure-derived PBA-CDs increased linearly with an increase in glucose concentration (0.1–1.0 M, light-up sensor), which was ascribed to the binding effect of glucose with the electron-donor groups of boronic acid (functional groups present on the surface of PBA-CDs).^220^ Besides, the N-CDs (chicken cartilage derived)-based biosensor platforms (CDs/Fe^2+^ and CDs/Fe^2+^/glucose/glucose oxidase (GO_*x*_)) showed the sensitive and selective determination of H_2_O_2_ and glucose with a wider linear range and a lower LOD compared to the previously reported baked lamb-derived N-CDs (Table 2).^152,217^ The produced H_2_O_2_ by the catalytic conversion of glucose (in the presence of GO_*x*_) and formation of ˙OH (*via* Fenton reaction) functioned as a bridge to detect glucose *via* DQE. Also, was proposed that the highly reactive ˙OH may partially destroy the surface functional groups and conjugated structures of N-CDs, resulting in the quenching phenomenon.^152,217^ N-CDs synthesized from pork were employed for the effective and selective sensing of UA (based on the combined effect of aggregation and the formation of a ground-state complex *via* electrostatic and H-bonding interactions).^210^ Fe-CDs (milk powder derived) were used for the colorimetric/fluorometric detection of AA. The oxidized product of TMB (oxTMB produced with Fe-CDs; TMB = 3,3′,5,′-tetramethylbenzidine) effectively quenched the fluorescence signal of Fe-CDs *via* IFE, and the fluorescence further restored in the presence of AA due to the inhibition of oxTMB. Consequently, the characteristic absorption of oxTMB (∼652 nm) was reduced with the evolution of the Fe-CDs emission (∼460 nm) for the dual-signal detection of AA with a colorimetric/fluorometric LOD as low as 8.59/5.13 μM.^196^ Subsequently, a biosensor was developed based on mussel seafood-derived N-CDs for the detection of riboflavin (through FRET) with an LOD as low as 6.06 nM.^188^

##### 7.1.2.2 Anion sensor

Prawn shell-derived N-CDs were used for the fluorometric detection of nitrite ion (NO_2_^−^) in water. The fluorescence quenching-based sensor system could detect NO_2_^−^ with an LOD as low as 1.0 μM, which is much lower than the maximum permissible limit set by the World Health Organization (WHO) in drinking water (65 μM). The DQE-based quenching occurred due to the redox reaction between light-induced excited oxidation states of N-CDs and analyte. However, I^−^ is recognized as a potential interfering anion in the selectivity of NO_2_^−^.^175^

##### 7.1.2.3 Herbicide and pesticide sensor

**Fig. 15:**
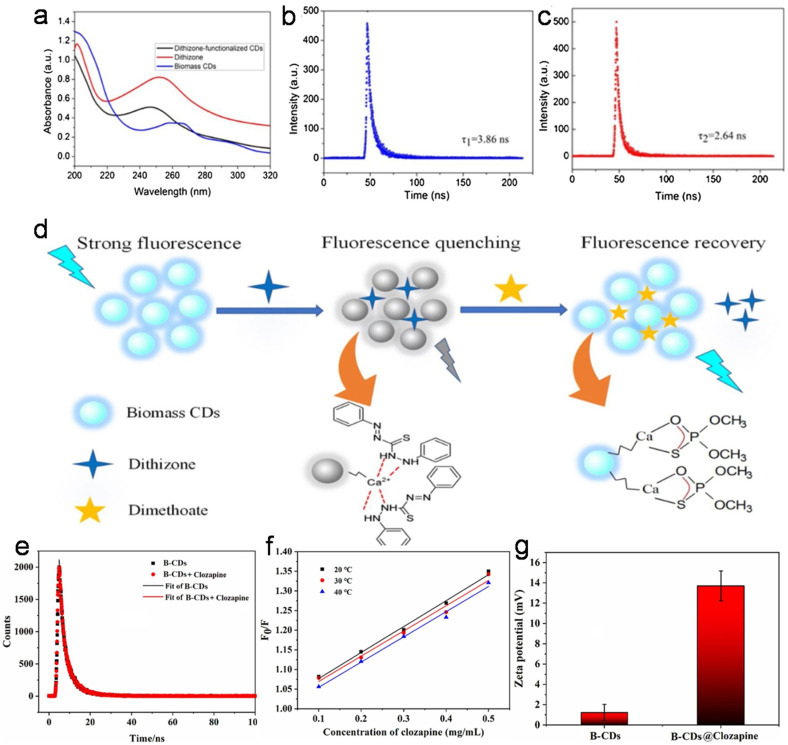
(a) The UV-vis spectra of pork rib bone-derived doped CDs, dithizone, and doped CDs-dithizone complex, (b and c) the fluorescence decay curves of doped CDs and doped CDs-dithizone complex, and (d) a schematic of dimethoate detection *via* the fluorescence turn-on–off–on mechanism. Reprinted from ref. 212, Copyright (2020), with permission from Elsevier. (e) The fluorescence decay profile, (f) temperature-dependent Stern–Volmer plots, and (g) zeta potentials of human hair synthesized N-CDs and N-CD-CLZ adduct. Reprinted from ref. 243, Copyright (2023), with permission from Elsevier.

The consumption of glyphosate as an environmentally safe herbicide is very common in agricultural and non-agricultural cultivation due to its low toxicity.^252^ However, its excessive use may lead to high levels of accumulation in the soil and agricultural products, resulting in adverse health effects in human and animals.^253,254^ Alternatively, dimethoate and pentachlorophenol (PCP) are widely used organophosphate and organochlorine pesticides, respectively, which accumulate in the ecosystem (soil, water, and agriculture products) and cause adverse toxicological impacts in humans and other living beings.^255,256^ In this case, a CD/AgNP-based fluorometric platform was developed using N,S-CDs (sheep wool derived) for the highly sensitive and selective determination of glyphosate. The fluorescence quenching of CDs by AgNPs (turn-off), and its subsequent recovery (turn-on) in the presence of glyphosate are due to the IFE and IFE inhibition, respectively.^236^ Liu *et al.*^212^ constructed a turn on–off–on-based fluorescent probe for the sensitive and selective detection of dimethoate. The dithizone-induced fluorescence quenching of doped CDs (pork rib bone derived) was attributed to the coexistence of SQE (formation of ground-state complex between dithizone and Ca element present in the CDs, as evidenced by the change in UV-vis spectrum, Fig. 15a), DQE (change in *τ*_av_ in the absence and presence of dithizone, Fig. 15b and c, respectively), and FRET effect. Furthermore, the fluorescence intensity was gradually restored after the addition of dimethoate due to the displacement of dithizone (bound at the surface of CDs) *via* its preferential bonding with the hydrolyzed product of dimethoate, which resulted in the inhibition of the quenching process (Fig. 15d). Recently, a turn on–off–on fluorescence sensor was developed using crawfish shell-derived N,S-CDs for the sensitive and selective assessment of PCP. The sensing strategy was based on DQE-type fluorescence quenching in the presence of horseradish peroxidase/H_2_O_2_ (the generated ˙OH oxidized the functional groups in N,S-CDs) and its subsequent recovery by the addition of PCP (inhibition of oxidation) with a satisfactory LOD (2.3 μM). Moreover, the sensing platform was suitable for the detection of PCP in spiked fresh crawfish, aquaculture water, and lake water samples with recoveries in the range of 95.62–105.17% and relative standard deviation (RSD) in the range of 1.20–4.50%.^186^

##### 7.1.2.4 Drug molecule sensor

Animal/human biomass-derived doped CDs have been successfully applied to detect various drug molecules such as curcumin,^131^ lidocaine hydrochloride (C_14_H_22_N_2_O·HCl, LH),^223^ ceftriaxone (CFX),^180,214^ tetracyclines (TCs),^195,233^ 6-thioguanine (6-TG),^213^ nitroimidazoles,^216^ thiamphenicol (TAP),^185^ and clozapine (CLZ)^243^*via* the turn on–off-based quenching process (Table 2). Noticeably, the LOD of CFX drug with chicken drumstick-derived N-CDs (0.44 nM)^214^ was much improved compared to the previously reported crab shell-derived N-CDs (9.0 nM).^180^ Moreover, the significant improvement in the LOD for the IFE-based detection of TCs with carp roe-derived N-CDs (41.7 nM)^233^ rather than milk-derived N,P-CDs (600 nM)^195^ is also remarkable. Besides, the sensitive and selective detection of 6-TG with N-CDs (pork liver derived) showed temperature-, pH-, and response time-dependent fluorescence quenching.^213^ Among the various nitroimidazole drugs, the LOD of tinidazole (TNZ)/secnidazole (SNZ) was found to be superior to metronidazole (MNZ)/ornidazole (ONZ) using GGEC-derived N-CDs (Table 2).^216^ Subsequently, the N,S-CDs derived from crayfish shell were employed for the sensitive detection of TAP (linear range: 50–300 μg L^−1^ and LOD: 27.5 μg L^−1^ or 0.077 μM).^185^ Recently, carbonized human hair-derived N-CDs were used as an IFE/SQE-based fluorescence quenching probe for the sensitive detection of CLZ with an LOD as low as 67 ng mL^−1^ (0.205 μM), which is far below than the minimum effective amount in the blood (0.35 μg mL^−1^). The insignificant change in *τ*_av_ (Fig. 15e) and decrease in the quenching constant with an increase in temperature (calculated from Stern–Volmer plot, Fig. 15f) endorsed that the quenching was based on SQE. Moreover, the significant increase in the zeta potential (1.229 to 13.7 mV) after the quenching process justified the coordination ability of CLZ with the functional groups attached to the surface of N-CDs (Fig. 15g). The sensing platform also exhibited satisfactory recoveries of CLZ in actual tablet (103.26%) and blood samples (92.92–102.45%).^243^

##### 7.1.2.5 Food/beverage colorant or flavoring agent sensor

Sunset yellow (SY) and raspberry ketone (RK) are a widely used food/beverage colorant and flavoring agent, respectively, which can have adverse health impacts upon excessive consumption.^257,258^ In an earlier report, human fingernail-synthesized N,S-CDs were explored as a fluorescence quenching nanoprobe (IFE and SQE based) for the sensitive detection of SY (LOD: 0.1 nM).^247^ Recently, N-CDs (egg white derived) were executed for the wider (0.609–6.09 μM) and low LOD (0.092 μM) detection of RK. Interestingly, N-CDs showed an enhancement in their absorbance and emission spectra after incubation with RK, which was ascribed to the PET between the N-CD functionalities and RK *via* radiative recombination. Moreover, the sensor was also validated for the detection of RK in dietary supplement capsules.^133^

##### 7.1.2.6 Organic molecule sensor

The strong oxidizing ability of HClO and ClO^−^ towards organic matter during the chlorine disinfection process leads to the generation of various trihalomethanes, including chloroform (CHCl_3_), as major by-products, which are potentially toxic to humans and aquatic organisms.^259^ Singh *et al.*^242^ showed the CHCl_3_ sensing capability of human hair-derived N-CDs with a low LOD of 3.0 ppb (parts per billion), which is well below the WHO guideline for drinking water (300 ppb). The CHCl_3_ molecules bound to N-CDs behaved like a passivating agent to prevent interactions between the solvent molecule (water) and electron density of the exposed pyridinic N–O groups to develop a PL enhancement-based sensor platform. Recently, crayfish shell-^187^ and hen feather^207^-derived doped CDs were employed to assemble a sensor platform for the detection of another hazardous organic molecule, namely, 4-nitrophenol (4-NP). A wider linear range of 0–20 ppm and more than 90% PL quenching of both Zn/Mg-doped CDs using 20 ppm 4-NP in acidic condition (pH = 2) clearly justified their efficiency to detect toxic pollutants in an aqueous environment.^207^

##### 7.1.2.7 Laundry powder sensor

Laundry powder is a commonly used household item, which contains phosphate and surfactant as major aquatic pollutants.^260^ Recently, a fluorescence enhancement-based sensor system was developed using chicken bone-derived CDs for the detection of laundry powder (Liby) with an appreciable LOD (0.17 μg mL^−1^). The recovery rates (85.2–106%) and RSDs (1.01–5.3%) of the sensor in spiked water samples (mineral/tap/river water) showed its applicability in real water systems.^218^

### 7.2 Bioimaging application

Compared to metal-based QDs, fluorescent CDs are found to be more suitable for bio-applications due to their small size, high water dispersibility/solubility, low cytotoxicity, superior cell viability, high photo-stability, and good bio-degradability. Multimodal bioimaging through CDs is an effective approach for diagnosis and cell labeling. It is known that CDs may have critical effects on living cells and model organisms.^261–263^ Therefore, before applying CDs in various bio-environments, it is necessary to understand their *in vitro* and/or *in vivo* cytotoxicity as well as bio-distribution and cellular uptake for safety purpose. In this section, the toxic evaluation, bio-distribution, cell uptake, and bioimaging applications of animal/human biomass-derived CDs are summarized.

#### 7.2.1 *In vitro*/*in vivo* cytotoxicity

Many animal/human source-derived CDs exhibit excellent biocompatibility and low cytotoxicity to various investigated cell lines using either the standard MTT (3-(4,5-dimethylthiazolyl-2)-2,5-diphenyltetrazolium bromide) assay or other cell counting protocols (Table S2†). Horo *et al.*^147^ evaluated the *in vitro* cytotoxicity of CDs synthesized from SF, their biotin conjugate (BT-CDs), and 5-fluorouracil-loaded BT-CDs (FU-BT-CDs) through cancerous (human breast cancer (MCF-7) and human cervical cancer (HeLa)) and normal (human embryonic kidney cells (HEK-293)) cell lines. The results showed the higher cytotoxicity of FU-BT-CDs in the cancer cell lines compared to the normal cell line, which signified the positive/negative degree of biotin expression in the cancer/normal cell lines. Furthermore, the large value of IC_50_ in HEK-293 (138.1 μM) compared to MCF-7 (28.19 μM) and HeLa (38.17 μM) suggested the low cytotoxicity of FU-BT-CDs in the normal cell line for better target specificity because of biotin conjugation. It was observed that EWCDs (egg white derived) could be easily excreted through zebrafish metabolism and showed ∼80% cell viability in the BEAS-2B (human bronchial epithelial) cell line.^132^ The cytotoxicity evaluation of two types of N-CDs (WBCDs and EBCDs, derived from bee pollen) exhibited the toxic effects of WBCDs to HeLa, HCT-116 (human colorectal carcinoma cells), and HepG2 (human hepatocellular liver carcinoma cells), but no obvious toxicity to the SHSY5Y (human neuroblastoma cells) and RAW264.7 (mouse monocyte/macrophage cells) cell lines. Furthermore, the EBCDs showed slight toxicity to all five cells.^172^ Besides, severe *in vitro* cytotoxicity was observed for cow milk-derived CDs on mouse hippocampus cells (HT22, cell viability <60%) even at the lowest concentration of 0.4 mg mL^−1^.^197^

The toxicity study of hamburger-, lamb-, chicken-, and chicken breast-derived doped CDs exhibited the more cytotoxic effect of CDs obtained at a higher temperature in comparison to that synthesized at a relatively lower temperature (Table S2†) due to their surface functional groups and large amount of carbon core *via* effective carbonization.^149,153,158,159^ Interestingly, the lamb-derived N,S-CDs obtained at a longer baking time (CD-3: 45 min and cell viability: 45.0%) had much higher cytotoxicity than that from the shorter baking times (CD-1: 15 min, cell viability: 95.0% and CD-2: 30 min, cell viability: 74.0%), which is again related to the degree of carbonization and surface passivation. Moreover, the adverse effects of CD-3 were also judged by the perturbation of energy, amino acid, purine, and lipid metabolism.^154^ Thus, the results suggest that a lower processing temperature/time for animal-based food stuff is favorable to human health.

Dose-dependent cytotoxicity has also been observed in some animal/human biomass-derived CDs.^154,177,201,208,220,243–245^ For example, the low cytotoxicity of cow manure-derived PBA-CDs at a concentration of 0.01 mg mL^−1^ to B16F10 (murine melanoma cells) and NIH3T3 (fibroblast cells) cells was explained based on the surface-confined negative charges present in the boronic groups. However, the PBA-CDs showed significant cytotoxicity at a higher concentration (0.1 mg mL^−1^, cell viability: 60–70%).^220^

Besides *in vitro* cytotoxicity, *in vivo* biocompatibility has also been demonstrated in animal/human biomass-derived CDs. For example, N-CDs (derived from human hair/pig skin)^209^ and FA@Gd-CDs (FA-conjugated Gd-CDs derived from crab shell)^178^ exhibited no adverse effect on phenotypic development, survival rate, and hatching rate in zebrafish embryos. Similarly, an oral/injected dose of doped/modified CDs derived from SF,^143^ pork,^164^ milk,^195^ and cow manure^220^ showed an insignificant toxicological effect on BALB/c mice. *In vivo* cytotoxic assessment of the doped CDs (shark cartilage derived) also showed good tolerance to zebrafish larvae even up to the maximum concentration of 0.1 mg mL^−1^.^232^

#### 7.2.2 *In vitro*/*in vivo* bioimaging

**Fig. 16:**
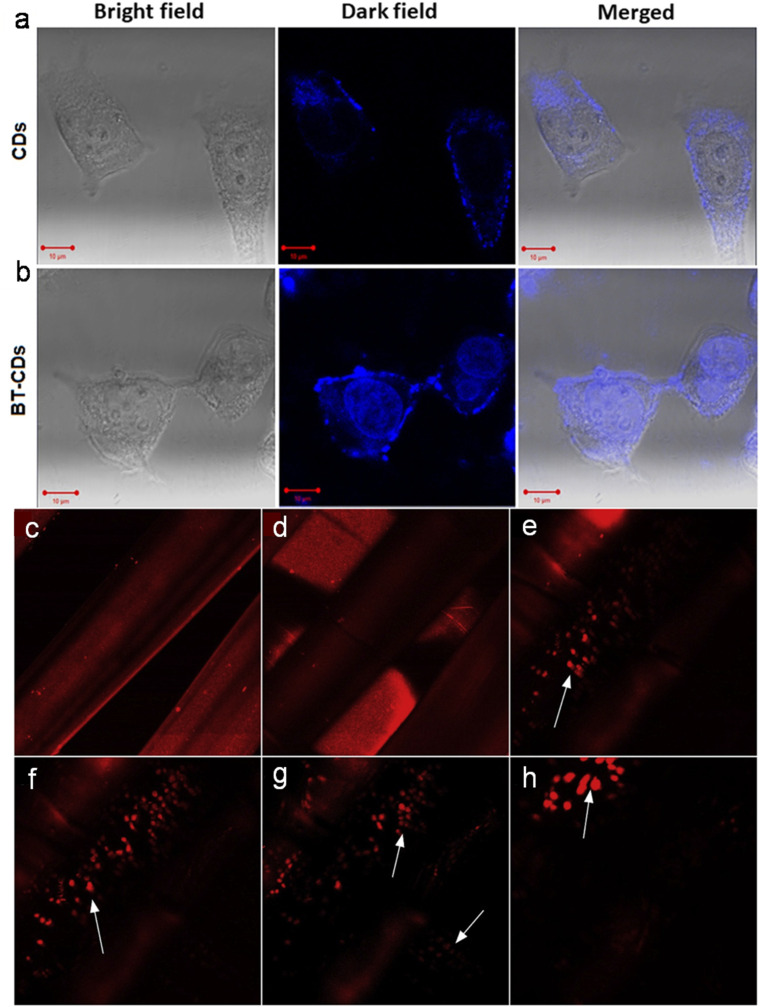
Confocal microscopy images showing the uptake of (a) CDs and (b) BT-CDs in MCF-7 cell lines as evidenced by bright blue fluorescence. Reprinted from ref. 147, Copyright (2021), with permission from Elsevier. The 3D-printed scaffold imaging of cell nuclei at variable depths: (c) outer layer, (d) *z* = 600 μm, (e) *z* = 800 μm, (f) *z* = 900 μm, (g) *z* = 1000 μm, and (h) *z* = 1500 μm. Reprinted from ref. 211, Copyright (2018), with permission from Elsevier.

*Bombyx mori* silk-derived N-CDs were the first example of animal/human biomass showing *in vitro* cellular uptake in A549 (human lung adenocarcinoma epithelial cells) cancerous cell nuclei.^138^ The possibilities of *in vitro*/*in vivo* bioimaging and cellular uptake *via* the endocytosis process using CDs derived from animal/human biomass are summarized in Table 3, which reveals that the internalization of CDs preferably occurs in the cytoplasm or cell interior/membrane instead of the nucleus. As evidenced by the intense blue fluorescence localized in the cytoplasm under UV irradiation, FA-conjugated N-CDs (derived from crab shell) were selectively targeted to the folate receptor over-expressing cancerous HeLa cells in comparison to the normal HEL (human erythroleukemia) cells.^179^ Two types of human urine-derived doped CDs (UCDs and HUCDs) were used to assess their *in vitro* distributions in the cytoplasm of HeLa and onion epidermal cells. Additionally, the *in vivo* imaging showed the location of UCDs/HUCDs in the pharynx, intestine, and anus regions of *Caenorhabditis elegans* (*C. elegans*) organism and interior of bean sprout plant tissue. Interestingly, in all the fluorescent labelling experiments, HUCDs exhibited brighter fluorescence than UCDs despite their large size, which is ascribed to their higher QY.^246^ The successful internalization of CDs (Fig. 16a) and BT-CDs (Fig. 16b) in MCF-7 cancer cells was evidenced by their bright blue fluorescence. It was observed that the CDs were only localized in the cytoplasm (Fig. 16a), while BT-CDs were distributed in the entire cell (Fig. 16b) – preferably in the nucleus due to receptor (BT)-mediated endocytosis. Moreover, the cellular uptake of BT-CDs was more specific towards cancer cells (MCF-7) in comparison to the normal HEK-293 cells due to the positive BT expression.^147^

**Table 3:** *In vitro* and *in vivo* cell imaging results of the animal/human biomass-derived CDs^a^^,^^b^

Source	Mode	Time (h)	Cell/plant/organism (amount of CDs in mg mL^−1^)	Uptake region/emission color	Ref.
Egg white	*In vitro*	0.5	HeLa^c^ (0.04)	Cytosol/green	129
Egg white (albumin)	*In vitro*	24	Bcap-37^c^ (2.0)	Cytoplasm/blue, green	130
Egg white	*In vitro*	02^a^	*E. coli* ^d^, *S. aureus*^d^ (0.1)	Entire bacteria/blue, green, red	131
Egg white	*In vivo*	24	Zebrafish^e^ (2.0)	Entire embryo/blue, green, red	132 ^i^
*Bombyx mori* silk	*In vitro*	24	A549^c^ (1.0)	Nucleus/blue, green, red	138
Silkworm chrysalis	*In vitro*	06	HeLa^c^ (6.0)	Cell interior/blue, green, red	140
Spider silk	*In vitro*	24	HepG2^c^ (0.5)	Cell interior/blue	142 ^i^
Silk fibroin	*In vitro*	04	KB^c^ (0.1)	Cytoplasm, nucleus/green	143
	*In vivo*	18	BALB/c Mice^e^ (50^f^)	Liver, lung/green	
Silk fibroin + LMWC	*In vitro*	24	MCF-7^c^ (0.1)	Cytoplasm (CDs)/blue; entire cell, nucleus (BT-CDs)/blue	147
Hamburger	*In vitro*	24	MC3T3-E1^c^ (0.5)	Cytoplasm/blue, green, red	149
	*In vivo*	05^b^	Mung bean sprout^g^ (3.2)	Root, stem, cotyledon, vascular system/blue	
Pike eel fish	*In vitro*	24	MC3T3-E1^c^ (1.5)	Cell membrane, cytoplasm, nucleus/blue, green, red	150
Pike eel fish	*In vitro*	24	MC3T3-E1^c^ (1.5)	Cytoplasm/blue, green, red	151
Lamb	*In vitro*	07	HepG2^c^ (1.0)	Cell membrane, cytoplasm/blue, green, red	153
Duck	*In vitro*	24	PC12^c^ (1.0)	Cytoplasm/blue	156
	*In vivo*	24	*C. elegans* ^e^ (15.0)	Intestine/blue	
Chicken	*In vitro*	24	HepG2^c^ (1.0)	Cytoplasm/blue	158
	*Ex vivo*	02	BALB/c Mice^e^ (2000^f^)	Brain/blue	
Chicken breast	*In vitro*	12	NRK	Lysosome	159
Chicken	*Ex vivo*	2–6	BALB/c Mice^e^ (2000^f^)	Stomach, intestine, testis, kidney	160
Pork	*In vivo*	0.5–6	BALB/c Mice^e^ (2000^f^)	Kidney, heart, brain, lung, intestine, testis, liver, stomach	164
	′′	24	*C. elegans* ^e^ (10.0)	Epithelium, digestive system/blue, green, red	
Honey	*In vitro*	01	HEp-2^c^, HeLa^c^ (1.0)	Cell interior/green	166
Rapeseed bee pollen	*In vitro*	12	LoVo^c^ (0.5)	Cytoplasm/blue, green, red	167
Bee pollen	*In vivo*	20^b^	*B. parachinensis* L.^g^ (3.5)	Periplasmic space/blue	168
Rapeseed bee pollen	*In vitro*	02	Lettuce^g^ (1.0)	Leaf vein, stem, root/blue	169
Bee pollen	*In vitro*	15^a^	HepG2^c^ (0.1)	Cytoplasm/blue	172
Shrimp egg	*In vitro*	24	SK-Hep-1^c^ (0.2)	Cell membrane, cytoplasm/blue, green, red	173
Dried shrimp	*In vitro*	3/48	MCF-7^c^, SH-SY5Y^c^ (0.1)	Cytoplasm/blue, green, red	177
Crab shell + GdCl_3_	*In vitro*	24	HeLa^c^, HepG2^c^ (0.5)	Cytoplasm/blue	178
Crab shell	*In vitro*	24	HeLa^c^ (0.5)	Cytoplasm/blue	179
Shrimp shell	*In vivo*	—	Nematode^e^ (—)	Entire body/green	184
Crayfish shell	*In vitro*	24	HeLa^c^ (0.55)	Cytoplasm/red	185 ^i^
Mussel seafood	*In vitro*	24	HepG2^c^ (1.5)	Cytoplasm/blue	188
	*In vitro*	10^a^	Onion epidermal cell^g^ (1.5)	Cell wall/blue	
	*In vivo*	48	Zebrafish^e^ (1.5)	Liver, yolk, intestine/blue	
Milk	*In vitro*	02	U87^c^ (0.01)	Cell interior/blue	189
Milk	*In vitro*	01	SMMC-7721^c^ (50^h^)	Cell interior/blue, green	190
Milk	*In vitro*	—	HeLa^c^ (—)	Cell membrane, cytoplasm/blue, green, red	193
	*In vivo*	—	Tumour bearing BALB/c Mice^e^ (0.5)	Tumour tissue	
Milk	*In vitro*	02	HeLa^c^ (0.2)	Cytoplasm/green	194
Pasteurized milk	*In vitro*	02	HeLa^c^ (0.01)	Lysosome/blue (CDs); Cytoplasm/blue (Lis-CDs)	201
Expired milk	*In vitro*	06	HeLa^c^ (0.2)	Cytoplasm/green	202
Denatured sour milk	*In vitro*	72	U-251 MG^c^ (0.05)	Cell membrane, cytoplasm/blue	204
Pigeon feather	*In vitro*	01	HUVEC^c^ (2.0)	Cell membrane, cytoplasm/blue	134 ^i^
Pig skin	*In vitro*	0.5	HeLa^c^ (0.35)	Cell interior/blue, green, red	208 ^i^
Pig skin	*In vivo*	20	Zebrafish^e^ (0.2–1.6)	Entire embryo/blue, green, red	209 ^i^
Pig skin collagen	*In vitro*	24	RL-14^c^ (0.5)	Cytoplasm/bright	211
GGEC	*In vitro*	04	Melanoma^c^ (1.0)	Cytoplasm/blue	216
Cow manure	*In vitro*	0.5	MCF-7^c^, HUVEC^c^, MDA-MB-231^c^, Caco-2^c^, DU-145^c^ (2.5)	Cytoplasm/green (unmodified CDs); Nucleolus/green (modified CDs)	219
Cow manure	*In vitro*	—	MCF-7^c^ (—)	Mitochondria/green, red	220
	*In vivo*	2–48	C57BL/6 Mice^e^ (0.16–0.32)	Entire body	
Fish scale (crucian carp)	*In vitro*	24	KG-1a^c^ (0.125)	Cell interior/blue	225 ^i^
Fish scale (*Lethrinus lentjan*)	*In vitro*	06	hMSCs^c^ (0.025)	Cytoplasm, nucleus/blue, green, red	226
Shark cartilage (chondroitin sulphate)	*In vivo*	24	Zebrafish^e^ (0.1)	Intestine/blue, green	232
Sheep wool	*In vitro*	0.5	HeLa^c^ (0.3)	Cytoplasm, nucleus/blue, green	235 ^i^
Hair	*In vitro*	04	HeLa^c^ (0.2)	Cell membrane, cytoplasm/blue	239
Hair	*In vivo*	20	Zebrafish^e^ (0.2–0.8)	Entire embryo/blue, green, red	209 ^i^
Urine	*In vitro*	10^a^	MEF^c^ (0.2)	Cytoplasm/green, red	244
Urine	*In vitro*	04	HeLa^c^ (1.0)	Cytoplasm/blue, green	246
	*In vivo*	24	*C. elegans* ^e^ (20)	Pharynx, intestine, anus/blue	
	*In vitro*	06	Onion epidermal^g^ (3.0)	Cytoplasm/blue	
	*In vivo*	24	Bean sprouts^g^ (20)	Tissue interior/blue	
Fingernail	*In vitro*	02	A549^c^, MDA-MB-231^c^, HeLa^c^ (0.2)	Cytoplasm/blue	247
Fingernail	*In vitro*	02	A549^c^, HeLa^c^, MDA-MB-231^c^, HEK-293^c^ (0.2)	Perinuclear region/blue	248

a ^a^Minutes, ^b^days, ^c^cells, ^d^bacteria, ^e^organism, ^f^mg kg^−1^ of mouse, ^g^plant, ^h^μM, and ^i^intracellular bioimaging of metal ions/other analytes.

b Bcap-37: human breast cancer cells, *S. aureus*: *Staphylococcus aureus*, KB: epithelial carcinoma cells, MC3T3-E1: mouse osteoblast cells, PC12: rat pheochromocytoma cells, NRK: normal rat kidney epithelial cells, HEp-2: human larynx carcinoma cells, LoVo: human colon carcinoma cells, *B. parachinensis*: *Brassica parachinensis*, SK-Hep-1: human hepatoma cell lines, SH-SY5Y: human neuroblastoma cells, U87: human brain glioma tumor cells, SMMC-7721: human liver cancer cells, U-251 MG: human malignant glioblastoma cell lines, HUVEC: human umbilical vein endothelial cells, MDA-MB-231: human breast cancer cells, Caco-2: human colorectal adenocarcinoma cells, DU-145: human prostate cancer cells, KG-1a: promyeloblast macrophage cells, hMSCs: human mesenchymal stem cells, and MEF: mouse embryonic fibroblast cells.

Distinct distributions of animal-derived CDs in living cells *via* two-photon excited fluorescence imaging (with longer wavelength femtosecond laser, 800/780 nm) were also noticed without obvious auto-fluorescence, which is advantageous for cell imaging without photon-induced damage.^194,202,211^ For example, Dehghani *et al.*^211^ performed two-photon imaging of RL-14 (human fetal ventricular cardiomyocytes) cells under a 780 nm pulse laser to confirm the uptake of N-CDs (pig skin collagen derived) inside the cytoplasm. Moreover, the 3D luminescent scaffolds with a tailored design could visualize the cell structures at various depths (*z* = 0 to 1500 μm) using two-photon microscopy (Fig. 16c–h).

**Fig. 17:**
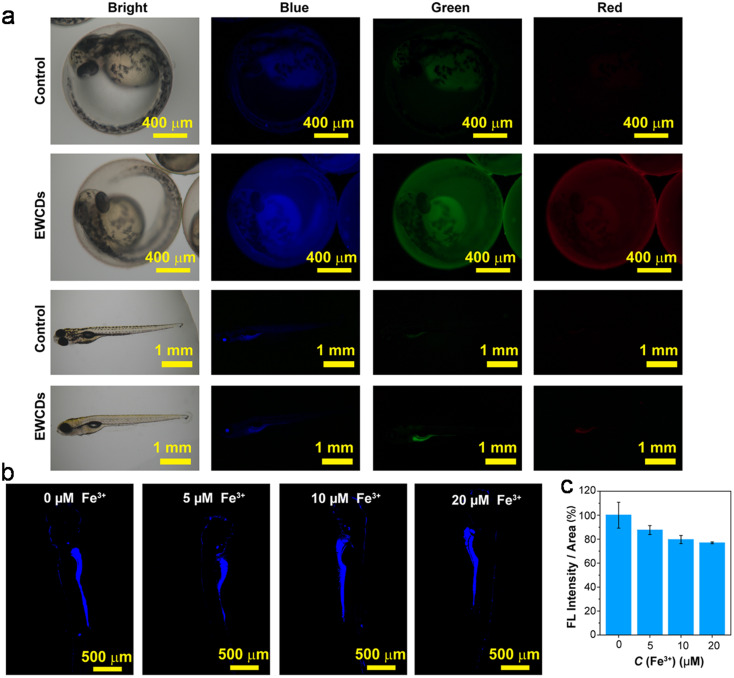
(a) The bright-field and fluorescent field (blue, green, and red) imaging of zebrafish treated with egg white-derived EWCDs, (b) zebrafish larva incubated with EWCDs and different concentrations of Fe^3+^, and (c) average fluorescence statistics from confocal laser scanning microscopy images. Reprinted (adapted) with permission from ref. 132, Copyright (2021), the American Chemical Society.

Zebrafish was used as a model organism to investigate the transport/distribution of animal-derived CDs *via in vivo* imaging (Table 3).^132,188,209,232^ For example, the *in vivo* imaging of EWCDs (derived from egg white) confirmed their internalization in zebrafish embryo *via* chorion as evidenced by the characteristic blue/green/red emission of 2 hpf (1 hpf = 24 h post-fertilization) zebrafish embryo and 5 dpf (1 dpf = 1 day post-fertilization) zebrafish larvae (Fig. 17a).^132^ N-CDs (derived from mussels) were localized outside the cell walls/cytoplasm when applied for *in vitro* bioimaging in onion epidermal/HepG2 cells, while *in vivo* blue fluorescent monitoring showed their presence in the liver, yolk, and intestine of zebrafish larva.^188^

**Fig. 18:**
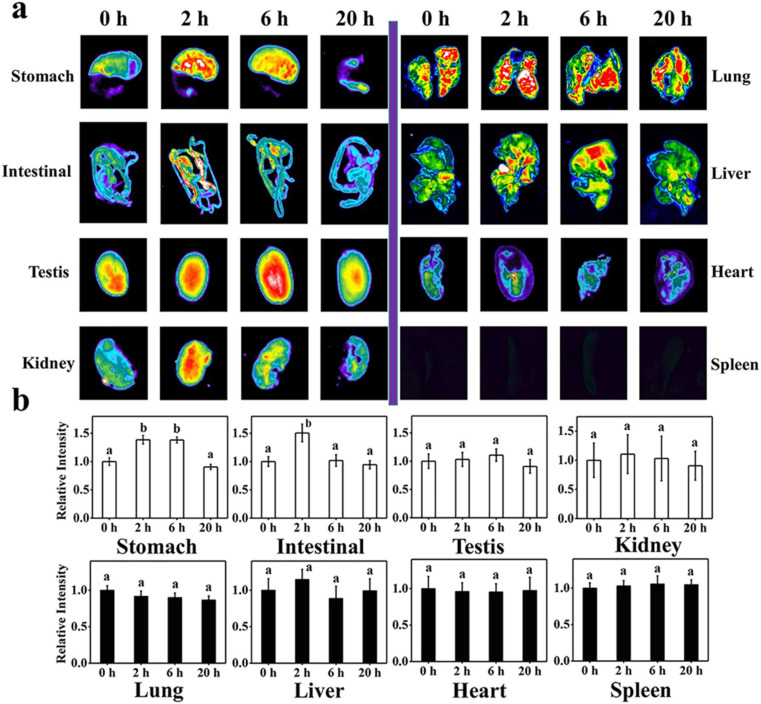
(a) Fluorescence images acquired from the different major organs of BALB/c mice immediately after the oral supplementation of chicken-derived N-CDs and after 2, 6, and 20 h. (b) Plots of relative fluorescence intensity of imaged organs with corresponding time (a and b letters in (b) represent the major difference in the relative intensity at *P* < 0.05). Reprinted from ref. 160, Copyright (2022), with permission from Elsevier.

Animal-derived doped/modified CDs were also supplemented in mice to access their fluorescence imaging/spectral contrast-based distributions (Table 3).^143,158,160,164,193,220^ For example, the *ex vivo* oral administration of N-CDs (chicken derived) in BALB/c mice suggested their maximum appearance in the digestive system including stomach and intestine (2 h), followed by testis and kidney (2–6 h), while a minimal effect on the lung, liver, heart, and spleen (Fig. 18a). The subsequent reduction of the internalized N-CDs in the digestive system after 20 h metabolic process could be seen by the change in fluorescence intensity (Fig. 18a and b). Moreover, the *in vitro* studies demonstrated several adverse effects of N-CDs on the digestibility of SPI (soy protein isolate), digestion product (polypeptides and free amino acids), and pepsin activity, which suggested their unfavorable interaction with digestive enzymes.^160^

Besides *in vivo*/*in vitro* bioimaging, animal biomass-derived CDs are also consumed for the intracellular sensing of metal ions^132,134,142,208,209,225,235^ or other analytes (TAP)^185^ through suppressed fluorescence signaling. For example, the excellent *in vitro* selectivity of egg white-derived EWCDs with Fe^3+^ (Table 2) was further implemented for the *in vivo* detection of Fe^3+^ in zebrafish. A gradual decrease in surface-based fluorescence intensity with an increase in Fe^3+^ concentration can be seen in Fig. 17b and c, suggesting the permeation of EWCDs and absorption of Fe^3+^ in zebrafish embryos.^132^ Similarly, the intracellular monitoring of TAP in HeLa cells showed a significant decrease in red fluorescence (originated from N,S-CDs) with an increase in TAP concentration.^185^

### 7.3 Other applications

The promotion of plant growth using animal biomass-derived CDs is another growing area of interest.^168,169,172^ For instance, bee pollen-derived EBCDs exhibited better promotion capability on the growth of the classical *Arabidopsis* plant model in comparison to WBCDs. The high growth-promoting ability of EBCDs was ascribed to their high content of N- and O-heteroatoms, which resulted in greater biocompatibility and bioactivity.^172^

The antimicrobial activity of animal-derived CDs has also been investigated.^171,181,184^ For example, crab shell-derived CDs were used as an antibacterial agent against highly noxious human pathogenic bacteria (*Escherichia coli* (*E. coli*) and *Klebsiella pneumonia*). The high inhibition activity of CDs in this study was ascribed to the effective damage of the cell membrane *via* the generation of ROS on the surface of CDs.^181^

Animal biomass-derived CDs have also been investigated for therapeutic application.^132,220^ For example, boronic acid-functionalized PBA-CDs (derived from cow manure) were employed as an immunotherapy agent to treat melanoma skin cancer-bearing mice, which exhibited interaction with tumour tissues and stimulated the immune system against the disease. The histopathological imaging of the cancer-bearing mice after treatment with PBA-CDs exhibited no obvious damage to the brain and kidney tissues, while the liver, spleen, and lung tissues showed inflammatory focus, white pulp hyperplasis, and fibrosis/inflammatory infiltrate, respectively. The histological analyses also revealed necrosis and inflammatory infiltrate on large areas of tumour tissues after PBA-CD treatment.^220^

The drug delivery applications of doped CDs derived from dried shrimps,^177^ crab shell,^178^ and pasteurized milk^201^ are also appreciable. For instance, magneto-fluorescent Gd-CDs (derived from a mixture of crab shell and GdCl_3_) showed potential for diagnostic and theranostic application. FA@Gd-CDs specifically targeted HeLa cells (folate receptor-positive) *via* folate receptor-mediated endocytosis. Moreover, doxorubicin (DOX, anti-cancer drug) conjugated with FA@Gd-CDs (∼74.5% loading) exhibited higher release kinetics at low pH (5.0) in comparison to high pH (7.4), which was ascribed to the weakened DOX binding with CDs due to the protonation of the –NH_2_ groups present in DOX.^178^

Animal-derived CDs have also been employed to prepare fluorescent ink for multi-colour coating, patterning, and word writing, which is vital for staining, information encryption, and anti-counterfeiting applications.^126,145,166,193,202,204,221,235^ Li *et al.*^145^ employed silk sericin-derived N-CDs to prepare multimodal quick response/bar security codes and Morse code-based encryption, which could be recognized under UV irradiation and immediately after switching-off of the UV light. Moreover, the N-CD ink coated on different fabrics (silk, cotton, and terylene) was visible once the UV light is turned-off after exposure.

The catalytic and photocatalytic applications of animal/human biomass-derived CDs have also been investigated.^148,167,206,228,249^ Recently, Din *et al.*^206^ demonstrated 92% degradation of methylene blue (MB) by chicken feather-derived CDs during 60 min exposure to sunlight. The catalyst also showed reusability of up to 5 cycles. The photo-generated electrons/holes on the surface of the CDs induced the formation of superoxide anion radical (O_2_˙^−^)/˙OH, which eventually decomposed the aromatic structure of the dye molecules.

Athika *et al.*^203^ investigated denatured milk-derived CDs for supercapacitor application. The rectangular shape of the cyclic voltammetry curves and almost symmetrical galvanostatic charge-discharge (GCD) profiles implied the existence of double layer capacitance. The specific capacitance was estimated to be 95.0 F g^−1^ together with 100% columbic efficiency/cyclic stability during 1000 GCD cycles at 0.12 A g^−1^.

The free radical scavenging activity of animal source-derived CDs is also remarkable.^153,161^ Mackerel fish-derived N-CDs were utilized for unique free radical (˙OH and ˙CH_3_) scavenging activity, which showed potential to protect against oxidative stress under biological condition. The scavenging of ˙OH radicals *via* Fenton reaction resulted in strong fluorescence quenching due to the changes in the energy levels of the N-CDs. The dose-dependent scavenging exhibited 90% disappearance of the ˙OH signal using 15.0 mg mL^−1^ N-CDs. Additionally, the 41.4% decrease in the reaction rate constant implied the remarkable reduction of the MB (source of ˙CH_3_ generation by visible-light photosensitization) degradation rate *via* ˙CH_3_ scavenging.^161^

The non-linear optical (NLO) phenomenon refers to a process that non-linearly depends on the excitation light parameters. An aqueous dispersion of N,S-codoped CDs (obtained from a mixture of honey, garlic extract, and NH_3_) showed a self-defocusing and strong NLO response. The third-order NLO susceptibility was estimated to be significantly high (2.29 × 10^−6^ esu) together with reverse saturable absorption in the open aperture curve, which indicated the suitability of doped CDs for NLO application.^171^

## 8 Challenges and future prospects

Considering the significant utilization of animal/human biomass in the synthesis and potential applications of sustainable CDs, there are several challenges and prospects that require attention in the foreseeable future, as follows:

(1) Exploring novel precursors capable of self-doping and surface passivation, coupled with refined bottom-up synthetic protocols can potentially enhance both the performance and fluorescent QY of CDs.

(2) Attaining precise control of the size distribution, degree of graphitization, and surface functionalization remains crucial for achieving exceptional optical properties in CDs, necessitating further improvement.

(3) The presence of impurities in CDs has the potential to impede the desired performance outcomes. Therefore, developing new, uncomplicated, and cost-effective purification protocols is essential to achieve optimal results.

(4) Although the applications of animal/human biomass-derived CDs have shown promise at the laboratory scale, achieving their commercial-scale production has proven to be a challenge. Thus, comprehensive research is needed to enable the large-scale synthesis of CDs from these readily available precursors while retaining their inherent small-scale properties.

(5) Despite the clear influence of synthetic parameters and starting sources, the actual underlying growth mechanism of these CDs remains unknown, requiring comprehensive understanding through empirical/*in situ* experimental evidence.

(6) The precise origins of the PL and EWD/EWID features in these CDs still need to be elucidated, making it a pivotal area for in-depth investigation.

(7) In addition to the common down-conversion fluorescence, some animal/human biomass-derived CDs exhibit UCPL (Table S1†), which holds potential for near infrared (NIR) cellular imaging *via* two-photon luminescence and catalyst fabrication for energy-related applications. Consequently, these application areas offer fertile ground for new research.

(8) The design of CDs with long *λ*_em_ using animal/human biomass remains uncharted. Therefore, dedicated efforts should be devoted to creating CDs with NIR absorption and emission properties, leveraging the low biotoxicity and efficient tissue penetration capabilities of NIR light.

(9) Many sensors for metal ions and their tracking in biological systems using these CDs rely on light-down (fluorescence quenching) mechanisms. However, the development of light-up (fluorescence enhancement, Table 2) phenomena in the presence of analytes can be a valuable advancement, addressing the challenges posed by the high background fluorescence in biological environments.

(10) Opportunities exist to enhance the sensitivity and selectivity of analyte detection and tracking in real samples and biological systems by producing CD nanoprobe sensors using new precursors or modifying chemical composition/structure. Furthermore, there is untapped potential to develop sensing platforms for various toxic metal ions such as Mn^2+^, Pb^2+^, As^3+^, Po^3+^, and other unexplored analytes using these CDs in the future.

(11) Investigation of the long-term acute toxicity of animal/human-derived CDs on vertebrates and humans is insufficient. Moreover, advancement in the *in situ* tracking of these CDs may facilitate the in depth understanding and assessment of the metabolic process.

## 9 Conclusions

This review article presented a comprehensive overview of CDs synthesized from biomass of animal and human origin, aiming to provide researchers with insights for novel experimental implications and advancements in this field. This type of biomass, which is often considered inexpensive or waste materials, offers a sustainable and eco-friendly resource that can be converted into value-added products. The choice of precursor and synthetic method significantly influences the fundamental structure, heteroatom doping, and surface functional groups of the resulting CDs. Consequently, both the CDs and their doped counterparts can benefit from size control, leading to an enhanced QY. Many of these CDs exhibit self-passivation through various functional groups, offering opportunities for tailored modifications catering to specific applications. Notably, the optical properties of these CDs are closely related to their structure and chemical composition. In addition to the common emission known as EWD fluorescence, these CDs display EWID and UCPL behaviours. Their EWD fluorescence properties are primarily influenced by the quantum size effect of the core structure, the presence of surface emission centers arising from the presence of functional groups or defect sites, and the radiative recombination of excitons trapped at the surface. Conversely, their UCPL characteristics stem from a multi-photon activation process. The outstanding PL properties of these CDs combined with their attributes such as photo-stability, low cytotoxicity, and high biocompatibility make them promising candidates for various sensing, bioimaging, and many more applications. Consequently, the utilization of animal and human biomass has potential to emerge as a sustainable and eco-friendly platform for creating fluorescent CDs characterized by excellent optical properties, thereby opening diverse avenues for their application.

## Conflicts of interest

There are no conflicts to declare for financial interests or personal relationships.

## Supplementary Material

RA-013-D3RA06976A-s001
