# Supplementary material for: An overview on animal/human biomass-derived carbon dots for optical sensing and bioimaging applications
Source: RSC Adv. 2023 Dec 1;13(50):35088–126. doi: 10.1039/d3ra06976a (PMC10690874; doi:10.1039/d3ra06976a)
Supplement: RA-013-D3RA06976A-s001 [file RA-013-D3RA06976A-s001.pdf]

Electronic Supplementary Material (ESI) for RSC Advances.
This journal is © The Royal Society of Chemistry 2023

An overview on animal/human biomass-derived carbon dots for optical sensing and bioimaging applications

Prashant Dubey

Centre of Material Sciences, Institute of Interdisciplinary Studies (IIDS), University of Allahabad, Prayagraj-211002, Uttar Pradesh (INDIA)

Email: pdubey@allduniv.ac.in, pdubey.au@gmail.com

Table S1 Optical results of CDs obtained from various animal/human biomasses.

**Source**	**λ_{abs} (nm)**		**λ_{ex} (nm)**	**λ_{em} (nm)**	**Color in UV light**	**Nature of PL**	**Origin of PL**	**τ_{av} (ns)$^{decay\ behavior}$**	**Ref.**
	**$\pi\rightarrow\pi^*$**	**$n\rightarrow\pi^*$ or surface states**							
			**Animal biomass-derived CDs**						
Egg white/yolk	275		360	420	B	EWD	(ii)	6.37^2	[126]
Egg shell membrane	Featureless		275	450	B	—	—	—	[127]
Egg shell membrane	220	320	350	420	B	EWD	(ii)	6.36^2	[128]
Egg white	260–290		315	420	B	EWD	(ii)	9.1	[129]
Egg white (albumin)	218	276	333	412	—	EWD	(i), (ii)	$7.2–8.7^2$	[130]
Egg white	Featureless		300	395	B	EWD	(i), (ii)	6.5	[131]
Egg white	270	—	340	420	B	EWD	—	—	[132]
Egg white	204, 270	> 300	360	441	B	EWD	(ii)	—	[133]
Pigeon egg white	—	250–300	340	400	B	EWD	(ii)	—	[134]
Pigeon egg yolk	220–240	—	340	400	B	EWD	(ii)	—	
Egg yolk oil	270	—	200–400	420	B	EWID	—	—	[135]
Egg yolk	—	290	330	~ 400	B	EWD	(ii)	2.5^2	[136]
Egg yolk	275		332	408	B	EWD	—	—	[137]
Bombyx mori silk	278	—	370<br>780	450<br>412	B-G	EWD<br>UC	(ii)	4.23^3	[138]
Cocoon silk + H_2O_2	—	268, 320	370	438	B	EWD	(ii)	3.99^2	[139]
Silkworm chrysalis	276	317	350	420	B	EWD	(ii)	—	[140]
Bombyx mori silk + CA	267	314	360	425	B	EWD	(i), (ii)	—	[141]
Spider silk	272	333	370	452	B	EWD	—	6.43^3	[142]
Silk fibroin	272	320	340	~ 420	B	EWD	—	—	[143]
Silk fibroin	275	330	330<br>900	420<br>~ 550	B	EWD<br>UC	(i)	1.94^2	[144]
Silk sericin	215, 245, 286,	320	240–340	~ 420	B	EWID	(i)	5.62^2	[145]
Silk sericin	215	281	350	420	B	EWD	(i), (ii)	—	[146]
Silk fibroin + LMWC	275	320–350	350	~ 420	B	EWD	(i)	—	[147]
Barbeque beef	Featureless		440	520	—	EWD	(ii)	—	[148]
Hamburger	200–250	250–350	320	380	B	EWD	(ii)	6.49^1	[149]
Pike eel fish	—	335	410	475	S	EWD	(ii)	6.89^2	[150]
Pike eel fish	278	335	405	465	C	EWD	(i), (ii)	7.17^2	[151]
Lamb	—	260	370	426	B	EWD	(ii)	8.05^2	[152]
Lamb	240	325	330	385	B	EWD	(ii)	9.1^2	[153]
Lamb	200–250	320	400	455	B	EWD	(ii)	7.96^3	[154]

Duck	239	—	360	435	B	EWD	(ii)	10.47[2]	[155]
Duck	239	—	370	~ 435	B	EWD	(ii)	7.65[2]	[156]
Chicken breast	250	—	320	385	B	EWD	(ii)	4.8[2]	[157]
Chicken	—	350	375	460	B	EWD	(i), (ii)	10.57[2]	[158]
Chicken breast	—	—	—	—	—	—	—	—	[159]
Chicken	245–255		370	440	—	EWD	(ii)	7.93[2]	[160]
Mackerel fish	278	329	380	451	B	EWD	(ii)	9.1[2]	[161]
Mackerel fish	~ 220	—	—	—	B	—	—	7.719	[162]
Salmon fish	241	—	—	—	B	—	—	6.43	[163]
Pork	~ 260	~ 320	380	460	C	EWD	(ii)	9.53[2]	[164]
Beef	Featureless		360	~ 460	—	EWD	—	8.78[2]	[165]
Honey	278		338	420	B	EWD	—	—	[166]
			700	435		UC			
Rapeseed bee pollen	280	—	360	435	B-G	EWD	(ii)	7.28[2]	[167]
Camellia bee pollen	″				″	EWD		7.54[2]	
Lotus bee pollen	″				″	EWD		7.61[2]	
Bee pollen	—	—	340	430	B	EWD	(ii)	—	[168]
Rapeseed bee pollen	270	—	360	432	B	EWD	(i)	—	[169]
Honey	280	—	360	456	B-G	EWD	—	4.67[2]	[170]
Honey + Garlic extract + NH_3	271	315	330	424	B	EWD	(i), (ii)	—	[171]
Bee pollen (water)	~275		365	~ 420	B	EWD	(i), (ii)	2.97	[172]
Bee pollen (ethanol)	~275		365	~ 420	B	EWD	(i), (ii)	2.92	
Shrimp egg	295	345	320	420	B	EWD	—	—	[173]
Prawn shell	280	330	330	405	B	EWD	(ii)	—	[174]
Prawn shell	265, 345	—	345	417	B	—	—	—	[175]
Prawn shell + Urea	275	335	355	415	B	EWD	(ii)	—	[176]
Dried shrimp	321		430	475	B	EWD	(i), (ii)	7.53[3]	[177]
Crab shell + $GdCl_3$	235, 275	350	350	425	B	EWD	(i), (ii)	—	[178]
Crab shell	—	260	380	~ 450	B	EWD	(i), (ii)	—	[179]
Crab shell	240	340	340	412	—	EWD	(i), (ii)	—	[180]
Crab shell	265	~ 330	330	420	G	EWD	(i), (ii)	—	[181]
Shrimp shell	—	—	—	—	—	—	—	—	[182]
Shrimp shell	—	—	350	~ 400	—	EWD	(ii)	—	[183]
Shrimp shell	265		355	450	G	EWD	—	—	[184]
Crayfish shell	265		372	446	B	EWID	—	1.72	[185]
Crayfish shell	273	364	360	440	B	EWD	(i), (ii)	8.38	[186]
Crayfish shell	273	—	350	430	B	EWD	(i), (ii)	—	[187]
Mussel seafood	260	—	370	437.5	B	EWD	(i), (ii)	7.07[2]	[188]
Milk	280	—	360	454	B-G	EWD	(ii)	1.61[3]	[189]
Milk	275	—	380	466	B-G	EWD	(i), (ii)	5.54–6.07[3]	[190]
			850	506		UC			
Milk + L-cysteine	274	310	340	430	B-G	EWD	″	5.07–5.47[3]	
			850	506		UC			
Milk + urea	274	310	320	435	B-G	EWD	″	5.20–5.85[3]	
			760	456		UC			
Cow milk	274		379	448	B	EWD	(ii)	8.9[3]	[191]
Cow milk	274		—	—	—	—	—	—	[192]
Milk	275		400	480	B	EWD	(ii)	6.88–7.20[3]	[193]
Milk	—	—	365	450	B	EWD	—	5.06	[194]
Milk	240	400, 450	370	450	C	EWD	(ii)	—	[195]

Milk powder + $FeCl_3$	—	281	370	460	B	EWD	(ii)	—	[196]
Cow milk	280±3		300	~ 420	—	—	—	—	[197]
Cow milk	275	330	475	550	—	EWD	(i), (ii)	—	[198]
Cow milk	275	—	360	440	B	EWD	—	—	[199]
Milk powder + Methionine	230	315	360–410	~ 470	B	EWID	—	12.78[2]	[200]
Pasteurized milk	245	302	370	455	B	EWD	(i), (ii)	4.89[2]	[201]
Expired milk	280	—	360	440	B	EWD	(ii)	—	[202]
Denatured milk	290	—	350	425	B	EWD	(i), (ii)	7.92[2]	[203]
Denatured sour milk	290	300–400	350	430	B	EWD	(i), (ii)	—	[204]
Goose feather	270	—	340	~ 420	B	EWD	(ii)	—	[205]
Pigeon feather	Featureless		330	400	B	EWD	(ii)	—	[134]
Chicken feather	215	275	—	—	—	—	—	—	[206]
Carbonized hen feather + Zn salt	—	—	316	390	—	EWD	(i), (ii)	2.199[3]	[207]
Carbonized hen feather + Mg salt			320	412			″	1.839[3]	
Pig skin	260	293	460	521	G	EWD	—	8.89[2]	[208]
Pig skin	Featureless		320	380	B	EWD	(i), (ii)	—	[209]
Pork	235	281	310	412	B	EWD	—	—	[210]
Pig skin collagen	265	320	350	435	B	EWD	(i), (ii)	3.86[2]	[211]
			780	510		UC			
Pork rib bone	259	—	300–400	453	—	EWID	(i)	3.86	[212]
Pork liver	263	—	329	410	B	EWD	—	4.53	[213]
Chicken drumstick	—	280	330	417	B	EWD	(i), (ii)	—	[214]
Bovine bone	259	—	310–400	453	—	EWID	(i)	5.272	[215]
Pork bone	259	—	″	″		″		2.653	
Sheep bone	259	—	″	″		″		2.882	
GGEC	—	273	350	420	B	EWD	(ii)	12.9	[216]
Chicken cartilage	252	330	360	435	—	EWD	(i), (ii)	9.76[2]	[217]
Chicken bone	—	—	380	465	B	EWD	(ii)	—	[218]
Cow manure	—	—	360	~ 450	B	EWD	(ii)	—	[219]
Cow manure	—	284	360	475	—	EWD	(i), (ii)	—	[220]
Pigeon manure	225	285	350	400	B	EWD	(ii)	—	[134]
Pigeon manure	230, 289	332	334	~ 470	B	EWD	(ii)	—	[221]
Fish scale (grass carp)	266	316	360	430	V-B	EWD	(i), (ii)	—	[222]
Fish scale (grass carp)	270	300–400	200–290	420	B	EWID		4.43[1]	[223]
			360	440		EWD	(i), (ii)		
			700	440		UC			
Fish scale (grass carp)	220	287	355	441	B	EWD	—	—	[224]
			800	415		UC			
Fish scale (crucian carp)	263	300–350	220–290	425	B	EWID		6.2[2]	[225]
			350	440		EWD	(i), (ii)		
			700	440		UC			
Fish scale (*Lethrinus lentjan*)	—	280	340	~ 420	B	EWD	(i), (ii)	—	[226]
Fish scale (*Ctenopharyngodon idella*)	270		250–405	335, 460	B	EWID	(iii)	—	[227]
Fish scale (*Dicentrarchus labrax*)	250	350	365	450	—	EWD	(ii)	6.98[3]	[228]
Fish scale (*Dicentrarchus labrax*)	—	—	—	—	—	—	—	—	[229]
Fish scale (*Labeo rohita*)	216	263	360	434	B-G	EWD	(i), (ii)	—	[230]
Fish scale (silver carp)-HT	250–290	—	360	430	B	EWD	(i), (ii)	—	[231]

Fish scale (silver carp)-MW	″		280–350	400	″	EWID	(ii)	—	
Shark cartilage (chondroitin sulphate)	287	324	390	~ 475	B	EWD	(ii)	—	[232]
Carp roe	—	255	365	443	B	EWD	—	3.56	[233]
Fish scale collagen peptides-HT	< 300	—	350	420	B	EWD	(i), (ii)	—	[234]
Fish scale collagen peptides-MW	″		360	430	″	″	″	—	
Sheep wool	282	310	340	402	B	EWD	—	6.8491[2]	[235]
Sheep wool	270	—	360	460	B	EWD	(i), (ii)	7.86[3]	[236]
Sheep wool	272	340	350	430	—	EWD	—	—	[237]
Wool keratin	270	323	365	434	B	EWD	(i), (ii)	9.90[3]	[238]
			**Human biomass-derived CDs**						
Hair fiber	323		330	383	P-B	EWD	(ii)	4.72[2]	[239]
			655	~ 383		UC			
	300		353	450	B	EWD	″	5.34[2]	
			726	~ 450		UC			
	295		369	470	G-B	EWD	″	5.17[2]	
			731	~ 470		UC			
Hair	270	330	360	430	B	EWD	(i), (ii)	2.83[2]	[240]
Hair	275	330	330	415	B	EWD	—	5.15	[241]
Hair	Featureless		320	380	B	EWD	(i), (ii)	—	[209]
Hair-AT	280	—	435	400–500	B	—	(ii)	8.21[3]	[242]
Hair-MW	230	—	435	400–500	B	—	″	5.99[3]	
Hair	285 (n→σ*)	330	380	450	B	EWD	—	2.19	[243]
Urine (unmodified diet)	330, 390		325	392	—	EWD	—	—	[244]
Urine (vitamin C supplemented)	330, 385		350	427	—	EWD	—	—	
Urine (asparagus rich diet)	325, 400		425	500	—	EWD	—	—	
Urine + Citron fruit juice	—	333	355	~ 440	B	EWD	(i), (ii)	7.7[3]	[245]
Urine-GC	—	275, 325	400	~ 450	B	EWD	(ii)	6.83[3]	[246]
Urine-HT	—	232, 290	380	~ 450	B	EWD	″	7.60[3]	
Fingernail	—	300	320	380	B	EWD	(ii)	—	[247]
Fingernail	275	330	330/ 370	380/ 450	B	EWD	(ii)	—	[248]
Fingernail	—	301, 371	360	~ 470	B-G	EWD	(i), (ii)	—	[249]

Note: B: blue, B-G: bluish-green, S: sapphire, C: cyan, G: green, V-B: violet-blue, P-B: purplish-blue, G-B: greenish-blue, EWD: excitation wavelength dependent, UC: up-conversion, EWID: excitation wavelength independent, [1]: mono-exponential fitting, [2]: bi-exponential fitting, [3]: tri-exponential fitting.

Table S2 Cytotoxicological assessment of CDs derived from various animal/human biomasses under different cell lines.

**Source**	**Cell line**	**Assay**	**Amount of CDs (mg mL^{-1})**	**Time (h)**	**Cell viability (%)**	**Ref.**
Egg white	HeLa, KB	MTT	5–80	24	80–100	[129]
Egg white (albumin)	Bcap-37	MTT	0.4–2.0	24	98–94	[130]
Egg white	BEAS-2B	MTT	1.5	24	~ 80	[132]
Bombyx mori silk	A549	CCK-8	1.0	24	> 100	[138]
Silkworm chrysalis	HeLa	MTT	15.0	20	> 80	[140]

Spider silk	HepG2	MTT	1.0	24	88	[142]
Silk fibroin	KB	MTT	1.0	24	> 95	[143]
Silk fibroin + LMWC	MCF-7, HeLa, HEK-293	MTT	0.3	48	> 100, ~ 80, > 100	[147]
Hamburger	MC3T3-E1	MTT	3.2	24	90 (220 °C), 85 (260 °C), 79 (300 °C)	[149]
Pike eel fish	MC3T3-E1	MTT	20.0	24	96.5	[150]
Pike eel fish	MC3T3-E1	MTT	20.0	24	~ 100	[151]
Lamb	HepG2	Annexin V-FITC/PI	2.0	4	93.3 (200 °C), 94.8 (300 °C), 92.1 (350 °C)	[153]
Lamb	PC-12	Annexin V-FITC/PI	4.0	24	95.0 (CD-1), 74.0 (CD-2), 45.0 (CD-3)	[154]
Duck	PC-12	MTT	1.0	36	91.2	[156]
Chicken	HepG2	MTT	4.0	24	85 (200 °C), 63 (250 °C), 67 (300 °C)	[158]
Chicken breast	NRK	Annexin V-FITC/PI	1.0	12	74.8 (200 °C), 48.8 (300 °C)	[159]
Pork	BALB/c Mice/*C. elegans*	Oral	2000[a]/ 10	24/ 96	No acute toxicity/toxicity	[164]
Honey	Seeds (soya bean, spanish)	External	10	1–8[b]	Non-toxic	[166]
Bee pollens	A549, HepG2	TE	0.5	24	> 100[c] (both cells)	[167]
Bee pollen (water)	SHSY5Y, RAW264.7, HeLa, HCT-116, HepG2	MTT	0.4	24	> 100 (first two cells), < 85 (last three cells)	[172]
Bee pollen (ethanol)	″	″	″	″	< 90 (all cells)	
Shrimp egg	SK-Hep-1	MTT	0.2	24	> 97	[173]
Dried shrimp	MCF-7	MTT	0.01/ 0.5	24/ 24	104/ 6.0	[177]
Crab shell + $GdCl_3$	HeLung, HeLa, HepG2	MTT	1.0	24	> 90 (all cells)	[178]
Crab shell	HeLa	MTT	1.0	24	> 90	[179]
Crayfish shell	HeLa	MTT	0.55	24	91.76	[185]
Mussel seafood	HepG2	MTT	6.0	24	> 100	[188]
Milk	U87	CCK-8	1.0	24	> 100	[189]
Milk	U87 MG	MTT	0.3	72	> 90	[193]
Milk	HeLa	MTT	0.2	24	> 95	[194]
Milk	BALB/c Mice	Oral	200[a]	14[b]	No acute toxicity	[195]
Cow milk	HT22	Invitrogen	0.4–40	24	< 60	[197]
Pasteurized milk	HeLa	MTT	0.1	24	97.3–95.1 (CDs), 93 (Lis-CDs)	[201]
Expired milk	HeLa/BMSCs P2	CCK-8	0.3/ 0.4	6/ 48	> 95 (both cells)	[202]
Denatured sour milk	U-251 MG	CCK-8	1.0	72	85	[204]
Pigeon feather	HUVEC	MTT	0.2	24	96	[134]
Pig skin	HeLa	MTT	0.1/ 0.8	24/ 24	100/ 82	[208]
Pig skin collagen	RL-14	TC10	0.5	24	> 95.0	[211]
GGEC	Melanoma	MTT	1.0	24	> 90	[216]
Chicken cartilage	HCT-116, MC, Hepa 1-6	CCK-8	0.2	24	> 80 (all cells)	[217]
Chicken bone	Hepa 1-6, HCT-116, MEFs	CCK-8	0.2	48	~ 100 (all cells)	[218]
Cow manure	MCF-7, HUVEC, MDA-MB-231, Caco-2, DU-145	MTT	2.5	24	> 95 (all cells)	[219]
Cow manure	B16F10, NIH3T3	MTT	0.1	24	60–70 (both cells)	[220]
Fish scale (grass carp)	PC-12	MTT	0.4	24	> 90	[223]
Fish scale (grass carp)	BV2	MTT	100	24	~ 96	[224]
Fish scale (crucian carp)	PC-12	MTT	0.5	24	~ 99	[225]
Fish scale (*Lethrinus lentjan*)	hMSCs	MTT	0.2	24/ 48	~ 93 (both times)	[226]

Carp roe	HCT-116, MC	CCK-8	0.2	60	> 90 (both cells)	[233]
Sheep wool	HeLa	MTT	0.8	24	> 86	[235]
Hair	HeLa	MTT	0.2	48	~ 84	[239]
Hair	HepG2	MTT	0.4	24	> 85	[243]
Urine	BT-474	SRB	0.5	48	> 90	[244]
Urine + Citron juice	HeLa	MTT	0.1/ 1.0	24	92/ 82	[245]
Urine	NRK	HP	0–0.5	12	0.15–0.6[d] (UCDs), 0.15–0.33[d] (HUCDs)	[246]
Fingernail	HeLa, A549, MDA-MB-231, HEK-293	CV	0.2	48	~ 98 (all cells)	[247]
Fingernail	A549, HeLa, MDA-MB-231, HEK-293	CV	0.2	24/ 48	> 100 (all cells, both times)	[248]

Note: HeLa: human cervical cancer cell lines, KB: human epithelial carcinoma cell lines, Bcap-37: human breast cancer cell lines, BEAS-2B: human bronchial epithelial cell lines, A549: human lung adenocarcinoma epithelial cancer cell lines, HepG2: human hepatocellular liver carcinoma cell lines, MCF-7: human breast cancer cell lines, HEK-293: human embryonic kidney cell lines, MC3T3-E1: mouse osteoblast cell lines, PC-12: rat pheochromocytoma cell lines, NRK: normal rat kidney cells, SHSY5Y: human neuroblastoma cell lines, RAW264.7: mouse monocyte/ macrophage cell lines, HCT-116: human colorectal carcinoma cell lines, SK-Hep-1: human hepatic adenocarcinoma cell lines, HeLung: human embryo lung normal cells, U87/ U-87 MG/ U-251 MG: human brain glioblastoma cells, HT22: mouse hippocampus cell lines, BMSCs P2: mouse bone marrow mesenchymal stem cells, HUVEC: human umbilical vein endothelial cell lines, RL-14: human fetal ventricular cardiomyocytes cell lines, MC: mouse cardiomyocyte cells, Hepa 1-6: murine hepatoma cell lines, MEFs: mouse embryonic fibroblasts, MDA-MB-231: human breast cancer cell lines, Caco-2: colon carcinoma cell lines, DU-145: prostate cancer cell lines, B16F10: murine melanoma cells, NIH3T3: fibroblast cells, BV2: microglial cells derived from murine, hMSCs: human mesenchymal stem cells, BT-474: human mammary gland breast/ duct carcinoma cell lines, MTT: 3-(4,5-dimethylthiazol-2-yl)-2,5-diphenyltetrazolium bromide assay, CCK-8: cell counting kit-8, FITC: fluorescein isothiocyanate, PI: propidium iodide, TE: trypsin-EDTA, TC10: automated cell counter, SRB: sulforhodamine B assay, HP: hoechst 33, 342 + propidium iodide, CV: crystal violet assay, [a]: mg kg^{-1} of mice, [b]: days, [c]: % proliferation, [d]: ratio of dead to live cells.
